# Three‐Dimensional Model of Human Nicotinamide Nucleotide Transhydrogenase (NNT) and Sequence‐Structure Analysis of its Disease‐Causing Variations

**DOI:** 10.1002/humu.23046

**Published:** 2016-08-08

**Authors:** Louise A Metherell, José Afonso Guerra‐Assunção, Michael J Sternberg, Alessia David

**Affiliations:** ^1^ Centre for Endocrinology, William Harvey Research InstituteQueen Mary University of LondonCharterhouse SquareLondonUK; ^2^ Centre for Molecular Oncology, Barts Cancer InstituteQueen Mary University of LondonCharterhouse SquareLondonUK; ^3^ Centre for Integrative System Biology and BioinformaticsImperial College LondonLondonUK

**Keywords:** NNT, variations, protein structure, familial glucocorticoid deficiency, mitochondrial disease

## Abstract

Defective mitochondrial proteins are emerging as major contributors to human disease. Nicotinamide nucleotide transhydrogenase (NNT), a widely expressed mitochondrial protein, has a crucial role in the defence against oxidative stress. *NNT* variations have recently been reported in patients with familial glucocorticoid deficiency (FGD) and in patients with heart failure. Moreover, knockout animal models suggest that NNT has a major role in diabetes mellitus and obesity. In this study, we used experimental structures of bacterial transhydrogenases to generate a structural model of human NNT (H‐NNT). Structure‐based analysis allowed the identification of H‐NNT residues forming the NAD binding site, the proton canal and the large interaction site on the H‐NNT dimer. In addition, we were able to identify key motifs that allow conformational changes adopted by domain III in relation to its functional status, such as the flexible linker between domains II and III and the salt bridge formed by H‐NNT Arg882 and Asp830. Moreover, integration of sequence and structure data allowed us to study the structural and functional effect of deleterious amino acid substitutions causing FGD and left ventricular non‐compaction cardiomyopathy. In conclusion, interpretation of the function–structure relationship of H‐NNT contributes to our understanding of mitochondrial disorders.

## Introduction

Nicotinamide nucleotide transhydrogenase (NNT, MIM #607878), a widely expressed integral protein of the inner mitochondrial membrane [Arkblad et al., 2002], catalyzes the transhydrogenation between NADH and NADP+ and the proton translocation across the mitochondrial membrane. Because of its role in maintaining the redox balance, NNT has a crucial role in defence against oxidative stress [Circu and Aw 2010]. Variations in *NNT* have recently been reported in patients with familial glucocorticoid deficiency (FGD, MIM#614736), a rare, life‐threatening condition [Meimaridou et al., 2012; Yamaguchi et al., 2013; Novoselova et al., 2015] and in one patient with combined adrenal failure and testicular adrenal rest tumor [Hershkovitz et al., 2015]. Moreover, a reduction in human NNT (H‐NNT) activity has been reported in the heart of patients with heart failure [Sheeran et al., 2010; Bainbridge et al., 2015] and knockout animal models suggest a role for *Nnt* in impaired insulin secretion, diabetes mellitus, and obesity [Freeman et al., 2006; Rydström 2006; Andrikopoulos 2010; Heiker et al., 2013]. Studies in mice with *Nnt* deletion and acute pulmonary infection by *Streptococcus pneumoniae* suggest that Nnt can also be a regulator of the macrophage‐mediated inflammatory response and the defence against pathogens [Ripoll et al., 2012].

The increase in reactive oxygen species resulting from perturbation of the redox balance (oxidative stress) can lead to cell damage and apoptosis [Circu and Aw 2010], which contribute to aging, neurological disorders [Uttara et al., 2009], and cancer [Reuter et al., 2010]. NNT is, thus, a strong candidate in the search for novel genes involved in the pathogenesis of the above conditions. Several novel genetic variations are likely to be identified in *NNT* in the near future because of sequencing efforts such as the 100K genomes project. Understanding the structure and function of NNT is, therefore, of paramount importance in the prioritization and characterization of novel *NNT* genetic variations.

NNT exists as a homodimer [Pedersen et al., 2008]. Each monomeric NNT is characterized by three domains: the NAD(H) binding domain (domain I), the transmembrane domain (domain II), which is responsible for anchoring NNT to the mitochondrial inner membrane and for proton translocation and the NADP(H) binding domain (domain III). Domain I and III are located on the matrix side in mitochondria and on the cytoplasmic side in bacteria [Cotton et al., 2001]. Hydride transfer between NAD(H) and NADP(H) occurs at the interface between domains I and III and is coupled with proton translocation across the proton canal located in domain II [reviewed in [Pedersen et al., 2008]]. The crystal structure of domain I, with and without NAD(H), has been determined in *Rhodospirillum rubrum* [Prasad et al., 2002] and *Escherichia coli* [Johansson et al., 2005], whereas that of domain III in bovine [Prasad et al., 1999] and human transhydrogenase [White et al., 2000]. Moreover, a solved structure of domain I–III complex was determined in *R. rubrum* [Cotton et al., 2001]. A recent breakthrough has been the crystallized 6.9 Å structure of the *Thermus thermophilus* transhydrogenase (*Tt*‐NNT) homodimer [Leung et al., 2015], which allowed clarification of how the three domains are positioned in relation to one another. Moreover, it showed the 180° flipping of domain III relative to domain I, which allows the NAD(H) and NADP(H) binding sites to come in close contact and exchange hydride.

We present a structural model of the H‐NNT protein and a structural analysis of *NNT* deleterious amino acid substitutions causing FGD. Moreover, we present the results of a comparative sequence analysis among different species and structural annotation of evolutionary conserved motifs for the purpose of identifying residues, which are unlikely to tolerate substitutions.

## Materials and Methods

The amino acid sequence of H‐NNT (UniProt  Identification Code (ID) Q13423, GenBank: AAC51914.1), *E. coli* (UniProt IDs P07001 and P0AB67, GenBank: CAB37089.1 and CAB37090.1, respectively) and *T. thermophilus* (UniProt IDs Q72GR8, Q72GR9, Q72GS0, GenBank: AAS82122.1, AAS82121.1, AAS82120.1, respectively) were retrieved from UniProt [UniProt Consortium 2015]. The amino acid numbering system is based on the initiation codon as codon 1, as per the HGVS recommendations.

A structural model of NNT domains was generated using Phyre2 prediction program [Kelley et al., 2015]. Although the structure of the holo Tt‐NNT was available [Leung et al., 2015], its resolution is very low and not appropriate for homology modeling. We, therefore, first modeled individual domains using the best template structures available. Modeling of individual domains was performed automatically using Phyre2 [Kelley et al., 2015], as detailed in Supp. Methods. A list of all templates used to generate the H‐NNT model is presented in Table 1. The amino acid percentage relative surface accessibility area (rASA) was calculated by dividing its total surface area with that in the extended conformation (*ϕ* = *Ψ* = 180°) of the Gly–X–Gly tripeptide. Residues were defined as solvent accessible if rASA > 7%, otherwise buried [Worth and Blundell 2010]. Residues were assigned to interface when the distance between at least one atom on two different interacting proteins was within 5 Å. Interface residues were defined as “interface core” when becoming buried upon interaction, otherwise they were “interface rim” [Chakrabarti and Janin 2002]. Disordered regions were predicted using Disopred2 prediction program [Ward et al., 2004]. 3DLigandSite was used to predict residues interacting with dinucleotide coenzymes [Wass et al., 2010].

**Table 1:** **PDB** Files **used to** Generate **the H‐NNT** Structural Model

PDB file	Ligand	Method	Resolution	Seq Id %	NNT domain	Organism
2bru	NAD, NADP	NMR	–	56	I, I‐I, I‐III	*E. coli*
4izh	Glycerol	X‐ray	1.8 Å	41	I	*T. thermophilus*
4o9p (chains C‐D)	–	X‐ray	2.89 Å	31	II	*T. thermophilus*
1djl	NADP, sulfate ion, glycerol	X‐ray	2.0 Å	100	III	*Homo sapiens*
4o9u	NAD, NADP	X‐ray	6.93 Å	33	Holo‐NNT	*T. thermophilus*

Seq id %, percentage of sequence identity to H‐NNT amino acid sequence; NAD, nicotinamide‐adenine‐dinucleotide; NADP, nicotinamide‐adenine‐dinucleotide phosphate.

For structural analysis, the following structural elements were considered: (i) salt bridges, defined as at least one pair of atoms on oppositely charge groups within a 4.5 Å distance; (ii) hydrogen bonds (H‐bond), defined as a donor–acceptor distance ≤2.5 Å and an angle at the acceptor ≥90°; (iii) pi–pi stacking, defined as an interaction between two aromatic rings, where the maximum distance between the ring centroids is <4.4 Å and the angle between ring planes <30° (face‐to‐face) or the distance is <5.5 Å and >60° angle <120° (edge‐to‐face); (iv) disulfide bridge (S‐S bridge) defined as the side chains of two cysteines at a 3.0 Å distance. Pairs of cysteines at a greater distance were also considered as potentially forming an S‐S bridge when their Cα‐Cα distance was <10 Å. We used Cα distance to allow for errors in side‐chain placement and a relatively high threshold to accommodate possible deviation of the backbone from the native.

The NNT electrostatic potential was calculated using PBEQ program [Jo et al., 2008], which computes the protein electrostatic potential by solving the Poisson–Boltzmann equation. The presence of a signal peptide was investigated using SignalP 4.1 [Petersen et al., 2011]. The presence of a mitochondrial targeting sequence was predicted using TargetP 1.1 [Emanuelsson et al., 2007] and Predotar [Small et al., 2004]. Three variant phenotype prediction servers SIFT [Kumar et al., 2009], Polyphen2 [Adzhubei et al., 2010] and Suspect [Yates et al., 2014] were used to assess the damaging nature of single amino acid variations (SAVs). The logo was generated using Weblogo [Crooks et al., 2004].

The amino acid sequences for *NNT* homologs were retrieved from Uniprot and Ensembl. Homologs were selected among those with high‐coverage sequencing and complete genome assembly, from species spanning a broad evolutionary distance. For bacterial genomes, the sequences of the different elements of the operons were concatenated. The sequences were aligned using the G‐INS‐i algorithm implemented in the MAFFT package (global‐global alignment) [Katoh and Standley 2013]. The alignment robustness was checked using GUIDANCE2 [Penn et al., 2010]. Amino‐acid conservation profiles were analyzed using ConSurf [Ashkenazy et al., 2010]. The sequence alignment was generated using ESPript 3.0 [Robert and Gouet 2014]

## Results

### Overview of Human NNT

H‐NNT is encoded by a single gene, whereas bacteria transhydrogenases are encoded by two (*E. coli*, UniProt IDs P07001 and P0AB67, GenBank: CAB37089.1 and CAB37090.1, respectively) or three genes (*T. thermophilus*, UniProt IDs Q72GR8, Q72GR9, Q72GS0, GenBank: AAS82122.1, AAS82121.1, AAS82120.1, respectively). Nevertheless, the structural organization of the NNT protein is similar across different species, with domain III always at the C‐terminal part of the protein [Cotton et al., 2001; Leung et al., 2015].

**Figure 1:**
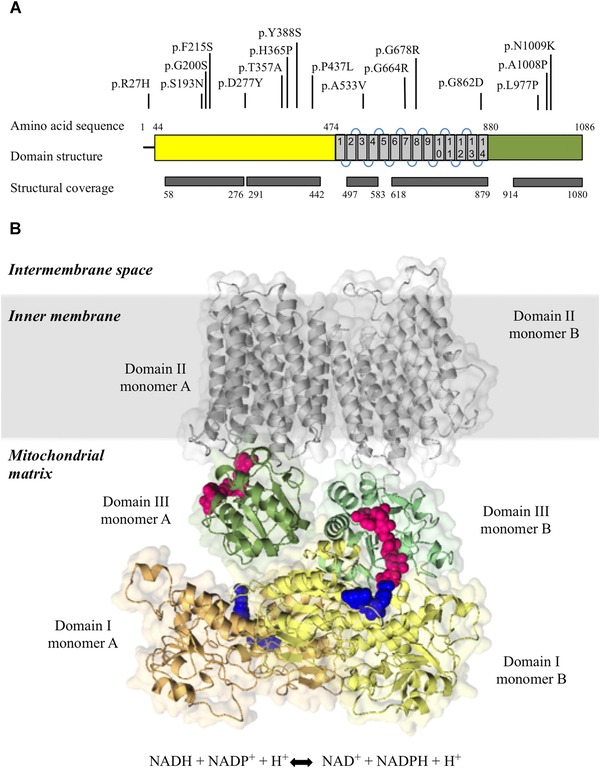
**A**: Domain organization of H‐NNT. Domains are color‐coded (domain I in yellow, domain II in gray and domain III in green). The 14 TMs of domain II are also indicated. The position of deleterious amino acid substitutions identified in H‐NNT and described in this study is presented. The protein regions for which an experimental or homology‐derived structure is available (structural coverage) are also shown. **B**: Three‐dimensional model of H‐NNT homodimer (monomer A and monomer B) inserted in the inner mitochondrial membrane. The reaction catalyzed by NNT is also presented. On the right, domain III (in green) is in the up‐face orientation with the NADP(H) binding site (in magenta) oriented toward domain I (in yellow) and interacting with the NAD(H) binding site (in blue). On the left, domain III (in green) is in the face‐down orientation, with the NADP(H) binding site (in magenta) facing away from the NAD(H) binding site (in blue) and in close proximity to domain II (in gray). H‐NNT dimeric model is based on *Tt*‐NNT (PDB 4O9U).

H‐NNT is comprised of three domains (Fig. 1]: the N‐terminal domain (domain I: residues 1–474) located in the mitochondrial matrix and containing a predicted mitochondrial targeting peptide (1–39 aa) and the NAD binding site; a transmembrane domain (domain II: residues 475–880), which spans the inner mitochondrial membrane and a C‐terminal domain (domain III: residues 881–1086), which contains the NADP binding site.

The structural model of NNT was built by template‐based modeling. A total of 924 (85%) of H‐NNT residues were modeled (for 62 of these residues no coordinates were found in the template and their predicted secondary structure was automatically generated by Phyre2). For 162 residues, no template was available and no secondary structure was generated. Forty‐one of these residues were predicted disordered (the detailed sequence‐structure alignment is presented in Supp. Table S1). Each of the three domains was modeled using a different structural template (detailed in Table 1] and the structure of the individual domains is discussed separately (a multiple sequence alignment (MSA), between NNT and its close homologues is presented in Supp. Fig. S1 and the degree of conservation of the amino‐acid sites between NNT and its close homologues is presented in Supp. Table S2). The Phyre2 confidence score, which reflects the probability that the chosen template is correct, was 100% in all cases.

#### NNT domain I

The 3D structure of domain I (residues Val59‐Lys442) was modeled using the NAD transhydrogenase experimental structures of *E. coli* (PDB: 2bru, sequence identity with human sequence = 56%). This was preferred to that of *T. thermophilus* (PDB: 4izh, sequence identity = 41%) because of the higher sequence identity to H‐NNT. The quality of the model was overall good (PROCHECK Ramachandran statistics: 1% in disallowed regions, 85% residues in favored regions and 14% in additionally allowed regions; ProSA Z‐score −8.48, which is within the range of scores typically found for native proteins of similar size; Supp. Fig. S2). Superposition between H‐NNT domain I and *E. coli* NNT template demonstrated that the two structures have the same overall conformation with minimal structural differences (r.m.s.d. = 0.60 Å).

Several salt bridges, which are likely to contribute to structural stabilization of domain I, were identified: Glu64‐Lys127, Lys127‐Glu141, and Asp312‐Lys338, which are also present in the *E. coli* template structure and Arg254‐Glu310 and His210‐Glu361, which appear to be specific to H‐NNT. A structural analysis demonstrated the presence of a beta‐alpha‐beta fold (Rossmann fold), which is typically present in domains binding dinucleotide coenzymes [Kutzenko et al., 1998]. In particular, residues 234–239 formed the consensus sequence Gly‐X‐Gly‐X‐X‐Gly located at the beta‐alpha junction of the beta‐alpha‐beta fold [Hanukoglu 2015].

**Figure 2:**
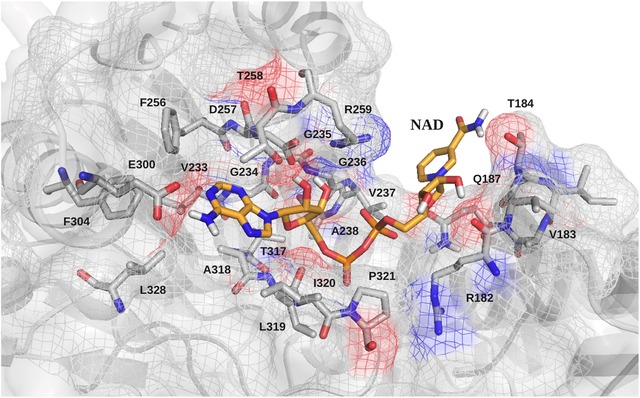
NAD binding site in H‐NNT domain I. The 3D model structure of H‐NNT was used to identify residues likely to participate in NAD binding.

NAD was modeled with H‐NNT domain I using 3Dligandsite and residues that are predicted to interact with NAD are presented in Figure 2 and Supp. Figure S3. The conformational changes occurring at the NAD binding site (as described below), do not allow excluding that additional H‐NNT residues may participate in NAD binding. Amino acids 319–323 (residues LIPGK, which are located within a loop) and Arg182 are conserved between human and *E. coli*, as shown by sequence alignment (Supp. Figures S4A and S4B). Studies in *E. coli* show that these residues are crucial for NNT function. Comparison of experimental structures of *E. coli* domain I, determined both in the absence and presence of NAD and NADH, demonstrate that the side chains of Arg120, Leu257, and Pro262 (corresponding to Arg182, Leu319, and Pro321 in H‐NNT, respectively) adopt different conformations according to the presence or absence of NAD and NADH. When domain I is bound to NAD, the side chain of Arg120 points toward NAD and makes strong interaction with it, whereas in the Apo Domain I (with no substrate) and in the Domain I‐NADH complex, the side chain points in the opposite direction, away from NADH. Similarly, Leu257 and Pro262 can adopt at least three different conformations, according to presence of NAD or NADH [Johansson et al., 2005]. Residues 181–194 form the so‐called RQD loop. Residues Arg182, Val183, Thr184, and Gln187 are evolutionarily conserved and are predicted to interact with NAD (Fig. 2). In addition, Asp190 is located at 3.8 Å from NAD C4 atom and could participate in NAD binding. Ser193 is also an invariable residue and is part of the enzyme catalytic site, which also involves the invariable Arg182, Gln187, and Asp190 (Supp. Fig. S5).

Residues 211–229 are arranged in a beta hairpin fold. Residues Phe211 and Phe215 are evolutionarily conserved, as demonstrated by the MSA and are predicted to form stacking interactions, thus, stabilizing the beta hairpin structure. The beta hairpin fold is a conserved structural feature of NNT as it is present in bacterial transhydrogenases. This suggests that the beta hairpin fold is involved in human NNT catalytic activity, similar to what is observed in bacteria [Johansson et al., 2005]. In addition, the beta hairpin fold participates in domain I homodimerization, as detailed below.

The last 35 amino acids of domain I (residues 437–472) are predicted to be disordered and no alpha helices or beta strands regions were predicted. This short polypeptide is likely to represent a linker between domains I and II. The linker is likely to be flexible, with the exception of its initial sequence (residues Pro437, Arg438, Pro439, Thr440, and Pro441), which is predicted to have a rigid structure. The MSA showed that residues P437, A438, and P439 are highly conserved among different species.

#### NNT domain II

Different species have different numbers of the transmembrane α helices (TM) in the transmembrane domain (domain II): 14 TMs in H‐NNT, 13 TMs in *E. coli*, and 12 TMs in *Tt*‐NNT. H‐NNT domain II (residues Leu496–Met581 corresponding to TMs 2–4 and residues Asn618–Ile888 corresponding to TMs 6–14) was modeled using the *Tt*‐NNT structure (PDB 4o9p, sequence identity to human protein = 33%; r.m.s.d. between template and model = 0.63 Å). This allowed modeling of 349 out of 405 (86%) residues. TM1 and TM5 were not modeled due to the presence of only twelve TM helices in *Tt*‐NNT (a cartoon of H‐NNT domain II is presented in Supp. Fig. S6). The model accuracy was overall good (PROCHECK: Ramachandran statistics: 0% in disallowed regions, 94% residues in favored regions and 5% in allowed regions; ProSA Z‐score −4.89, which is within the range of scores typically found for native proteins of similar size; see Supp. Fig. S7).

One of the most important features of domain II is the presence of the proton translocation canal. In *T. thermophilus*, the proton canal is formed by TM helices 2, 3, 7, 8, 11, 12, and has a hexagonal topology [Leung et al., 2015]. The same hexagonal topology is seen in the H‐NNT proton translocation canal, which is formed by TM helices 3, 4, 9, 10, 13, and 14.

Mutagenesis studies in *E. coli* have identified residues within the proton canal that are essential for proton translocation through the mitochondrial membrane. Structural alignment between the human model and the bacterial templates allowed mapping and identification of these functionally and structurally important residues in H‐NNT. Residues His707 (in TM9), Ser756 (in TM10), and Asn839 (in TM13) form part of the canal's interior face. They are highly conserved and correspond to residues His89, Ser139 and Asn222 in *E. coli*, respectively. Mutational studies in *E. coli* showed that these residues are essential for proton translocation activity [Holmberg et al., 1994; Bragg and Hou 2001]. This crucial function is likely to be retained by the corresponding residues in H‐NNT. Alignment of TM helices forming the proton canal showed a high level of conservation at sequence and structural level. The conserved His524 (H450 in *E. coli*) is part of the loop between TM2 and TM3, which is located in the mitochondrial matrix. In *E. coli*, its substitution results in a markedly reduced proton translocation activity [Holmberg et al., 1994]. An additional residue, which, when substituted, inhibits the enzyme function possibly by altering its structure, is the conserved Met876 (Met259 in the beta subunit of *E. coli*) [Karlsson et al., 2003]. Although the majority of substitutions result in an inactive enzyme, it is worth noticing that substitution of the conserved Ser867, Ser868, and Ser873 (Ser250, Ser251, and Ser256 in *E. coli*, respectively) located in TM14 have been shown to enhance NNT activity possibly through an allosteric effect mediated by TM14 on NADP binding [Yamaguchi and Stout 2003; Karlsson et al., 2003].

Inhibition of NNT function can also result from amino acid substitutions that affect the tight packing of domain II alpha helices, which is driven by the interaction between the side chains of glycine and isoleucine/valine (the so called “groove‐ridge system”). Because of its special role in the packing of helices, glycine substitution with any other amino acid can result in profound structural modification and NNT malfunction. Accordingly, in vitro studies have demonstrated that substitution of the invariant Gly711 (TM9), Gly749 (TM10), Gly755 (TM10), Gly843 (TM13), Gly850 (TM13) impairs proton translocation and transhydrogenation activity (Gly95, Gly132, Gly138, Gly226, Gly233 in *E. coli*, respectively) [Yamaguchi and Stout 2003]. Moreover, substitution of the invariant Gly862, Gly866, and Gly869 (Gly245, Gly249, and Gly252 in beta *E. coli*, respectively), which form the last TM helix (TM14, which completes the proton translocation canal hexagonal structure), has been shown to reduce NNT function [Yamaguchi and Stout 2003; Karlsson et al., 2003], possibly through disruption of the groove‐ridge system, which contributes to tight packing of TM14 against TM13 in human and bacterial transhydrogenases.

A partially disordered linker connects domain II and III (residues 880–912, of which residues 893–912 are predicted to be disordered). The disordered nature of the linker allows the 180° flip of domain III relative to domain I and II described by Leung et al. (2015). Of great interest are the highly conserved residues Asp830 (located in the cytoplasmic loop of domain II, between TMs 12–13, at the opening of the proton canal toward the mitochondrial matrix) and Arg882 (located within the linker). These two residues form a salt bridge, which is a conserved feature across species (a salt bridge is formed by equivalent residues Asp202 and Arg254 in *Tt*‐NNT [Leung et al., 2015] and βAsp213‐βArg265 in *E. coli* [Althage et al., 2001]. In line with the conformational changes adopted by domain III in relation to its functional status, residues Asp830 and Arg882 have been shown to interact in the absence of NADP, but not in its presence [Althage et al., 2001]. Moreover, substitution of equivalent residues in *E. coli* alters NNT proton translocation activity [Yamaguchi and Hatefi 1995]. The holo *Tt*‐NNT solved with NADP [Leung et al., 2015] shows that the salt bridge formed by these two residues is adjacent to the entrance of the proton canal and in the face‐down conformation (which is competent for proton translocation), the NADP binding site in domain III comes in close contact with the salt bridge and the proton canal. Residues Asp830 and Arg882 in the H‐NNT are likely to have the same crucial functional and structural role as described for the equivalent residues in *Tt*‐NNT and *E. coli*.

#### NNT domain III

The NNT domain III (residues Pro902–Ser1083) bound to NADP was crystallized from human heart mitochondria in 2000 (PDB id: 1djl) [Peake et al., 2000; White et al., 2000] and an extensive structural analysis of this domain is described by White et al. (2000). We will therefore limit our analysis to the identification of structurally and/or functionally important residues in this domain.

**Figure 3:**
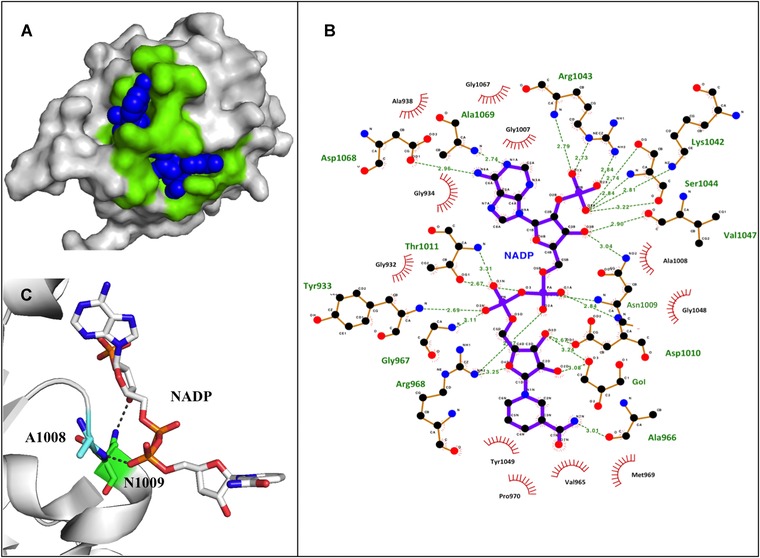
NADP ligand binding site. **A**: 3D structure of domain III in H‐NNT. The NADP cleft is presented in green and NADP as blue spheres. **B**: Schematic 2D representation of domain III residues interacting with NADP. The diagram was generated with Ligplot+ using the human structure of domain III (PDB 1djl). The bold purple bonds belong to the ligand, whereas the thin brown bonds to H‐NNT. Hydrogen bonds between H‐NNT and NADP are presented as dashed lines. H‐NNT residues making hydrophobic contact are presented as spiked arcs. **C**: Residues Ala1008 and Asn1009, which harbor variations p.Ala1008Pro and p.Asn1009Lys, interact with NADP. H‐NNT residues have been renumbered to follow NNT amino acid numbering reported in Uniprot. The alignment between PDB 1djl and H‐NNT Uniprot sequence is available at: http://www.ebi.ac.uk/thornton-srv/databases/cgi-bin/pdbsum/GetPage.pl?pdbcode=1djl&template=align.html&l=1.

Five salt bridges can be identified in this domain and are likely to help stabilize its structure (Asp915–Lys1079, His995–Asp996, Glu1018–Lys1059, Glu1031–Lys1034, Lys1070–Asp1074). Structurally, the NADP binding site is characterized by the Rossmann fold and residues 932–937 form the consensus sequence Gly‐X‐Gly‐X‐X‐Ala/Val. The residues forming the NADP cleft, which makes contact with NADP, are shown in Figure 3.

#### H‐NNT dimeric structure and open challenges

**Figure 4:**
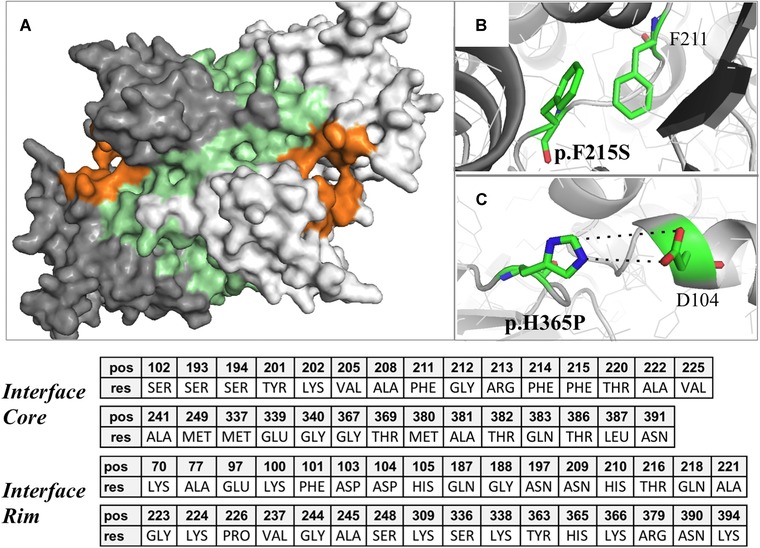
The predicted H‐NNT domain I interface site and amino acid substitutions causing glucocorticoid deficiency. **A**: Surface representation of the large interface site (in green) formed by two domain I monomers (presented in light and dark gray, respectively). The NAD binding sites, one on each monomer, are presented in orange. **B**: Residue His365, which harbors the p.His365Pro substitution, is a rim interface residue and is predicted to interact with Asp104 on the opposite domain I. **C**: Phe215, which harbors the p.Phe215Ser substitution, is a conserved interface core residue. Phe215 is predicted to form stacking interactions with Phe211 on the same chain. The name (res) and position on the NNT amino acid sequence (pos) of residues predicted to form the core and rim of dimer I interface is also presented.

NNT assembles in the inner mitochondrial membrane as an asymmetric homodimer [Leung et al., 2015]. We used the H‐NNT model to identify the homodimerization interface sites for domain I. In order to construct the homodimer structure for H‐NNT domain I, the *E. coli* domain I complex was used as a template (PDB: 2bru, dI_2_‐dIII structure). Two monomeric chains of H‐NNT were superposed to the *E. coli* dimeric structure and rotated using the *E. coli* complex coordinates. Superposition of equivalent domain I C alpha atoms (r.m.s.d. = 0.80 Å) between human and *E. coli* NNT, demonstrated a high level of similarity between model and template. As expected, manual comparative analysis showed that the highest level of structural divergence was located within loop regions. The model allowed calculation of the 3D atomic coordinates of residues forming the domain I‐domain I interface site for H‐NNT. Residues forming this large protein–protein interface and their conservation score are reported in Figure 4. The majority of residues forming the beta hairpin fold were shown to be part of this large interface (Fig. 4A). Among these, Phe211, Gly212, Phe214, Phe215, Ala222, and Ala228 were predicted to be interface core residues, as they shifted from solvent accessible to fully buried (solvent inaccessible) upon dimer formation. Identification of interface core residues is of particular relevance, since they play a major role in protein‐protein interaction and binding affinity [Chakrabarti and Janin 2002] and are a hot spot for disease causing variations [David et al., 2012]. Although mutational studies in *E. coli* have shown that deletion of the beta hairpin does not impair the formation of domain I dimer [Johansson et al., 2005], the interface residues, which are part of the beta hairpin, could still play a role in modulating the affinity of domain I dimer formation.

The NNT transmembrane domain (domain II) has been crystallized as a dimer in *T. thermophilus* [Leung et al., 2015]. We generated the H‐NNT domain II dimer using bacterial transhydrogenase 3D coordinates as a template (PDB 4o9t, sequence identity 34%). Sequence and structural alignment showed that H‐NNT TM2, 3 and 4 correspond to *Tt*‐NNT TM1‐2‐3. Structural superposition between model and template shows that several residues on opposite TM1s in *Tt*‐NNT and the equivalent TM2s in H‐NNT can potentially take part in hydrophobic interactions. Nevertheless, the presence of two additional helices in the H‐NNT (TM1 and TM5, which could not be modeled due to the absence of a suitable template) could result in different placement of TM2 within the inner mitochondrial membrane and therefore a different role in domain II dimerization. Moreover, participation of additional residues, located on TM1 and/or TM5, to H‐NNT domain II interface site cannot be excluded. A cartoon of the *E. coli* NNT domain II (which contains 13 TM helices) generated in 2001 based on literature data, suggests that TM1 may be located next to TM2, thus in close proximity to domain II dimerization site [Meuller et al., 2001]. Nevertheless, in this cartoon, the spatial location of the helices surrounding the proton canal appears to be different from what reported by Leung et al. using the solved structure of *Tt*‐NNT [Leung et al., 2015]. In particular, TM4 appears not to contribute to the hexagonal topology of the proton canal. Because of this discrepancy, and in the absence of a solved structure for *E. coli* domain II, it is difficult to speculate on the location of TM1 in relation to other helices and to the dimerization site in H‐NNT.

#### Sequence and structural analysis of NNT deleterious variations

The availability of the H‐NNT model allowed analysis of the structural/functional consequences of deleterious NNT amino acid substitutions and their predicted effect on H‐NNT is detailed below and in Table 2, Supp. Figure S1, and Supp. Figure S8A‐Q.

**Table 2:** Conservation Score and Prediction Scores from Three Variant Prediction Servers for H‐NNT Deleterious Amino Acid Substitutions and Rare Genetic Variations Reported in the EXAC Database

Genetic variation	Conservation score	Most common amino acids in the alignment	SIFT	Polyphen2	Suspect	Structural analysis	Figure
p.Arg27His	1	Pro(50.0%), Arg(20.0%), Thr, Lys and His (10.0%)	0.13 T	0.000 T	24 T	n.a.	n.a.
p.Lys63Arg	4	Lys(77.8%), Arg(16.7%), Gln(5.6%)	0.47 T	0.895 Poss D	28 T	Likely tolerated	S Fig 8A
p.Ser193Asn	9	Ser(100.0%)	0 D	0.754 Poss D	60 D	Likely deleterious	S Fig 5
p.Gly200Ser	9	Gly(100.0%)	0.02 D	0.252 T	90 D	Likely deleterious	S Fig 8B
p.Phe215Ser	9	Phe(100.0%)	0 D	1.00 D	47 Poss D	Likely deleterious	S Fig 8C
p.Asp277Tyr	5	Asp(77.8%), Asn(16.7%), Pro(5.6%)	0 D	0.686 Poss D	62 D	n.a.	n.a.
p.Thr357Ala	8	Thr(88.9%), Ser(5.6%), Ile(5.6%)	0 D	0.204 T	75 D	Likely deleterious	S Fig 8D
p.His365Pro	1	His(61.1%), Val and Lys(11.1%), Tyr, Glu and Ala(5.6%)	0.27 T	0.915 Poss D	19 T	Likely deleterious	S Fig 8E
p.Tyr388Ser	7	Tyr(94.4%), Phe(5.6%)	0 D	1.00 D	85 D	Likely deleterious	S Fig 8F
p.Pro437Leu	7	Pro(94.4%), Gly(5.6%)	0 D	0.942 Poss D	77 D	Likely deleterious	S Fig 8G
p.Ala533Val	8	Ala(94.4%), Phe(5.6%)	0 D	0.986 D	80 D	Likely deleterious	S Fig 8H
p.Thr589Ser	2*	Thr(62.5%), Asp(25.0%), Gln(6.3%), Pro(6.3%)	0.09 T	0.376 T	15 T	n.a.	n.a.
p.Leu663Phe	5	Gly(77.8%), Phe(11.1%), Val(5.6%), Leu(5.6%)	0.01 D	1.00 D	24 T	Likely tolerated	S Fig 8I
p.Gly664Arg	8	Gly(100.0%)	0 D	1.00 D	56 D	Likely deleterious	S Fig 8J
p.Gly678Arg	1	Gly(61.1%), Leu(22.2%), Thr, Ser and Ala(5.6%)	0.02 D	0.841 Poss D	56 D	Likely deleterious	S Fig 8K
p.Thr731Met	1	Thr(52.9%), Glu(23.5%), Ala(11.8%), Met and Leu(5.9%)	0.07 T	0.035 T	21 T	Likely tolerated	S Fig 8L
p.Gly862Asp	9	Gly(100.0%)	0 D	1.00 D	87 D	Likely deleterious	S Fig 8M
p.Leu977Pro	9	Leu(100.0%)	0 D	1.00 D	92 D	Likely deleterious	S Fig 8N
p.Ile993Val	9	Ile(94.4%), Leu(5.6%)	0.04 D	0.804 poss D	35 T	Likely tolerated	S Fig 8O
p.Ala1008Pro	9	Ala(94.4%), Ser(5.6%)	0 D	1.00 D	94 D	Likely deleterious	S Fig 8P
p.Asn1009Lys	9	Asn(100.0%)	0 D	1.00 D	84 D	Likely deleterious	S Fig 8Q

D, deleterious; Poss D, possibly deleterious; T, tolerated. Score is predicted deleterious if <0.05 for SIFT and ≥50 for Suspect. Polyphen2 calculates the naïve Bayesian posterior probability that a mutation is deleterious and classifies it accordingly, in “possibly” or “probably” deleterious or tolerated. *, unreliable estimate due to high number of gaps in alignment. S Fig, Supp. Figure.

**p.Ser193Asn,** homozygous in FGD patient [Meimaridou et al., 2012]. Ser193 is an invariable residue part of the enzyme catalytic site of the proton‐translocating transhydrogenase, which also involves the invariable residues Arg182, Gln187, and Asp190 (Supp. Fig. S5). Substitution of Ser138 in *R. rubrum*, which is equivalent to Ser193 in H‐NNT, leads to inhibition of the transhydrogenation rate without changing the binding affinity of the transhydrogenase to NAD [Brondijk et al., 2006].

**p.Gly200Ser,** homozygous in patients with combined mineralocorticoid and glucocorticoid deficiency [Weinberg‐Shukron et al., 2015]. Position 200 is an invariant Gly. It is located in an alpha helix, tightly packed against the alpha helix of Rossmann fold in domain I. Substitution of Gly to Ser is predicted to create a steric clash with this important structural motif. Moreover, this substitution introduces a hydrophilic residue in an otherwise hydrophobic environment.

**p.Phe215Ser,** homozygous in FGD patient [Yamaguchi et al., 2013]. Phe215 is a core residue of the dimerization interface of domain I. It is at the beginning of the beta hairpin structural motif. Phe215 stabilizes bacterial and human NNT structure by forming a stacking interaction with Phe211 on the same chain (Fig. 4B). Phe is a large, hydrophobic amino acid. Its substitution with serine would introduce a polar and much smaller amino acid, not able to form stacking interactions. This substitution is predicted to affect the H‐NNT structure and in particular the dimerization site.

**p.Asp277Tyr,** identified in a patient with left ventricular non‐compaction (LVNC, MIM#604169) [Bainbridge et al., 2015]. This position is predicted to be part of a loop in domain I. The loop in human NNT is longer than the corresponding loop in bacterial transhydrogenase and therefore a structural analysis could not be performed. Position 227 is not conserved, nevertheless tyrosine is never observed in the MSA. This substitution is predicted to be deleterious by prediction programs (Table 2) and functional studies demonstrated that it causes a partial loss in NNT function in Zebrafish larvae [Bainbridge et al., 2015].

**p.Thr357Ala,** found in compound heterozygosity in an FGD patient in addition to p.Met880Ter, which is predicted to cause nonsense‐mediated NNT mRNA decay [Meimaridou et al., 2012]. Thr357 is a hydrophilic, highly conserved residue on the surface of bacterial and human NNT and participates in hydrogen bonding. Its substitution with the hydrophobic alanine disrupts the H bonding. This substitution is predicted damaging by SIFT and Suspect (scores 0 and 75, respectively) but not by Polyphen2 (score = 0.204).

**p.His365Pro,** homozygous in an FGD patient [Meimaridou et al., 2012]. His365 is part of H‐NNT domain I homodimerization site. It is an interface rim residue. His365 is likely to form a salt bridge with Asp104 on the other chain of the dimer (Fig. 4C). His365 is part of a loop and its substitution with proline is likely to be structurally damaging, as it prevents formation of the salt bridge and can introduce a rigid structure in an otherwise short flexible motif. Interestingly, although His365 and Asp104 are not conserved, the salt bridge at this position is. In *E. coli*, a salt bridge can be formed by residues Glu304 and Lys48 corresponding to His365 and Asp104, respectively. This substitution is predicted possibly damaging by Polyphen2 but benign by SIFT and Suspect (Table 2).

**p.Tyr388Ser,** reported in a patient with combined adrenal failure and testicular adrenal rest tumor [Hershkovitz et al., 2015]. Tyr388 is located in domain I outside the NAD binding cleft and the dimerization site. Tyr388 is likely to form a stacking interaction with Phe154, thus helping stabilizing the structure of domain I, similarly to what is observed in bacterial NNT (Tyr327 and Phe92, respectively). Substitution of tyrosine with serine is predicted to alter the structural stability of domain I.

**p.Pro437Leu,** found in compound heterozygosity in an FGD patient in addition to p.Gln557Ter, which is predicted to cause nonsense‐mediated NNT mRNA decay [Meimaridou et al., 2012]. Pro437 is part of the highly conserved amino acid sequence PAP in the linker between domains I and II. Substitution with any other amino acid is likely to be structurally and functionally damaging.

**p.Ala533Val,** homozygous in an FGD patient [Meimaridou et al., 2012]. Ala553 is a highly conserved residue and contributes to the formation of an alpha helix (TM3 in domain II). Substitution with valine is predicted to cause structural damage, as valine is a poor alpha helix forming residue [Gregoret and Sauer 1998]. Moreover, substitution with valine is predicted to create a steric clash with the surrounding residues.

**p.Gly664Arg,** found in compound heterozygosity in an FGD patient in addition to p.Thr689LeufsTer320, which is predicted to cause nonsense‐mediated NNT mRNA decay [Meimaridou et al., 2012]. Gly664 is an evolutionarily conserved residue in TM7 of the NNT transmembrane domain. This variation replaces the smallest in size, hydrophobic, neutral residue with the largest in size, hydrophilic, charged residue. These large physico‐chemical changes are likely to be structurally damaging, especially since this amino acid change occurs in a hydrophobic environment.

**p.Gly678Arg,** found in a patient with FGD in compound heterozygosity with the p.Gly862Asp change described below [Meimaridou et al., 2012]. Gly678 is located in TM8 of the transmembrane domain. The MSA shows that Gly678 is not evolutionarily conserved. Nevertheless, this position is generally occupied by hydrophobic residues (valine, leucine, glycine, and alanine), which is consistent with the hydrophobic environment in which this residue is located. Similarly to the previous variation, Gly678Arg is therefore predicted to be structurally damaging.

**p.Gly862Asp** Gly862 is an invariant residue located in the TM14 of the transmembrane domain. It is part of an alpha helix, which is tightly packed with other TMs. Substitution between glycine and aspartic acid introduces major physico‐chemical changes (aspartic acid is a charged, hydrophilic residue). Moreover, introduction of aspartic acid is likely to create a steric clash with nearby TMs and, thus, structural damaging.

**p.Leu977Pro,** homozygous in an FGD patient [Meimaridou et al., 2012]. Leu977 is a highly conserved residue, located in domain III, outside the NAD binding site. Leu977 is part of an alpha helix and its substitution with proline is predicted to cause a kink in the alpha helix and loss of stability, causing structural damage to domain III.

**p.Ala1008Pro** and **p.Asn1009Lys**. Both variations were found in patients with FGD: p.Ala1008Pro was present in homozygosity, whereas p.Asn1009Lys was in compound heterozygosity with p.His370Ter, which is predicted to cause nonsense‐mediated mRNA decay [Meimaridou et al., 2012]. Ala1008 and Asn1009 are invariant residues located at the NADP cleft in H‐NNT domain III, directly interacting with NADP (Fig. 3B and C). Substitution of these crucial amino acids is predicted to alter NADP binding, thus, greatly affecting H‐NNT function.

#### Sequence and structural analysis of NNT rare SAVs

The following rare variants (detailed in Tables 2 and 3) have been identified in homozygosity in the 1000 Genomes Project [1000 Genomes Project Consortium et al., 2015] and are reported in the EXAC database [Consortium et al., 2015]:

**Table 3:** Rare Amino Acid Substitutions Identified in Homozygosity in the 1000Genomes Project

Protein consequence	Transcript consequence	Filter	Annotation	Allele count	Allele number	Number of homozygotes
p.Lys63Arg	ENST00000264663.5:c.188A > G	PASS	Missense	5,565	121100	196
p.Leu663Phe	ENST00000264663.5:c.1987C > T	PASS	Missense	5,425	121298	188
p.Ile993Val	ENST00000264663.5:c.2977A > G	PASS	Missense	1,107	120944	7
p.Thr731Met	ENST00000264663.5:c.2192C > T	PASS	Missense	897	121348	4
p.Arg27His	ENST00000264663.5:c.80G > A	PASS	Missense	197	121362	2
p.Thr589Ser	ENST00000264663.5:c.1765A > T	PASS	Missense	17	121412	1

***p.Arg27His (rs34241095)*** Arg27 could not be mapped onto the H‐NNT structure. The initial 39 amino acids in H‐NNT may represent the mitochondrial targeting peptide. This short sequence is not present in bacterial transhydrogenases. Position 27 is not conserved and p.Arg27His is predicted benign by most prediction programs (Polyphen2 = 0, SIFT = 0.13, Suspect = 24). Nevertheless, one prediction program (TargetP) suggests that this substitution may affect the mitochondrial targeting peptide. The latter has been shown to be enriched in positively charged and hydroxylated residues, but no clear consensus sequence is known to date [Habib et al., 2007]. Since the mitochondrial targeting peptide is not well characterized, it is not possible to make a prediction on the neutral or deleterious nature of this amino acid change.

***p.Lys63Arg (rs35201656)*** Lys63 is a positive surface amino acid. The position in not conserved and can be occupied by arginine (Supp. Table S2). Lys63 is neither in domain I dimerization site, nor in proximity to NAD binding site. The p.Lys63Arg substitution is likely to be tolerated (SIFT = 0.47 tolerated, Suspect = 28 tolerated, Polyphen2 = 0.895 damaging).

***p.Thr589Ser (rs370370846)*** Thr589 is part of a loop located in the mitochondrial matrix between helices TM4 and TM5. The loop in human NNT is longer than the corresponding loop in bacterial transhydrogenase. This position was not modeled in the H‐NNT model and could not be structurally analyzed. Threonine and serine have similar chemical properties (polar, non‐charged, and capable of forming hydrogen bonds) and are often interchangeable. Threonine to serine substitution is predicted tolerated by most programs (Polyphen2 = 0.376 tolerated, SIFT = 0.09 tolerated, Suspect = 15 tolerated).

***p.Leu663Phe (rs41271083)*** Leu663 is in the TM7 alpha helix of the transmembrane domain. Its substitution with phenylalanine is predicted damaging (Polyphen2 = 1 damaging, SIFT = 0.01 damaging, Suspect = 24 tolerated). Nevertheless, leucine and phenylalanine are amino acids with similar chemical properties (hydrophobic, not charged amino acids) and the MSA shows that, although this position is generally occupied by leucine, other residues such as isoleucine and phenylalanine, can also be present (Supp. Table S2). The lack of all 14 TM helices in the H‐NNT model does not allow excluding that this substitution could affect packing of TM7 with TM1 or TM5 in H‐NNT.

***p.Thr731Met (rs75710404)*** Thr731 is part of a loop located in the intermembrane mitochondrial space between helices TM9 and TM10 and it is unlikely to cause any structural damage. Methionine can be found at this position in the MSA and the threonine to methionine substitution is predicted benign by prediction programs (Polyphen2 = 0.035 tolerated, SIFT = 0.07 tolerated, Suspect = 21 tolerated).

***p.Ile993Val (rs78818665)*** Ile993 is located within an alpha helix in domain III. It is not in proximity to the NADP cleft and its substitution is not predicted to affect NADP binding. Two prediction programs predict this substitution to be not tolerated (Polyphen2 = 0.804 possibly damaging, SIFT = 0.04 damaging, Suspect = 35 tolerated). Nevertheless, isoleucine and valine have similar chemical properties and valine can be seen, although rarely, at this position in the MSA.

## Discussion

This study describes the first structural model of the human NNT, an enzyme crucial in the defence of cells against oxidative stress, defects in which have not only been linked to FGD and LVNC, but are also strong candidates for several other human disorders [Freeman et al., 2006; Reuter et al., 2010; Ripoll et al., 2012]. The 3D model was used to identify functional and structural H‐NNT key motifs and gain essential insight into the structural and functional effect of deleterious amino acid substitutions causing glucocorticoid deficiency and LVNC cardiomyopathy, as well as rare homozygote amino acid variations.

NNT is widely expressed and is likely to contribute to the pathogenesis of a wide range of medical conditions ranging from aging to cancer [Uttara et al., 2009; Reuter et al., 2010]. Therefore, identification of residues, which represent susceptible positions for disease‐causing variations, is important. Although amino acid evolutionary conservation is a good indicator of the structural and functional importance of residues, it cannot inform us on the effects of amino acid substitutions on a biological system. One fourth of amino acid variations in the human genome are predicted to be deleterious by the most widely SAV prediction tools [Yue and Moult 2006; Allali‐Hassani et al., 2009; Adzhubei et al., 2010]. As an amino acid substitution may alter protein fitness by affecting its folding, its location within the cell, its interaction with other molecules, or its ligand binding and catalytic activity [Yates and Sternberg 2013; Stefl et al., 2013], additional methods are required for informing SAV prioritization.

Individual transhydrogenase domains as well as the domain I–domain III complex have been determined in bacteria [Bergkvist et al., 2000; Prasad et al., 2002] and bovine NNT [Prasad et al., 1999]. Availability of 3D structures for individual domains and complexes allowed us to generate a model for H‐NNT and to demonstrate how amino acid substitutions affect H‐NNT fitness through a wide range of functional and structural mechanisms.

Without the H‐NNT 3D model, molecular mechanisms could only be identified for two variations (p.Ala1008Pro and p.Asn1009Lys), which are located in the NADP binding site. The H‐NNT 3D model allowed predictions of residues forming the NAD cleft. This would not have been possible without the aid of a 3D model. As NNT has a crucial role in the redox process, characterization of the NAD cleft becomes of paramount importance toward the understanding of NNT function and to guide in vitro studies. Knowledge of the residues predicted to form the NAD cleft also allowed characterization of variations, such as Ser193Asn.

Another important example of the importance of H‐NNT 3D model was the ability to identify domain I interface site. Disruption of protein–protein interaction, which includes the proteins ability to dimerize, has been recently demonstrated to be an important mechanism in human disease [David et al., 2012; Nishi et al., 2013; Das et al., 2014]. We showed that H‐NNT dimer has a large interface region and at least two NNT variations may affect NNT function by altering its dimerization site. In particular, we dissected the domain I interface into “core” and “rim” residues. Core residues are of great importance in establishing and maintaining protein–protein interaction and their substitution is unlikely to be tolerated [David and Sternberg 2015], as in the case of p.Phe215Ser.

Another important mechanism by which deleterious amino acid substitutions impair NNT function is the disruption of its ability to correctly fold within the mitochondrial inner membrane. This was likely to be the case for three variations in domain II (p.Gly664Arg, p.Gly678Arg, and p.Gly862Asp). We predicted that these substitutions would alter the “groove‐hinge” system, which represents the basis for transmembrane helices packing.

A crucial feature of NNT is the ability of domain III to flip according to NNT functional state (proton translocation across the membrane or hydrogen transfer). [Leung et al. 2015] demonstrated in the structure of bacterial transhydrogenase (which is localized in the cytosol) that domain III cycles from an up‐face orientation (NADP binding site oriented toward domain I and interacting with NAD binding site) to a face down orientation (NADP binding site oriented toward domain II, thus away from NAD binding site). The opposite phenomenon would occur in the second monomer, thus conferring an asymmetric structure to the NNT dimer. Moreover, they proposed that different orientation of domain III are associated with modifications in domain II proton canal orientation (inward or outward facing) and its ability to translocate protons across the mitochondrial membrane. The high level of structural similarity between the bacterial transhydrogenase structure and the human NNT model, suggests that the same mechanism is likely to occur in the human NNT dimer, as previously suggested [Krengel and Törnroth‐Horsefield 2015].

The flipping of domain III requires the absence of a rigid structure in the linker between domain II and III. Our structural analysis reveals that the amino acid sequence of this linker is highly disordered, thus supporting the hypothesis that this region is highly flexible. Identification of the amino acid sequence forming the linker in human NNT would not have been possible without the availability of a 3D model. Moreover, understanding the physical properties of the linker is of particular importance when analyzing amino acid substitution occurring in this region. There is mounting evidence of the role of intrinsically disorder regions (IDRs) in human disease [Uversky et al., 2008]. Amino acid substitutions occurring in the hinge should be evaluated for their propensity to create a disorder‐to‐order transition, which would affect the hinge flexibility, as it is often the case in deleterious mutations occurring in IDRs [Dunker et al., 2008]. Although at the moment no *NNT* deleterious variations are known to occur in this region, identifications of potentially deleterious amino acid substitutions in the hinge may occur in the future.

In conclusion, structural biology can provide valuable information on protein structure–function relationship and integration of genetic analysis with protein 3D modeling can greatly enhance prioritization and interpretation of genetic variants. Analysis of H‐NNT 3D model and structural interpretation of its deleterious amino acid substitutions, represent a powerful example. Moreover, availability of H‐NNT 3D model and identification of key structural/functional residues will prove valuable, as several novel NNT genetic variations are likely to be identified in the near future, not only as a cause of adrenal disorders, but as a risk factor for a wide range of conditions, such as aging, inflammatory response, and cancer.

## Supporting information

Disclaimer: Supplementary materials have been peer‐reviewed but not copyedited.

Supporting Information

## References

[humu23046-bib-0001] 1000 Genomes Project Consortium , Auton A , Brooks LD , Durbin RM , Garrison EP , Kang HM , Korbel JO , Marchini JL , McCarthy S , McVean GA , Abecasis GR . 2015 A global reference for human genetic variation. Nature 526:68–74. 26432245 10.1038/nature15393 PMC4750478

[humu23046-bib-0002] Adzhubei IA , Schmidt S , Peshkin L , Ramensky VE , Gerasimova A , Bork P , Kondrashov AS , Sunyaev SR . 2010 A method and server for predicting damaging missense mutations. Nat Methods 7:248–249. 20354512 10.1038/nmeth0410-248 PMC2855889

[humu23046-bib-0003] Allali‐Hassani A , Wasney GA , Chau I , Hong BS , Senisterra G , Loppnau P , Shi Z , Moult J , Edwards AM , Arrowsmith CH , Park HW , Schapira M , et al. 2009 A survey of proteins encoded by non‐synonymous single nucleotide polymorphisms reveals a significant fraction with altered stability and activity. Biochem J 424:15–26. 19702579 10.1042/BJ20090723

[humu23046-bib-0004] Althage M , Bizouarn T , Rydström J . 2001 Identification of a region involved in the communication between the NADP(H) binding domain and the membrane domain in proton pumping *E. coli* transhydrogenase. Biochemistry (Mosc) 40:9968–9976. 10.1021/bi0103157 11502193

[humu23046-bib-0005] Andrikopoulos S . 2010 Obesity and type 2 diabetes: slow down!–can metabolic deceleration protect the islet beta cell from excess nutrient‐induced damage? Mol Cell Endocrinol 316:140–146. 19815054 10.1016/j.mce.2009.09.031

[humu23046-bib-0006] Arkblad EL , Egorov M , Shakhparonov M , Romanova L , Polzikov M , Rydström J . 2002 Expression of proton‐pumping nicotinamide nucleotide transhydrogenase in mouse, human brain and *C. elegans* . Comp Biochem Physiol B Biochem Mol Biol 133:13–21. 12223207 10.1016/s1096-4959(02)00107-0

[humu23046-bib-0007] Ashkenazy H , Erez E , Martz E , Pupko T , Ben‐Tal N . 2010 ConSurf 2010: calculating evolutionary conservation in sequence and structure of proteins and nucleic acids. Nucleic Acids Res 38:W529–533. 20478830 10.1093/nar/gkq399 PMC2896094

[humu23046-bib-0008] Bainbridge MN , Davis EE , Choi W‐Y , Dickson A , Martinez HR , Wang M , Dinh H , Muzny D , Pignatelli R , Katsanis N , Boerwinkle E , Gibbs R , et al. 2015 Loss of function mutations in NNT are associated with left ventricular noncompaction. Circ Cardiovasc Genet 8:544–552. 26025024 10.1161/CIRCGENETICS.115.001026 PMC4545476

[humu23046-bib-0009] Bergkvist A , Johansson C , Johansson T , Rydström J , Karlsson BG . 2000 Interactions of the NADP(H)‐binding domain III of proton‐translocating transhydrogenase from escherichia coli with NADP(H) and the NAD(H)‐binding domain I studied by NMR and site‐directed mutagenesis. Biochemistry (Mosc) 39:12595–12605. 10.1021/bi0004091 11027139

[humu23046-bib-0010] Bragg PD , Hou C . 2001 Characterization of mutants of beta histidine91, beta aspartate213, and beta asparagine222, possible components of the energy transduction pathway of the proton‐translocating pyridine nucleotide transhydrogenase of Escherichia coli. Arch Biochem Biophys 388:299–307. 11368169 10.1006/abbi.2001.2298

[humu23046-bib-0011] Brondijk THC , van Boxel GI , Mather OC , Quirk PG , White SA , Jackson JB . 2006 The role of invariant amino acid residues at the hydride transfer site of proton‐translocating transhydrogenase. J Biol Chem 281:13345–13354. 16533815 10.1074/jbc.M513230200

[humu23046-bib-0012] Chakrabarti P , Janin J . 2002 Dissecting protein‐protein recognition sites. Proteins 47:334–343. 11948787 10.1002/prot.10085

[humu23046-bib-0013] Circu ML , Aw TY . 2010 Reactive oxygen species, cellular redox systems, and apoptosis. Free Radic Biol Med 48:749–762. 20045723 10.1016/j.freeradbiomed.2009.12.022 PMC2823977

[humu23046-bib-0014] Consortium EA , Lek M , Karczewski K , Minikel E , Samocha K , Banks E , Fennell T , O'Donnell‐Luria A , Ware J , Hill A , Cummings B , Tukiainen T , et al. 2015 Analysis of protein‐coding genetic variation in 60,706 humans. bioRxiv 30338. 10.1038/nature19057 PMC5018207 27535533

[humu23046-bib-0015] Cotton NP , White SA , Peake SJ , McSweeney S , Jackson JB . 2001 The crystal structure of an asymmetric complex of the two nucleotide binding components of proton‐translocating transhydrogenase. Struct Lond Engl 9:165–176. 10.1016/s0969-2126(01)00571-8 11250201

[humu23046-bib-0016] Crooks GE , Hon G , Chandonia J‐M , Brenner SE . 2004 WebLogo: a sequence logo generator. Genome Res 14:1188–1190. 15173120 10.1101/gr.849004 PMC419797

[humu23046-bib-0017] Das J , Lee HR , Sagar A , Fragoza R , Liang J , Wei X , Wang X , Mort M , Stenson PD , Cooper DN , Yu H . 2014 Elucidating common structural features of human pathogenic variations using large‐scale atomic‐resolution protein networks. Hum Mutat 35:585–593. 24599843 10.1002/humu.22534 PMC4876038

[humu23046-bib-0018] David A , Razali R , Wass MN , Sternberg MJE . 2012 Protein‐protein interaction sites are hot spots for disease‐associated nonsynonymous SNPs. Hum Mutat 33:359–363. 22072597 10.1002/humu.21656

[humu23046-bib-0019] David A , Sternberg MJE . 2015 The Contribution of missense mutations in core and rim residues of protein‐protein interfaces to human disease. J Mol Biol 427:2886–2898. 26173036 10.1016/j.jmb.2015.07.004 PMC4548493

[humu23046-bib-0020] Dunker AK , Silman I , Uversky VN , Sussman JL . 2008 Function and structure of inherently disordered proteins. Curr Opin Struct Biol 18:756–764. 18952168 10.1016/j.sbi.2008.10.002

[humu23046-bib-0021] Emanuelsson O , Brunak S , von Heijne G , Nielsen H . 2007 Locating proteins in the cell using TargetP, SignalP and related tools. Nat Protoc 2:953–971. 17446895 10.1038/nprot.2007.131

[humu23046-bib-0022] Freeman H , Shimomura K , Horner E , Cox RD , Ashcroft FM . 2006 Nicotinamide nucleotide transhydrogenase: a key role in insulin secretion. Cell Metab 3:35–45. 16399503 10.1016/j.cmet.2005.10.008

[humu23046-bib-0023] Gregoret LM , Sauer RT . 1998 Tolerance of a protein helix to multiple alanine and valine substitutions. Fold Des 3:119–126. 9565756 10.1016/S1359-0278(98)00017-0

[humu23046-bib-0024] Habib SJ , Neupert W , Rapaport D . 2007 Analysis and prediction of mitochondrial targeting signals. Methods Cell Biol 80:761–781. 17445721 10.1016/S0091-679X(06)80035-X

[humu23046-bib-0025] Hanukoglu I . 2015 Proteopedia: Rossmann fold: A beta‐alpha‐beta fold at dinucleotide binding sites. Biochem Mol Biol Educ Bimon Publ Int Union Biochem Mol Biol 43:206–209. 10.1002/bmb.20849 25704928

[humu23046-bib-0026] Heiker JT , Kern M , Kosacka J , Flehmig G , Stumvoll M , Shang E , Lohmann T , Dreßler M , Kovacs P , Blüher M , Klöting N . 2013 Nicotinamide nucleotide transhydrogenase mRNA expression is related to human obesity. Obes Silver Spring Md 21:529–534. 10.1002/oby.20095 23592659

[humu23046-bib-0027] Hershkovitz E , Arafat M , Loewenthal N , Haim A , Parvari R . 2015 Combined adrenal failure and testicular adrenal rest tumor in a patient with nicotinamide nucleotide transhydrogenase deficiency. J Pediatr Endocrinol Metab 28:1187–1190. 25879317 10.1515/jpem-2015-0075

[humu23046-bib-0028] Holmberg E , Olausson T , Hultman T , Rydström J , Ahmad S , Glavas NA , Bragg PD . 1994 Prediction and site‐specific mutagenesis of residues in transmembrane alpha‐helices of proton‐pumping nicotinamide nucleotide transhydrogenases from *Escherichia coli* and bovine heart mitochondria. Biochemistry (Mosc) 33:7691–7700. 10.1021/bi00190a024 8011636

[humu23046-bib-0029] Jo S , Vargyas M , Vasko‐Szedlar J , Roux B , Im W . 2008 PBEQ‐Solver for online visualization of electrostatic potential of biomolecules. Nucleic Acids Res 36:W270‐275. 18508808 10.1093/nar/gkn314 PMC2447802

[humu23046-bib-0030] Johansson T , Oswald C , Pedersen A , Törnroth S , Okvist M , Karlsson BG , Rydström J , Krengel U . 2005 X‐ray structure of domain I of the proton‐pumping membrane protein transhydrogenase from *Escherichia coli* . J Mol Biol 352:299–312. 16083909 10.1016/j.jmb.2005.07.022

[humu23046-bib-0031] Karlsson J , Althage M , Rydström J . 2003 Roles of individual amino acids in helix 14 of the membrane domain of proton‐translocating transhydrogenase from Escherichia coli as deduced from cysteine mutagenesis. Biochemistry (Mosc) 42:6575–6581. 10.1021/bi034172v 12767241

[humu23046-bib-0032] Katoh K , Standley DM . 2013 MAFFT multiple sequence alignment software version 7: improvements in performance and usability. Mol Biol Evol 30:772–780. 23329690 10.1093/molbev/mst010 PMC3603318

[humu23046-bib-0033] Kelley LA , Mezulis S , Yates CM , Wass MN , Sternberg MJE . 2015 The Phyre2 web portal for protein modeling, prediction and analysis. Nat Protoc 10:845–858. 25950237 10.1038/nprot.2015.053 PMC5298202

[humu23046-bib-0034] Krengel U , Törnroth‐Horsefield S . 2015 Biochemistry. Coping with oxidative stress. Science 347:125–126. 25574006 10.1126/science.aaa3602

[humu23046-bib-0035] Kumar P , Henikoff S , Ng PC . 2009 Predicting the effects of coding non‐synonymous variants on protein function using the SIFT algorithm. Nat Protoc 4:1073–1081. 19561590 10.1038/nprot.2009.86

[humu23046-bib-0036] Kutzenko AS , Lamzin VS , Popov VO . 1998 Conserved supersecondary structural motif in NAD‐dependent dehydrogenases. FEBS Lett 423:105–109. 9506850 10.1016/s0014-5793(98)00074-x

[humu23046-bib-0037] Leung JH , Schurig‐Briccio LA , Yamaguchi M , Moeller A , Speir JA , Gennis RB , Stout CD . 2015 Structural biology. Division of labor in transhydrogenase by alternating proton translocation and hydride transfer. Science 347:178–181. 25574024 10.1126/science.1260451 PMC4479213

[humu23046-bib-0038] Meimaridou E , Kowalczyk J , Guasti L , Hughes CR , Wagner F , Frommolt P , Nürnberg P , Mann NP , Banerjee R , Saka HN , Chapple JP , King PJ , et al. 2012 Mutations in NNT encoding nicotinamide nucleotide transhydrogenase cause familial glucocorticoid deficiency. Nat Genet 44:740–742. 22634753 10.1038/ng.2299 PMC3386896

[humu23046-bib-0039] Meuller J , Mjörn K , Karlsson J , Tigerström A , Rydström J , Hou C , Bragg PD . 2001 Properties of a proton‐translocating nicotinamide nucleotide transhydrogenase from Escherichia coli with alpha and beta subunits linked through fused transmembrane helices. Biochim Biophys Acta 1506:163–171. 11779549 10.1016/s0005-2728(01)00191-8

[humu23046-bib-0040] Nishi H , Tyagi M , Teng S , Shoemaker BA , Hashimoto K , Alexov E , Wuchty S , Panchenko AR . 2013 Cancer missense mutations alter binding properties of proteins and their interaction networks. PloS One 8:e66273. 23799087 10.1371/journal.pone.0066273 PMC3682950

[humu23046-bib-0041] Novoselova TV , Rath SR , Carpenter K , Pachter N , Dickinson JE , Price G , Chan LF , Choong CS , Metherell LA . 2015 NNT pseudoexon activation as a novel mechanism for disease in two siblings with familial glucocorticoid deficiency. J Clin Endocrinol Metab 100:E350–354. 25459914 10.1210/jc.2014-3641 PMC4318891

[humu23046-bib-0042] Peake SJ , Jackson JB , White SA . 2000 The NADP(H)‐binding component (dIII) of human heart transhydrogenase: crystallization and preliminary crystallographic analysis. Acta Crystallogr D Biol Crystallogr 56:489–491. 10739929 10.1107/s0907444900001542

[humu23046-bib-0043] Pedersen A , Karlsson GB , Rydström J . 2008 Proton‐translocating transhydrogenase: an update of unsolved and controversial issues. J Bioenerg Biomembr 40:463–473. 18972197 10.1007/s10863-008-9170-x

[humu23046-bib-0044] Penn O , Privman E , Ashkenazy H , Landan G , Graur D , Pupko T . 2010 GUIDANCE: a web server for assessing alignment confidence scores. Nucleic Acids Res 38:W23‐28. 20497997 10.1093/nar/gkq443 PMC2896199

[humu23046-bib-0045] Petersen TN , Brunak S , von Heijne G , Nielsen H . 2011 SignalP 4.0: discriminating signal peptides from transmembrane regions. Nat Methods 8:785–786. 21959131 10.1038/nmeth.1701

[humu23046-bib-0046] Prasad GS , Sridhar V , Yamaguchi M , Hatefi Y , Stout CD . 1999 Crystal structure of transhydrogenase domain III at 1.2 A resolution. Nat Struct Biol 6:1126–1131. 10581554 10.1038/70067

[humu23046-bib-0047] Prasad GS , Wahlberg M , Sridhar V , Sundaresan V , Yamaguchi M , Hatefi Y , Stout CD . 2002 Crystal structures of transhydrogenase domain I with and without bound NADH. Biochemistry (Mosc) 41:12745–12754. 10.1021/bi020251f 12379117

[humu23046-bib-0048] Reuter S , Gupta SC , Chaturvedi MM , Aggarwal BB . 2010 Oxidative stress, inflammation, and cancer: how are they linked? Free Radic Biol Med 49:1603–1616. 20840865 10.1016/j.freeradbiomed.2010.09.006 PMC2990475

[humu23046-bib-0049] Ripoll VM , Meadows NA , Bangert M , Lee AW , Kadioglu A , Cox RD . 2012 Nicotinamide nucleotide transhydrogenase (NNT) acts as a novel modulator of macrophage inflammatory responses. FASEB J Off Publ Fed Am Soc Exp Biol 26:3550–3562. 10.1096/fj.11-199935 22593545

[humu23046-bib-0050] Robert X , Gouet P . 2014 Deciphering key features in protein structures with the new ENDscript server. Nucleic Acids Res 42:W320‐324. 24753421 10.1093/nar/gku316 PMC4086106

[humu23046-bib-0051] Rydström J . 2006 Mitochondrial NADPH, transhydrogenase and disease. Biochim Biophys Acta 1757:721–726. 16730324 10.1016/j.bbabio.2006.03.010

[humu23046-bib-0052] Sheeran FL , Rydström J , Shakhparonov MI , Pestov NB , Pepe S . 2010 Diminished NADPH transhydrogenase activity and mitochondrial redox regulation in human failing myocardium. Biochim Biophys Acta 1797:1138–1148. 20388492 10.1016/j.bbabio.2010.04.002

[humu23046-bib-0053] Small I , Peeters N , Legeai F , Lurin C . 2004 Predotar: A tool for rapidly screening proteomes for N‐terminal targeting sequences. Proteomics 4:1581–1590. 15174128 10.1002/pmic.200300776

[humu23046-bib-0054] Stefl S , Nishi H , Petukh M , Panchenko AR , Alexov E . 2013 Molecular mechanisms of disease‐causing missense mutations. J Mol Biol 425:3919–3936. 23871686 10.1016/j.jmb.2013.07.014 PMC3796015

[humu23046-bib-0055] UniProt Consortium . 2015 UniProt: a hub for protein information. Nucleic Acids Res 43:D204–212. 25348405 10.1093/nar/gku989 PMC4384041

[humu23046-bib-0056] Uttara B , Singh AV , Zamboni P , Mahajan RT . 2009 Oxidative stress and neurodegenerative diseases: a review of upstream and downstream antioxidant therapeutic options. Curr Neuropharmacol 7:65–74. 19721819 10.2174/157015909787602823 PMC2724665

[humu23046-bib-0057] Uversky VN , Oldfield CJ , Dunker AK . 2008 Intrinsically disordered proteins in human diseases: introducing the D2 concept. Annu Rev Biophys 37:215–246. 18573080 10.1146/annurev.biophys.37.032807.125924

[humu23046-bib-0058] Ward JJ , Sodhi JS , McGuffin LJ , Buxton BF , Jones DT . 2004 Prediction and functional analysis of native disorder in proteins from the three kingdoms of life. J Mol Biol 337:635–645. 15019783 10.1016/j.jmb.2004.02.002

[humu23046-bib-0059] Wass MN , Kelley LA , Sternberg MJE . 2010 3DLigandSite: predicting ligand‐binding sites using similar structures. Nucleic Acids Res 38:W469–473. 20513649 10.1093/nar/gkq406 PMC2896164

[humu23046-bib-0060] Weinberg‐Shukron A , Abu‐Libdeh A , Zhadeh F , Carmel L , Kogot‐Levin A , Kamal L , Kanaan M , Zeligson S , Renbaum P , Levy‐Lahad E , Zangen D . 2015 Combined mineralocorticoid and glucocorticoid deficiency is caused by a novel founder nicotinamide nucleotide transhydrogenase mutation that alters mitochondrial morphology and increases oxidative stress. J Med Genet 52:636–641. 26070314 10.1136/jmedgenet-2015-103078

[humu23046-bib-0061] White SA , Peake SJ , McSweeney S , Leonard G , Cotton NP , Jackson JB . 2000 The high‐resolution structure of the NADP(H)‐binding component (dIII) of proton‐translocating transhydrogenase from human heart mitochondria. Struct Lond Engl 8:1–12. 10.1016/s0969-2126(00)00075-7 10673423

[humu23046-bib-0062] Worth CL , Blundell TL . 2010 On the evolutionary conservation of hydrogen bonds made by buried polar amino acids: the hidden joists, braces and trusses of protein architecture. BMC Evol Biol 10:161. 20513243 10.1186/1471-2148-10-161 PMC2892493

[humu23046-bib-0063] Yamaguchi M , Hatefi Y . 1995 Proton‐translocating nicotinamide nucleotide transhydrogenase of Escherichia coli. Involvement of aspartate 213 in the membrane‐intercalating domain of the beta subunit in energy transduction. J Biol Chem 270:16653–16659. 7622474 10.1074/jbc.270.28.16653

[humu23046-bib-0064] Yamaguchi M , Stout CD . 2003 Essential glycine in the proton channel of Escherichia coli transhydrogenase. J Biol Chem 278:45333–45339. 12952962 10.1074/jbc.M308236200

[humu23046-bib-0065] Yamaguchi R , Kato F , Hasegawa T , Katsumata N , Fukami M , Matsui T , Nagasaki K , Ogata T . 2013 A novel homozygous mutation of the nicotinamide nucleotide transhydrogenase gene in a Japanese patient with familial glucocorticoid deficiency. Endocr J 60:855–859. 23474776 10.1507/endocrj.ej13-0024

[humu23046-bib-0066] Yates CM , Filippis I , Kelley LA , Sternberg MJE . 2014 SuSPect: enhanced prediction of single amino acid variant (SAV) phenotype using network features. J Mol Biol 426:2692–2701. 24810707 10.1016/j.jmb.2014.04.026 PMC4087249

[humu23046-bib-0067] Yates CM , Sternberg MJE . 2013 The effects of non‐synonymous single nucleotide polymorphisms (nsSNPs) on protein‐protein interactions. J Mol Biol 425:3949–3963. 23867278 10.1016/j.jmb.2013.07.012

[humu23046-bib-0068] Yue P , Moult J . 2006 Identification and analysis of deleterious human SNPs. J Mol Biol 356:1263–1274. 16412461 10.1016/j.jmb.2005.12.025

