# Supplementary material for: Three‐Dimensional Model of Human Nicotinamide Nucleotide Transhydrogenase (NNT) and Sequence‐Structure Analysis of its Disease‐Causing Variations
Source: Hum Mutat. 2016 Aug 8;37(10):1074–84. doi: 10.1002/humu.23046 (PMC5026163; doi:10.1002/humu.23046)
Supplement: Supplementary file 1 — Supporting Information [file HUMU-37-1074-s001.pdf]

SUPPORTING INFORMATION FOR THE ARTICLE

Three-dimensional model of human Nicotinamide Nucleotide Transhydrogenase (NNT) and sequence-structure analysis of its disease-causing variations

Louise A Metherell [1], José Afonso Guerra-Assunção [2], Michael J Sternberg [3], Alessia David [3]

Supp. Methods

Phyre2 uses template-based modelling (TBM, or homology modelling) to generate a structural model. TBM aligns the target amino acid sequence to the sequence of known structures deposited in the ProteinDataBank (PDB), on the basis of patterns of evolutionary variation and is the most reliable and frequently used technique for structural modelling (Daga et al. 2010) (Kelley et al. 2015). Phyre2 uses the alignment of Hidden Markov models via HHsearch (Söding 2005) to significantly improve the accuracy of alignment and detection rate. A match with a confidence score >90% indicates that the query protein adopts the overall predicted fold and that the core of the protein is modeled at high accuracy (2–4 Å r.m.s.d. from the native, true structure). However, surface loops do probably deviate from the native (Kelley et al. 2015). Regions not covered by templates are modelled *ab initio* using the Poing algorithm embedded in Phyre2. H-NNT domain I and II homodimeric structures were generated manually by using *E. coli* domain I dimer and *Tt*-NNT domain II dimer as templates. The holo H-NNT dimer model was generated manually by superposing individual H-NNT domains to the holo *Tt*-NNT (PDB 4O9U) crystallized by Leung et al. (Leung et al. 2015).

To ensure model reliability, we required a sequence identity > 30% between NNT and its structural templates. This sequence identity cut-off was chosen because it is widely accepted as the minimum required for successful homology modeling (Xiang 2006) (Kelley et al. 2015). The quality of individual models was checked using the following software: ProSA, (Wiederstein and Sippl 2007), which calculates an overall quality score for the query protein model. A quality score that falls

outside the range of scores typically found for native proteins of similar size, indicates that the model structure probably contains errors; PROCHECK (Laskowski et al. 1996), which evaluates the stereochemical properties of protein models.

NAD was modelled with H-NNT domain I using 3DligadSite (Wass et al. 2010)

Supp. Figure S1 Multiple sequence alignment.

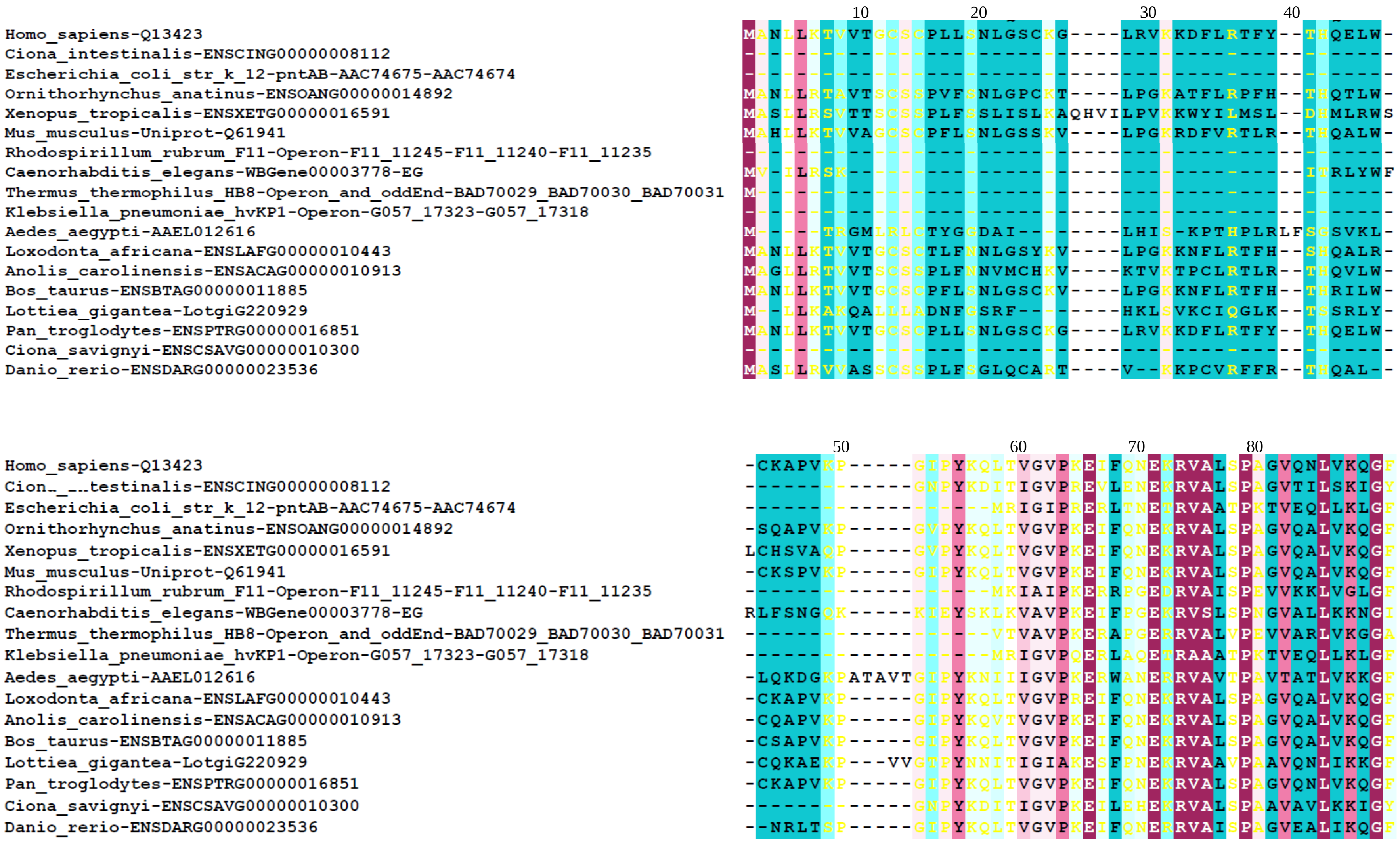

```
                                                              90        100       110       120       130
Homo_sapiens-Q13423                                           NVVVESGAGEASKFSDDHYRVAGAQIQG--AKEVLA-SDLVVKVRAPMVN
Ciona_intestinalis-ENSCING00000008112                         KVKVEENAGEASEFSNAMYSEVGATIAS--KKDVYS-SDILLKVRAPELT
Escherichia_coli_str_k_12-pntAB-AAC74675-AAC74674             TVAVESGAGQLASFDDKAFVQAGAEIVE--GNSVWQ-SEIILKVNAPLDD
Ornithorhynchus_anatinus-ENSOANG00000014892                   NVVVESGAGEASKFSDDHYRAAGAQIQG--TKEVLA-SDVVVKVRAPMVN
Xenopus_tropicalis-ENSXETG00000016591                         NVVVEAGAGEASKFSDDQYKEAGAKIQG--TKDVLA-SDLVLKVRAPMLN
Mus_musculus-Uniprot-Q61941                                   NVVVESGAGEASKFPDDLYRAAGAQIQG--MKEVLA-SDLVVKVRAPMVN
Rhodospirillum_rubrum_F11-Operon-F11_11245-F11_11240-F11_11235 EVIVEQGAGVGASITDDALTAAGATIASTAAQALSQ-ADVVWKVQRPMTA
Caenorhabditis_elegans-WBGene00003778-EG                      SVLIEENAGVLAGYSNEEYVRSGADVGK--HNEVFN-TDIMLKVRPPTEN
Thermus_thermophilus_HB8-Operon_and_oddEnd-BAD70029_BAD70030_BAD70031 RVRVERGAGEGAYHPDEAYQEAGAEVVE--RGELLKGAHLLFTVQPPPED
Klebsiella_pneumoniae_hvKP1-Operon-G057_17323-G057_17318      SVAVESGAGKLASFDDEAFAEAGAEIVT--GDEVWQ-SDVILKVNAPNDD
Aedes_aegypti-AAEL012616                                      KVNVESGAGFDAKFRDADYASAGANIVD--KSQAFQ-SDIVLKVRQPMDN
Loxodonta_africana-ENSLAFG00000010443                         NVVVESGAGEASKFSDDHYRAAGAQIQG--TKEVLA-SDLVVKVRAPMVN
Anolis_carolinensis-ENSACAG00000010913                        NVIVESNAGEASKFSDDHYKEAGAQIQG--TEEVLA-SDLVVKVRAPMLN
Bos_taurus-ENSBTAG00000011885                                 NVVVESGAGEASKFSDDHYRAAGAQIQG--AKEVLA-SDLVVKVRAPMLN
Lottiea_gigantea-LotgiG220929                                 NVNVEENAGVEAKFKNDDYAAAGANIKS--TKDLFH-SDIVLKVRGPSME
Pan_troglodytes-ENSPTRG00000016851                            NVVVESGAGEASKFSDDHYRAAGAQIQG--AKEVLA-SDLVVKVRAPMVN
Ciona_savignyi-ENSCSAVG00000010300                            NVNIEKSAGDKSEFSDAMYTELGASIGS--KSDVFA-SDILLKVRAPELS
Danio_rerio-ENSDARG00000023536                                NVVVESGAGESAKFSDDMYTKAGATIRD--VKDVFS-SDVLLKVRAPMLN
```

```
                                                                 140       150       160       170       180
Homo_sapiens-Q13423                                           PTLGVHEADLLKTSGTLISFIYPAQNPELLNKLSQRKTTVLAMDQVPRVT
Ciona_intestinalis-ENSCING00000008112                         ------EIQQMKPNSTLFSYLYPAQNPQLLDALAENKMTAFAMDQVPRVT
Escherichia_coli_str_k_12-pntAB-AAC74675-AAC74674             ------EIALLNPGTTLVSFIWPAQNPELMQKLAERNVTVMAMDSVPRIS
Ornithorhynchus_anatinus-ENSOANG00000014892                   PALGVHEADLFKPSGTLISFIYPAQNPDLLDKLAGRKSTVLAMDQVPRVT
Xenopus_tropicalis-ENSXETG00000016591                         PALGVHEADLFKSSSTLISFIYPAQNPDLLSKLSEKNMTILAMDQVPRVT
Mus_musculus-Uniprot-Q61941                                   PTLGAHEADFLKPSGTLISFIYPAQNPDLLNKLSERKTTVLAMDQVPRVT
Rhodospirillum_rubrum_F11-Operon-F11_11245-F11_11240-F11_11235 -EEGTDEVALIKEGAVLMCHLGALTNRPVVEALTKRKITAYAMELMPRIS
Caenorhabditis_elegans-WBGene00003778-EG                      ------EVSKLKSGCTLISFIHPGQNQALLDSLTKTDKTVFAMDCVPRIS
Thermus_thermophilus_HB8-Operon_and_oddEnd-BAD70029_BAD70030_BAD70031 ------LIQALEPGAIVVGFVQPHKNLELVRALQAKKATVIAMELIPRIT
Klebsiella_pneumoniae_hvKP1-Operon-G057_17323-G057_17318      ------EIALLNPGTTLISFIWPAQNPQLMEKLAARNINVMAMDSVPRIS
Aedes_aegypti-AAEL012616                                      ------EIPTLKENSTLISFLYPTQNKDLIDKLSQKKLNAFAMDAIPRIS
Loxodonta_africana-ENSLAFG00000010443                         PTLGVHEADLLKTSGTLISFIYPAQNPDLLSKLSERKTTVLAMDQVPRVT
Anolis_carolinensis-ENSACAG00000010913                        PALGVHEADLFKSPSTLISFIYPAQNPDLLNKLSERKTTVLAMDQVPRVT
Bos_taurus-ENSBTAG00000011885                                 PTLGVHEADLLKTSGTLISFIYPAQNPDLLNKLSKRKTTVLAMDQVPRVT
Lottiea_gigantea-LotgiG220929                                 ------EVSQFKDGGTLISFLYPAQNKDLVDKLGERRMTAFAMDCVPRIS
Pan_troglodytes-ENSPTRG00000016851                            PTLGVHEADLLKTSGTLISFIYPAQNPELLNKLSQRKTTVLAMDQVPRVT
Ciona_savignyi-ENSCSAVG00000010300                            ------EIDQLKPNSTLFSYIYPTQNPDLLEKLAQKNMTVFAMDQVPRVT
Danio_rerio-ENSDARG00000023536                                PTLGVHEASLMSEGATLVSFIYPAQNPELMDTLSQRKATVLAMDQVPRVT
```

```
                                                                    190       200       210       220       230
Homo_sapiens-Q13423                                                 IAQGYDALSSMANIAGYKAVVLAANHFGRFFTGQITAAGKVPPAKILIVG
Ciona_intestinalis-ENSCING00000008112                               IAQAFDALSSMSNISGYKAIVLAANNFGRFFTGQITAAGKVPPAKVLIIG
Escherichia_coli_str_k_12-pntAB-AAC74675-AAC74674                   RAQSLDALSSMANIAGYRAIVEAAHEFGRFFTGQITAAGKVPPAKVMVIG
Ornithorhynchus_anatinus-ENSOANG00000014892                         IAQGYDALSSMANIAGYKAVVLAANHFGRFFTGQITAAGKVPPAKILIIG
Xenopus_tropicalis-ENSXETG00000016591                               IAQGYDALSSMANIAGYKAVVMAANNFGRFFTGQITAAGKVPPAKVLIIG
Mus_musculus-Uniprot-Q61941                                         IAQGYDALSSMANISGYKAVVLAANHFGRFFTGQITAAGKVPPAKILIVG
Rhodospirillum_rubrum_F11-Operon-F11_11245-F11_11240-F11_11235      RAQSMDILSSQSNLAGYRAVIDGAYEFARAFPMMMTAAGTVPPARVLVFG
Caenorhabditis_elegans-WBGene00003778-EG                            RAQVFDALSSMANIAGYRAVIEAANHFGRFFTGQITAAGKVPPAKVLVIG
Thermus_thermophilus_HB8-Operon_and_oddEnd-BAD70029_BAD70030_BAD70031 RAQSMDALSSQATVAGYLAAIHAARLSPRFFPMLTTAAGTIRPAKVMVMG
Klebsiella_pneumoniae_hvKP1-Operon-G057_17323-G057_17318            RAQSLDALSSMANIAGYRAIVEAAHEFGRFFTGQITAAGKVPPAKVMVIG
Aedes_aegypti-AAEL012616                                            RAQVFDALSSMANISGYRAVIEAANHFPRFFTGQITAAGKVPPAKILVIG
Loxodonta_africana-ENSLAFG00000010443                               IAQGYDALSSMANIAGYKAVVLAANHFGRFFTGQITAAGKVPPAKILIVG
Anolis_carolinensis-ENSACAG00000010913                              IAQGYDALSSMANIAGYKAVVLAANHFGRFFTGQITAAGKVPPAKVLIIG
Bos_taurus-ENSBTAG00000011885                                       IAQGYDALSSMANIAGYKAVVLAANHFGRFFTGQITAAGKVPPAKILIVG
Lottiea_gigantea-LotgiG220929                                       RAQVFDALSSMANIAGYKAVLEAANNFPRFFAGQITAAGKVPPAKVLVIG
Pan_troglodytes-ENSPTRG00000016851                                  IAQGYDALSSMANIAGYKAVVLAANHFGRFFTGQITAAGKVPPAKILIVG
Ciona_savignyi-ENSCSAVG00000010300                                  IAQAFDVLSSMSNISGYKAIVLAANHFGRFFTGQITAAGKVPPAKVLIIG
Danio_rerio-ENSDARG00000023536                                      IAQGYDALSSMANIAGYKAVVLAANNFGRFFTGQITAAGKVPPAKVLIIG
```

```
                                                                    240       250       260       270       280
Homo_sapiens-Q13423                                                 GGVAGLASAGAAKSMGAIVRGFDTRAAALEQFKSLGAEPLEVDLK--ESG
Ciona_intestinalis-ENSCING00000008112                               GGVAGLASAGTAKGMGAIVRGFDTRDAALEQFKSLGAEPLKVDIE--ESG
Escherichia_coli_str_k_12-pntAB-AAC74675-AAC74674                   AGVAGLAAIGAANSLGAIVRAFDTRPEVKEQVQSMGAEFLELDFK--EEA
Ornithorhynchus_anatinus-ENSOANG00000014892                         GGVAGLAAAGAAKSMGAVVRGFDTRAAALEQFKSLGAEPLEVDLK--ESG
Xenopus_tropicalis-ENSXETG00000016591                               GGVAGLAAAGAAKSMGAIVRGFDTRAAALEQFKSLGAEPLEVDLK--ESG
Mus_musculus-Uniprot-Q61941                                         GGVAGLASAGAAKSMGAVVRGFDTRAAALEQFKSLGAEPLEVDLK--ESG
Rhodospirillum_rubrum_F11-Operon-F11_11245-F11_11240-F11_11235      VGVAGLQAIATAKRLGAVVMATDVRAATKEQVESLGGKFITVDDEAMKTA
Caenorhabditis_elegans-WBGene00003778-EG                            GGVAGLSAIGTSRGMGAVVRGFDTRAAVKEHVESLGAQFLTVNVK--EDG
Thermus_thermophilus_HB8-Operon_and_oddEnd-BAD70029_BAD70030_BAD70031 VGVAGLMAIATAKRLGAQVFAYDVRKAALEQALSLGAKPIELPIS----A
Klebsiella_pneumoniae_hvKP1-Operon-G057_17323-G057_17318            AGVAGLAAIGAANSLGAIVRAFDTRPEVKEQVQSMGAEFLELDFK--EEA
Aedes_aegypti-AAEL012616                                            GGVAGLAAIGQARGMGAIVRAFDTRPVVKEQVESMGAEFLTINIE--EDG
Loxodonta_africana-ENSLAFG00000010443                               GGVAGLASAGAAKSMGAIVRGFDTRAAALEQFKSLGAEPLEVDLK--ESG
Anolis_carolinensis-ENSACAG00000010913                              GGVAGLAAAGAAKSMGAVVRGFDTRAEALEQFKSLGAEPLEVDLK--ESG
Bos_taurus-ENSBTAG00000011885                                       GGVAGLASAGAAKSMGAIVRGFDTRAAALEQFKSLGAEPLEVDLK--ESG
Lottiea_gigantea-LotgiG220929                                       GGVAGLSAIGTAKNLGAIVRGFDTRAAVKEQVESFGAEFLEVNIK--ESG
Pan_troglodytes-ENSPTRG00000016851                                  GGVAGLASAGAAKSMGAIVRGFDTRAAALEQFKSLGAEPLEVDLK--ESG
Ciona_savignyi-ENSCSAVG00000010300                                  GGVAGLASAGTAKGMGAIVRGFDTRDAALEQFKSLGAEPLKVDIE--ESG
Danio_rerio-ENSDARG00000023536                                      GGVAGLAAAGSARAMGAIVRGFDTRAAALEQFKSLGAEPLEVDIK--ESG
```

```
                                                                            290       300       310       320         330
Homo_sapiens-Q13423                                                EGQGGYAKEMSKEFIEAEMKLFAQQCKEVDILISTALIP--GKKAPVLFN
Ciona_intestinalis-ENSCING00000008112                              EGGGGYAKEMSKEFIEAEMALFAKQAKEVDIIVTTALIP--GKPAPKLIT
Escherichia_coli_str_k_12-pntAB-AAC74675-AAC74674                  GSGDGYAKVMSDAFIKAEMELFAAQAKEVDIIVTTALIP--GKPAPKLIT
Ornithorhynchus_anatinus-ENSOANG00000014892                        EGQGGYAKEMSKEFIEAEMQLFAKQCKEVDILISTALIP--GKKAPVLFR
Xenopus_tropicalis-ENSXETG00000016591                              EGQGGYAKEMSKEFIDAEMKLFAKQCQDVDIIVTTALIPETGKKAPTLFR
Mus_musculus-Uniprot-Q61941                                        EGQGGYAKEMSKEFIEAEMKLFAQQCKEVDILISTALIP--GKKAPVLFS
Rhodospirillum_rubrum_F11-Operon-F11_11245-F11_11240-F11_11235     ETAGGYAKEMGEEFRKKQAEAVLKELVKTDIAITTALIP--GKPAPVLIT
Caenorhabditis_elegans-WBGene00003778-EG                           EGGGGYAKEMSKEFIDAEMKLFADQCKDVDIIITTALIP--GKKAPILIT
Thermus_thermophilus_HB8-Operon_and_oddEnd-BAD70029_BAD70030_BAD70031 EGEGGYARELTEEEKRIQHEALRDHVAGMDVLITTAQVP--GRRAPILLT
Klebsiella_pneumoniae_hvKP1-Operon-G057_17323-G057_17318           GSGDGYAKVMSEAFIKAEMALFAAQAKEVDIIVTTALIP--GKPAPKLIT
Aedes_aegypti-AAEL012616                                           STAGGYSKEMSKEFIEAEMSLFAKQCKEVDVIITTALIP--GRKAPILIT
Loxodonta_africana-ENSLAFG00000010443                              EGQGGYAKEMSKEFIEAEMKLFAQQCKEVDILISTALIP--GKKAPVLFR
Anolis_carolinensis-ENSACAG00000010913                             EGQGGYAKEMSKEFIEAEMKLFAKQCQDVDIIITTALIP--GKKAPILFR
Bos_taurus-ENSBTAG00000011885                                      EGQGGYAKEMSKEFIEAEMKLFAQQCKEVDILISTALIP--GKKAPILFN
Lottiea_gigantea-LotgiG220929                                      EGTGGYAKTMSKEFIEAEMALFAKQCKEIDILITTALIP--GKPAPKLIS
Pan_troglodytes-ENSPTRG00000016851                                 EGQGGYAKEMSKEFIEAEMKLFAQQCKEVDILISTALIP--GKKAPVLFN
Ciona_savignyi-ENSCSAVG00000010300                                 DGAGGYAKEMSKEFIEAEMALFAKQCKEVDIVVTTALIP--GKPAPKLIT
Danio_rerio-ENSDARG00000023536                                     EGQGGYAKEMSKEFIEAEMKLFAKQCLDVDIIITTALIP--GRKAPVLIT
```

```
                                                                        340       350       360       370
Homo_sapiens-Q13423                                                KEMIESMKEGSVVVDLAAEAGGNFETTKPGELY-IHKGITHIGYTDLPSR
Ciona_intestinalis-ENSCING00000008112                              KEMVESMKDGSVIVDLASEAGGNCALTKPGKLY-KHGGVTIIGYTDLPSR
Escherichia_coli_str_k_12-pntAB-AAC74675-AAC74674                  REMVDSMKAGSVIVDLAAQNGGNCEYTVPGEIFTTENGVKVIGYTDLPGR
Ornithorhynchus_anatinus-ENSOANG00000014892                        KDMIESMKEGSVVVDLAAEAGGNFETTKPGELY-VHKGVTHIGYTDLPSR
Xenopus_tropicalis-ENSXETG00000016591                              KDMIELMKEGSVVVDLAAEAGGNIETTKPGDLY-VHKKVIHVLYTEVMCY
Mus_musculus-Uniprot-Q61941                                        KEMIESMKEGSVVVDLAAEAGGNFETTKPGELY-VHKGITHIGYTDLPSR
Rhodospirillum_rubrum_F11-Operon-F11_11245-F11_11240-F11_11235     EEMVTKMKPGSVIIDLAVEAGGNCPLSEPGKIV-VKHGVKIVGHTNVPSR
Caenorhabditis_elegans-WBGene00003778-EG                           EEMIKSMKPGSVVVDLAAESGGNIATTRPGEVY-VKHGVTHIGFTDLPSR
Thermus_thermophilus_HB8-Operon_and_oddEnd-BAD70029_BAD70030_BAD70031 EDMVERLKPGTVVVDLAAESGGNCVLTKPGEVV-EVRGVRVYGPLNLPSE
Klebsiella_pneumoniae_hvKP1-Operon-G057_17323-G057_17318           REMVDSMKSGSVVVDLASQNGGNCEYTVPGEVVTTANGVKIIGYTDLPGR
Aedes_aegypti-AAEL012616                                           EEMVKSMKPGSVIVDLAAEAGGNVATTVPGKVE-VVHDVVHVGLTDFPSR
Loxodonta_africana-ENSLAFG00000010443                              KEMIESMKEGSVVVDLAAEAGGNFETTKPGELY-VHKGVTHIGYTDLPSR
Anolis_carolinensis-ENSACAG00000010913                             KDMIELMKEGSVVVDLAAEAGGNIETTKPGELY-IHKGITHIGYTDLPSR
Bos_taurus-ENSBTAG00000011885                                      KEMIESMKEGSVVVDLAAEAGGNFETTKPGELY-VHKGITHIGYTDLPSR
Lottiea_gigantea-LotgiG220929                                      KQMIESMKPGSVVVDLASEAGGNIETIKPGELY-RYNDVVHIGYTDFPSR
Pan_troglodytes-ENSPTRG00000016851                                 KEMIESMKEGSVVVDLAAEAGGNFETTKPGELY-IHKGITHIGYTDLPSR
Ciona_savignyi-ENSCSAVG00000010300                                 KQMVESMKDGSVIVDLAAEAGGNCELTKPGELY-KHGGVTIIGYTDIPSR
Danio_rerio-ENSDARG00000023536                                     KEMVETMKDGSVVVDLAAEAGGNIETTVPGELS-VHKGVIHVGYTDIPSR
```

```
                                                                       380       390       400       410       420
Homo_sapiens-Q13423                                                    MATQASTLYSNNITKLLKAISPDKD--NFYFDVKDDFDFGTMGHVIRGTV
Ciona_intestinalis-ENSCING00000008112                                  LPTQSSTLYSNNMVKLIKAISPDKE--VYDFEFKDDFAYGNVDHVVRGSC
Escherichia_coli_str_k_12-pntAB-AAC74675-AAC74674                      LPTQSSQLYGTNLVNLLKLLCKEKD-GNITVDFDDV--------VIRGVT
Ornithorhynchus_anatinus-ENSOANG00000014892                            MATQASTLYSNNITKLLKAISPDKD--NFYLDVKDEFDFGTMGHVVRGTV
Xenopus_tropicalis-ENSXETG00000016591                                  VTCHSLFLYENKETRIIKNCTSNTS--TAMKQIKTTLIIQSPYNIQCKCK
Mus_musculus-Uniprot-Q61941                                            MATQASTLYSNNITKLLKAISPDKD--NFHFEVKDDFDFGTMSHVIRGTV
Rhodospirillum_rubrum_F11-Operon-F11_11245-F11_11240-F11_11235         VAADASPLFAKNLLNFLTPHV-DKDTKTLVMKLEDE--------TVSGTC
Caenorhabditis_elegans-WBGene00003778-EG                               LPTQSSELYSNNIAKFLLHLGKDK---TFFVNEEDE--------VARGAL
Thermus_thermophilus_HB8-Operon_and_oddEnd-BAD70029_BAD70030_BAD70031  LSVHASEMYAKNLYNLSSLLI-EKG--AFAPKWEDE--------IVRAAL
Klebsiella_pneumoniae_hvKP1-Operon-G057_17323-G057_17318               LPTQSSQLYGTNLVNLLKLLCKEKD-GNIVIDFDDV--------VVRGVT
Aedes_aegypti-AAEL012616                                               LPTQSSTLYANNISKFLLSMG-EKD--HFNINLEDE--------VVRGSI
Loxodonta_africana-ENSLAFG00000010443                                  MATQASTLYSNNITKLLKAISPDKD--HFYFEVKDDFDFGTMGHVIRGTV
Anolis_carolinensis-ENSACAG00000010913                                 MATQASSLYSNNITKLLKAISPDKD--NFYFNLKDDFDYGTMDHVVRGTV
Bos_taurus-ENSBTAG00000011885                                          MATQASTLYSNNITKLLKAISPDKD--NFYFEVKDDFDFGTMGHVIRGTV
Lottiea_gigantea-LotgiG220929                                          LPTQSSTLYGNNISKFLLSIG-EKE--HYNVNLEDE--------VVRGSI
Pan_troglodytes-ENSPTRG00000016851                                     MATQASTLYSNNITKLLKAISPDKD--NFYFDVKDDFDFGTMGHVIRGTV
Ciona_savignyi-ENSCSAVG00000010300                                     LPTQSSNLYANNMVKLIKAISPDKE--TFNFEFKDDFSYGNIDHVVRGSC
Danio_rerio-ENSDARG00000023536                                         LPTQASTLYSNNITKLIRAISPDKE--TFYFDVKNEFDFGTMDHVIRGSV
```

```
                                                                       430       440       450       460       470
Homo_sapiens-Q13423                                                    VMKDGKVIFPAPTPKNIPQGAPVKQKTVA---ELEAEKAATITPFRKTMS
Ciona_intestinalis-ENSCING00000008112                                  VVHEGKITFPSPAPQNVPQPTP-PPKPVE---TQEV--AEPESAFASKFK
Escherichia_coli_str_k_12-pntAB-AAC74675-AAC74674                      VIRAGEITWPAPPIQVSAQPQA-AQKAAP---EVKTEEKCTCSPWRKYAL
Ornithorhynchus_anatinus-ENSOANG00000014892                            VMKDGKVIFPAPVPKNIPQGAPVKQKSVA---ELEAEKAATITPFQKTMT
Xenopus_tropicalis-ENSXETG00000016591                                  VQKDGKVIFPAPPPNNIPQGTPVKQKSVA---ELEAEKAASISPFRKTFN
Mus_musculus-Uniprot-Q61941                                            VMKDGKVIFPAPTPKNIPEEAPVKPKTVA---ELEAEKAGTVSMYTKTLT
Rhodospirillum_rubrum_F11-Operon-F11_11245-F11_11240-F11_11235         VTRDGAIVHPALTGQGAXXXMEDKNILVEGFNQLSQQALELSQHAQALAL
Caenorhabditis_elegans-WBGene00003778-EG                               VVRDGQMKWPPPPINFPPPAAP---KSDK---PSENTALVPLTPFRKTAN
Thermus_thermophilus_HB8-Operon_and_oddEnd-BAD70029_BAD70030_BAD70031  LMKEGEVLHGP---------------------------------------
Klebsiella_pneumoniae_hvKP1-Operon-G057_17323-G057_17318               VVREGEITWPAPPIQVSAQPQA-AAKKV----EAPKEAVKPASPWRKYAL
Aedes_aegypti-AAEL012616                                               VLQNGNLMWPPPVISVSAKPPPAVAAAAT---PATKVEVQPPNPFNDTLK
Loxodonta_africana-ENSLAFG00000010443                                  VMKDGKVIFPAPTPKNIPQGAPVKQKTVA---ELEAEKAATITPYRKTVT
Anolis_carolinensis-ENSACAG00000010913                                 VMKDGKVIFPAPLPKKIPQAAPVKQKTVA---EIEAEKAATITPFRKTMT
Bos_taurus-ENSBTAG00000011885                                          VMKDGQVIFPAPTPKNIPQGAPVKQKTVA---ELEAEKAATITPFRKTMT
Lottiea_gigantea-LotgiG220929                                          ILHEGKLMWPPPAPKVDPAAPP--PAPAA---KVEKVAPPPPNYFNMTMK
Pan_troglodytes-ENSPTRG00000016851                                     VMKDGKVIFPAPTPKNIPQGAPVKQKTVA---ELEAEKAATITPFRKTMS
Ciona_savignyi-ENSCSAVG00000010300                                     VVHEGKVTFPSPAPQNIPQPTPPPPKPTE---KLEV--AEPESAFASKFK
Danio_rerio-ENSDARG00000023536                                         VMQDGKVLFPAPQPQNVPVAAPPKQKTVQ---ELQKEKASAVSPFRATLT
```

```
                                                                                 480       490       500       510       520
Homo_sapiens-Q13423                                                         TASAY-TAGLTGILGLGIAAPNLAFSQMVTTFGLAGIVGYHTVWGVTPAL
Ciona_intestinalis-ENSCING00000008112                                       ESAIYTTGGLTGLVGMGVLSPTPSVTQMVTTCGISIIVGYHTVWGVAPAL
Escherichia_coli_str_k_12-pntAB-AAC74675-AAC74674                           MALAI------ILFGWMASVAPKEFLGHFTVFALACVVGYYVVWNVSHAL
Ornithorhynchus_anatinus-ENSOANG00000014892                                 TASVY-TTGVAGLLGLGIVAPTSAFSQMVTTFGLAGIVGYHTVWGVTPAL
Xenopus_tropicalis-ENSXETG00000016591                                       GAAAY-TAGLGTLLSLGIASPHSAFTQMVTTFGLAGIVGYHTVWGVTPAL
Mus_musculus-Uniprot-Q61941                                                 TASVY-SAGLTGMLGLGIVAPNVAFSQMVTTFGLAGIIGYHTVWGVTPAL
Rhodospirillum_rubrum_F11-Operon-F11_11245-F11_11240-F11_11235              QASHA------VLPAAAATEGASEFWWLMTVFVLACFIGFYVVWSVTPAL
Caenorhabditis_elegans-WBGene00003778-EG                                    QTLLL-TSGLGSVSLLGIAGTNPQISSMSTTFALAGLVGYHTVWGVTPAL
Thermus_thermophilus_HB8-Operon_and_oddEnd-BAD70029_BAD70030_BAD70031       ---------TKALLGGAXXXMEFNFWSALYIFVLTAFLGYELITRVPVIL
Klebsiella_pneumoniae_hvKP1-Operon-G057_17323-G057_17318                    MALAI------ILFGWLANVAPKEFLGHFTVFALACVVGYYVVWNVSHAL
Aedes_aegypti-AAEL012616                                                    NSMLY-TTGLGSLLGLGAISPNPAFTTMMTTFAMSGIVGYHTVWGVTPAL
Loxodonta_africana-ENSLAFG00000010443                                       SASAY-TAGLTSILGLGIAAPNLAFSQMVTTFGLAGIVGYHTVWGVTPAL
Anolis_carolinensis-ENSACAG00000010913                                      TASAY-TAGLASMLGLGLAAPNSAFTQMVTTFGLAGIVGYHTVWGVTPAL
Bos_taurus-ENSBTAG00000011885                                               SASVY-TAGLTGILGLGIAAPNLAFSQMVTTFGLAGIVGYHTVWGVTPAL
Lottiea_gigantea-LotgiG220929                                               SAGKY-SAGFGTIVGCGMISPNPQFTTMVTTFGLSGIVGYYTVWGVTPAL
Pan_troglodytes-ENSPTRG00000016851                                          TASAY-TAGLTGILGLGIAAPNLAFSQMVTTFGLAGIVGYHTVWGVTPAL
Ciona_savignyi-ENSCSAVG00000010300                                          ETALYTTGGLTGLVGMGVLSPTPSVTQMITTCGVSIIVGYHTVWGVAPAL
Danio_rerio-ENSDARG00000023536                                              TAGVY-TGGLGTAIGLGLCAPNAAFTQMVTTFGLAGIVGYHTVWGVTPAL
```

```
                                                                                 530       540       550       560       570
Homo_sapiens-Q13423                                                         HSPLMSVTNAISGLTAVGGLALMGGHLYPSTTSQGLAALAAFISSVNIAG
Ciona_intestinalis-ENSCING00000008112                                       HSPLMSVTNAISGLTAAGGLFLMGGEYLPNTTAQSLALASTFISSINIFG
Escherichia_coli_str_k_12-pntAB-AAC74675-AAC74674                           HTPLMSVTNAISGIIVVGALLQIGQG----GWVSFLSFIAVLIASINIFG
Ornithorhynchus_anatinus-ENSOANG00000014892                                 HSPLMSVTNAISGLTAVGGLALMGGNYFPSSTPQGLATLAAFISSINIAG
Xenopus_tropicalis-ENSXETG00000016591                                       HSPLMSVTNAISGLTAVGGLALMGGGYLPTNTHELLAVLAAFVSSINIAG
Mus_musculus-Uniprot-Q61941                                                 HSPLMSVTNAISGLTAVGGLALMGGHFYPSTTSQSLAALATFISSVNIAG
Rhodospirillum_rubrum_F11-Operon-F11_11245-F11_11240-F11_11235              HSPLMGVTNAISSVIVVGALIATGPEAF--SASKVLGFFAILLASVNIFG
Caenorhabditis_elegans-WBGene00003778-EG                                    HSPLMSVTNAISGTTAAGALCLMGGGLMPQNSAQTMALLATFISSVNIGG
Thermus_thermophilus_HB8-Operon_and_oddEnd-BAD70029_BAD70030_BAD70031       HTPLMSGSNFIHGVVVVGAMVVLGHAET--GLEKLIGFLGVILGAANAAG
Klebsiella_pneumoniae_hvKP1-Operon-G057_17323-G057_17318                    HTPLMSVTNAISGIIVVGALLQIGHG----GWVSFLSFIAVLIASINIFG
Aedes_aegypti-AAEL012616                                                    HSPLMSVTNAISGITAVGGLLMMGGGVTPTNTIETLAASAALISFINIFG
Loxodonta_africana-ENSLAFG00000010443                                       HSPLMSVTNAISGLTAVGGLALMGGHLYPSTTSQGLAALATFISSVNIAG
Anolis_carolinensis-ENSACAG00000010913                                      HSPLMSVTNAISGLTAVGGLALMGGKYLPDSIPQSLAVLAAFVSSVNIAG
Bos_taurus-ENSBTAG00000011885                                               HSPLMSVTNAISGLTAVGGLVLMGGHLYPSTTSQGLAALATFISSVNIAG
Lottiea_gigantea-LotgiG220929                                               HSPLMSVTNAISGITAAGGLLLMGGGYFPSNTIHGLAAAAAFISSINIGG
Pan_troglodytes-ENSPTRG00000016851                                          HSPLMSVTNAISGLTAVGGLALMGGHLYPSTTSQGLAALAAFISSVNIAG
Ciona_savignyi-ENSCSAVG00000010300                                          HSPLMSVTNAISGLTAAGGLFLMGGEYLPSSAPQGLALASTFISSINIVG
Danio_rerio-ENSDARG00000023536                                              HSPLMSVTNAISGLTAVGGLSLMGGGYLPSSTAETLAVLAAFISSVNIAG
```

```
                                                                           580       590       600       610       620
Homo_sapiens-Q13423                                                   GFLVTQRMLDMFKRPTDPPEYNYLYLLPAGTFVGGYLAALYSG--YNIE-
Ciona_intestinalis-ENSCING00000008112                                 GFLVTQRMLDMFKRPDDPPEHNYLYAIPGAAYLGAYYLGTLNGA-SNLH-
Escherichia_coli_str_k_12-pntAB-AAC74675-AAC74674                     GFTVTQRMLKMFRK------------------------NXXXMS-GGLV-
Ornithorhynchus_anatinus-ENSOANG00000014892                           GFLVTQRMLDMFKRPTDPPEYNYLYLIPTGTFVGGYFAALYSG--YDIE-
Xenopus_tropicalis-ENSXETG00000016591                                 GFLVTQRMLDMFKRPTDPPEYNYLYLLPGGALVGGYAAALHSG--YDIE-
Mus_musculus-Uniprot-Q61941                                           GFLVTQRMLDMFKRPTDPPEYNYLYLLPGGTFVGGYLAALYGG--YNIE-
Rhodospirillum_rubrum_F11-Operon-F11_11245-F11_11240-F11_11235        GFIVTQRMLAMFKKKQKXXXMT-----------------------HSLT-
Caenorhabditis_elegans-WBGene00003778-EG                              GFLVTKRMLDMFKRKDDPPEHNYLFSIPAAVFLGGYGYGVYTAA-PLIH-
Thermus_thermophilus_HB8-Operon_and_oddEnd-BAD70029_BAD70030_BAD70031 GYAVTVRMLEMFERKP--------------------GQGGGRXX-MDLI-
Klebsiella_pneumoniae_hvKP1-Operon-G057_17323-G057_17318              GFTVTQRMLKMFRK------------------------GXXXMS-GGLV-
Aedes_aegypti-AAEL012616                                              GFLVTQRMLDMFKRPTDPPEHNYLYGIPGALFLGGYGLAALQGM-PEIH-
Loxodonta_africana-ENSLAFG00000010443                                 GFLVTQRMLDMFKRPTDPPEYNYLYLIPAGTFVGGYLAALYSG--FNIE-
Anolis_carolinensis-ENSACAG00000010913                                GFLVTQRMLDMFKRPTDPPEYNYLYLLPTGVFVGGYGAALQSG--YNIE-
Bos_taurus-ENSBTAG00000011885                                         GFLVTQRMLDMFKRPTDPPEYNYLYLLPAGTFVGGYLASLYSG--YNIE-
Lottiea_gigantea-LotgiG220929                                         GFVVTQRMLDMFKRADDPPEYNKLYGIPAAVFLGGYGYAASTGAYPDIH-
Pan_troglodytes-ENSPTRG00000016851                                    GFLVTQRMLDMFKRPTDPPEYNYLYLLPAGTFVGGYLAALYSG--YNIE-
Ciona_savignyi-ENSCSAVG00000010300                                    GFLVTQRMLDMFKRPDDPPEHNYLYAIPAAAYIGAYFMGSMSGA-SNLHQ
Danio_rerio-ENSDARG00000023536                                        GFLVTQRMLDMFKRPTDPPEYNYLYLLPTGVFVGGYGVALQSG--YNIE-

                                                                                                   630       640
Homo_sapiens-Q13423                                                   ------------------------QIMYLGSGLCCVGALAGLSTQGTARL
Ciona_intestinalis-ENSCING00000008112                                 ------------------------QMMYFISGLCCVGALGGLSKQSTARM
Escherichia_coli_str_k_12-pntAB-AAC74675-AAC74674                     ------------------------TAAYIVAAILFIFSLAGLSKHETSRQ
Ornithorhynchus_anatinus-ENSOANG00000014892                           ------------------------QMMYLGSGLCCVGALAGLSSQGTARL
Xenopus_tropicalis-ENSXETG00000016591                                 ------------------------QMVYLGSGLCCVGALAGLSTQGTARL
Mus_musculus-Uniprot-Q61941                                           ------------------------EIMYLGSGLCCVGALGGLSTQGTARL
Rhodospirillum_rubrum_F11-Operon-F11_11245-F11_11240-F11_11235        ------------------------MAAYIVAGVLFILALRGLSNPESARN
Caenorhabditis_elegans-WBGene00003778-EG                              ------------------------SYAYLGSSLCCVGALAGLSSQSTARV
Thermus_thermophilus_HB8-Operon_and_oddEnd-BAD70029_BAD70030_BAD70031 ------------------------QAAYFVVAILFIVGLKRMAHPTTAKS
Klebsiella_pneumoniae_hvKP1-Operon-G057_17323-G057_17318              ------------------------TAAYIVAAILFIFSLAGLSKHETSQQ
Aedes_aegypti-AAEL012616                                              ------------------------QMAYLASSLCCIGALVGLSSQKTSRL
Loxodonta_africana-ENSLAFG00000010443                                 ------------------------QIMYLGSGLCCVGALAGLSTQGTARL
Anolis_carolinensis-ENSACAG00000010913                                ------------------------QMMYLGSGLCCVGALAGLSTQGTARL
Bos_taurus-ENSBTAG00000011885                                         ------------------------QIMYLGSGLCCVGALAGLSTQGTARL
Lottiea_gigantea-LotgiG220929                                         ------------------------QMIYLGAGLSCVGALTGLSSQKTARV
Pan_troglodytes-ENSPTRG00000016851                                    ------------------------QIMYLGSGLCCVGALAGLSTQGTARL
Ciona_savignyi-ENSCSAVG00000010300                                    VFWNCDLYTNPYVNLILDVNLPFLQMMYFISGLCCVGALGGLSKQSTARM
Danio_rerio-ENSDARG00000023536                                        ------------------------QMMYLGSGLCCVGALGGLSTQSTARL
```

```
                                                                       650       660       670       680
Homo_sapiens-Q13423                                                  GNALGMIGVAGGLAATLGVLKPGPELLAQMSGAMALGGTIG--------LT
Ciona_intestinalis-ENSCING00000008112                                GNALGIIGVSGGIAATLGCIAPSPALLTQMVSAMAVGGVAG--------FG
Escherichia_coli_str_k_12-pntAB-AAC74675-AAC74674                    GNNFGIAGMAIALIATI--FGPDTGNVGWILLAMVIGGAIG--------IR
Ornithorhynchus_anatinus-ENSOANG00000014892                          GNALGIMGVAGGLAATLGSLKPSLELLGQMSGAMALGGTIG--------LT
Xenopus_tropicalis-ENSXETG00000016591                                GNALGMMGVAGGIVATLGALKPSPELLAQMSGAMALGGTLG--------LT
Mus_musculus-Uniprot-Q61941                                          GNALGMIGVAGGLAATLGGLKPDPQLLAQMSGAMAMGGTIG--------LT
Rhodospirillum_rubrum_F11-Operon-F11_11245-F11_11240-F11_11235       GNRMGMVGMAIAILTTL--LSPSVQAYAWIVLAIAIGGAIG--------TV
Caenorhabditis_elegans-WBGene00003778-EG                             GNALGIIGVTGGIGATLGLLQPDFNTLCQMGGSVAMGSLIG--------LG
Thermus_thermophilus_HB8-Operon_and_oddEnd-BAD70029_BAD70030_BAD70031 GIVWAGWGMVLAVLATF--FWPGMGNFALILLALLLGSAVA--------WW
Klebsiella_pneumoniae_hvKP1-Operon-G057_17323-G057_17318             GNYFGIAGMAIALIATI--LGPDAGNVGWIILAMVIGGAIG--------IR
Aedes_aegypti-AAEL012616                                             GNALGMIGVTGGIAATLGHMAPSTEVLMQMGGVAGIGGLLG--------TI
Loxodonta_africana-ENSLAFG00000010443                                GNALGMIGVAGGLAATLGGLKPCPELLAQMSGAMALGGTIG--------LT
Anolis_carolinensis-ENSACAG00000010913                               GNALGMIGVAGGLAATLGGLKPSPELLGQMSGAMALGGTIG--------LT
Bos_taurus-ENSBTAG00000011885                                        GNALGMIGVAGGLAATLGGLKPCPELLAQMSGAMALGGTIG--------LT
Lottiea_gigantea-LotgiG220929                                        GNVLGMIGVGSGVTATLGIIKPDLDTLVQMSTCMGLGGLIG--------GL
Pan_troglodytes-ENSPTRG00000016851                                   GNALGMIGVAGGLAATLGVLKPGPELLAQMSGAMALGGTIG--------LT
Ciona_savignyi-ENSCSAVG00000010300                                   GNALGIIGVSGGIVATLGCIAPSTALLTQMCGAMGVGGLTGDLYQQTCLA
Danio_rerio-ENSDARG00000023536                                       GNALGMIGVAGGIAATFGVLKPSPELMAQMSAAMAVGGTAG--------LT
```

```
                                                                    690       700       710       720       730
Homo_sapiens-Q13423                                                  IAKRIQISDLPQLVAAFHSLVGLAAVLTCIAEYIIEYPH--FATDAA-AN
Ciona_intestinalis-ENSCING00000008112                                IARSIEITSLPQLVAAFHSLVGLAAVMTCVAEFMIEHPH--LAENEM-GN
Escherichia_coli_str_k_12-pntAB-AAC74675-AAC74674                    LAKKVEMTEMPELVAILHSFVGLAAVLVGFNSYLHHDA---GMAPIL-VN
Ornithorhynchus_anatinus-ENSOANG00000014892                          IAKRIQITDLPQLVAAFHSLVGLAAVLTCVAEYMVEYPH--FALDPA-AN
Xenopus_tropicalis-ENSXETG00000016591                                IAKRIQISDLPQLVAAFHSLVGLAAVLTCVAEYMIEYPH--FATDPA-AN
Mus_musculus-Uniprot-Q61941                                          IAKRIQISDLPQLVAAFHSLVGLAAVLTCMAEYIVEYPH--FAMDAT-SN
Rhodospirillum_rubrum_F11-Operon-F11_11245-F11_11240-F11_11235       IAKKVLMTALPQLVAAFHSLVGMAAVLVATGALLNPEAYGIGSAGAI-HA
Caenorhabditis_elegans-WBGene00003778-EG                             IANRIKVTDLPQLVAAFHSFVGLAATLTCLANFIQEHPH--FLEDPSNAA
Thermus_thermophilus_HB8-Operon_and_oddEnd-BAD70029_BAD70030_BAD70031 AAVRVAMTDMPQMVAIYNGMGGGAAATIAAVELLKGAFE---------NT
Klebsiella_pneumoniae_hvKP1-Operon-G057_17323-G057_17318             LAKKVEMTEMPELVAILHSFVGLAAVLVGFNSYLQHET---GMEQIL-VN
Aedes_aegypti-AAEL012616                                             IAKRIQITDLPQLVAAFHSLVGAAAVLTCVATYMHDFPT--LATDPA-AN
Loxodonta_africana-ENSLAFG00000010443                                IAKRIQISDLPQLVAAFHSLVGLAAVLTCIAEYIVEYPH--FATDAA-AN
Anolis_carolinensis-ENSACAG00000010913                               IAKRIQISDLPQLVAAFHSLVGLAAVLTCVAEYMIEYPH--FATNEA-AN
Bos_taurus-ENSBTAG00000011885                                        IAKRIQISDLPQLVAAFHSLVGLAAVLTCIAEYIIEYPH--FATDAA-AN
Lottiea_gigantea-LotgiG220929                                        AAKRIEVTDLPQMVALFHSLVGAAAVLTCFSNHLLEQPH--FATDPA-AG
Pan_troglodytes-ENSPTRG00000016851                                   IAKRIQISDLPQLVAAFHSLVGLAAVLTCIAEYIIEYPH--FATDAA-AN
Ciona_savignyi-ENSCSAVG00000010300                                   IAKSIEITTLPQLVAAFHSLVGLAAVMTCMAEFMIEHPH--LAENEM-GN
Danio_rerio-ENSDARG00000023536                                       IAKKIQISDLPQLVAAFHSLVGLAAVLTCVAEYMVEYPH--FATDPA-AN
```

```
                                                                     740       750       760       770       780
Homo_sapiens-Q13423                                                  LTKIVAYLGTYIGGVTFSGSLIAYGKLQGLLKSAPLLLPGRHLLNAGLLA
Ciona_intestinalis-ENSCING00000008112                                MIKFVAYLGTYIGGVTFTGSLVAYGKLQGILDSAAVLLPGRHMLNAGLLT
Escherichia_coli_str_k_12-pntAB-AAC74675-AAC74674                    IHLTEVFLGIFIGAVTFTGSVVAFGKLCGKISSKPLMLPNRHKMNLAALV
Ornithorhynchus_anatinus-ENSOANG00000014892                          LTKIVAYLGTYIGGVTFSGSLIAYGKLQGILNSAPLLLPGRHLLNLGLLT
Xenopus_tropicalis-ENSXETG00000016591                                LTKIVAYLGTYIGGVTFSGSLVAYGKLQGLLNSAPLMLPGRHMLNAGLLA
Mus_musculus-Uniprot-Q61941                                          FTKIVAYLGTYIGGVTFSGSLVAYGKLQGILKSAPLLLPGRHALNAGLLA
Rhodospirillum_rubrum_F11-Operon-F11_11245-F11_11240-F11_11235       GSLVEMSLGLAVGAITFSGSVIAFGKLQGLIAGKPVTFPMQHPLNAVLGI
Caenorhabditis_elegans-WBGene00003778-EG                             AAKLALFLGTYIGGVTFTGSLMAYGKLQGILASAPTYLPARHVLNGALLA
Thermus_thermophilus_HB8-Operon_and_oddEnd-BAD70029_BAD70030_BAD70031 GLMALAILGGLIGSVAFTGSLIAFAKLQGIMKSRPILFPGQKAVNALVLA
Klebsiella_pneumoniae_hvKP1-Operon-G057_17323-G057_17318             IHLTEVFLGIFIGAVTFTGSVVAFGKLRGKISSRPLMLPNRHKLNLAALV
Aedes_aegypti-AAEL012616                                             VLKTALFLGTYIGGVTFSGSLVAYGKLQGILNSSPLLLPGRHMINAGLLA
Loxodonta_africana-ENSLAFG00000010443                                LTKIVAYLGTYIGGVTFSGSLVAYGKLQGILKSAPLLLPGRHLLNAGLLA
Anolis_carolinensis-ENSACAG00000010913                               LTKIVAYLGTYIGGVTFSGSLVAYGKLQGILNSAPLLLPGRHLLNAGLLT
Bos_taurus-ENSBTAG00000011885                                        LTKIVAYLGTYIGGVTFSGSLVAYGKLQGILKSAPLLLPGRHLLNAGLLA
Lottiea_gigantea-LotgiG220929                                        VLKGALFAGTFIGGVTFTGSLIAFGKLQGVLKSDPLLLPGRNALNAGMGL
Pan_troglodytes-ENSPTRG00000016851                                   LTKIVAYLGTYIGGVTFSGSLIAYGKLQGLLKSAPLLLPGRHLLNAGLLA
Ciona_savignyi-ENSCSAVG00000010300                                   MIKFVAYLGTYIGGVTFTGSLVAYGKLQGILDSAAVMLPGRHMMNAGLLT
Danio_rerio-ENSDARG00000023536                                       LTKIVAYLGTYIGGVTFSGSLVAYGKLQGLLNSAPLMLPGRHALNATLMA
```

```
                                                                      790       800       810      820        830
Homo_sapiens-Q13423                                                  ASVGGIIPFMVDPSFTTGITCLGSVSALSAVMGVTLTAAIGGADMPVVIT
Ciona_intestinalis-ENSCING00000008112                                GSFLPMIPFMLDNSYNTGIMCLGSTALLSTVMGTTLTVAIGGADMPVVIT
Escherichia_coli_str_k_12-pntAB-AAC74675-AAC74674                    VSFLLLIVFVRTDSVGLQVLALLIMTAIALVFGWHLVASIGGADMPVVVS
Ornithorhynchus_anatinus-ENSOANG00000014892                          ASAGGIIPYMLDPSFTTGIMCLGSVSALSAIMGVTLTAAIGGADMPVVIT
Xenopus_tropicalis-ENSXETG00000016591                                ASVGGIIPYMLDPSYTTGLTCLGSVSALSAVMGVTLTAAIGGADMPVVIT
Mus_musculus-Uniprot-Q61941                                          ASVGGIIPFMADPSFTTGITCLGSVSGLSTLMGVTLTAAIGGADMPVVIT
Rhodospirillum_rubrum_F11-Operon-F11_11245-F11_11240-F11_11235       LLVVLLVVFAATESHTAYFALM----ILAFALGFLLIIPIGGADMPVVIS
Caenorhabditis_elegans-WBGene00003778-EG                             GNVGALGTYMYSTDFGTGMSMLGGTVGLSSLMGVTLTMAIGGADMPIVIT
Thermus_thermophilus_HB8-Operon_and_oddEnd-BAD70029_BAD70030_BAD70031 LTLVLGLSLLWNDATASIVLFF----LLALLFGVLMTLPIGGGDMPVAIS
Klebsiella_pneumoniae_hvKP1-Operon-G057_17323-G057_17318             VSFILMVIFVRSDSTGTQVLCLLVMTAIALAFGWHLVASIGGADMPVVVS
Aedes_aegypti-AAEL012616                                             GNLGAMAMFYMEPTMAGGLGLLGTTAALSTAMGVTLTAAIGGADMPVVIT
Loxodonta_africana-ENSLAFG00000010443                                ASVGGIIPFMMDPSFTMGITCLGSVSALSAIMGVTLTAAIGGADMPVVIT
Anolis_carolinensis-ENSACAG00000010913                               ASAGGMIPYMIDPSYTTGLTCLGSVSALSAVMGVTLTAAIGGADMPVVIT
Bos_taurus-ENSBTAG00000011885                                        GSVGGIIPFMMDPSFTTGITCLGSVSALSAVMGVTLTAAIGGADMPVVIT
Lottiea_gigantea-LotgiG220929                                        SSVAALGTYMVSEDPSTLLGMLGVTTTLSSILGVTLTAAIGGADMPVVIT
Pan_troglodytes-ENSPTRG00000016851                                   ASVGGIIPFMVDPSFATGITCLGSVSALSAVMGVTLTAAIGGADMPVVIT
Ciona_savignyi-ENSCSAVG00000010300                                   GSFLPMIPFIKKN-------------------GTTLTMAIGGADMPVVIT
Danio_rerio-ENSDARG00000023536                                       ASVGGMIPYMLDPSYTTGITCLGSVSALSAVMGLTLTAAIGGADMPVVIT
```

```
                                                                    840       850       860       870       880
Homo_sapiens-Q13423                                                 VLNSYSGWALCAEGFLLNNNLLTIVGALIGSSGAILSYIMCVAMNRSLAN
Ciona_intestinalis-ENSCING00000008112                               VLNSYSGWALCAEGFLLNNNLLTIVGALIGSSGAILSYIMCVAMNRSLMS
Escherichia_coli_str_k_12-pntAB-AAC74675-AAC74674                   MLNSYSGWAAAAAGFMLSNDLLIVTGALVGSSGAILSYIMCKAMNRSFIS
Ornithorhynchus_anatinus-ENSOANG00000014892                         VLNSYSGWALCAEGFLLNNNLLTIVGALIGSSGAILSYIMCVAMNRSLAN
Xenopus_tropicalis-ENSXETG00000016591                               VLNSYSGWALCAEGFLLNNNLLTIVGALIGSSGAILSYIMCVAMNRSLAN
Mus_musculus-Uniprot-Q61941                                         VLNSYSGWALCAEGFLLNNNLLTIVGALIGSSGAILSYIMCVAMNRSLAN
Rhodospirillum_rubrum_F11-Operon-F11_11245-F11_11240-F11_11235      MLNSYSGWAAAGIGFTLGNPLLIIAGALVGSSGAILSYIMCKGMNRSIFN
Caenorhabditis_elegans-WBGene00003778-EG                            VLNSYSGWALCAEGFMLDNSLLTVLGALIGSSGAILSHIMCKAMNRSLLN
Thermus_thermophilus_HB8-Operon_and_oddEnd-BAD70029_BAD70030_BAD70031 FYNAFTGMAVGFEGFAVGNPALMVAGTLVGAAGTLLTVLMARAMNRSVWS
Klebsiella_pneumoniae_hvKP1-Operon-G057_17323-G057_17318            MLNSYSGWAAAAAGFMLSNDLLIVTGALVGSSGAILSYIMCKAMNRSFIS
Aedes_aegypti-AAEL012616                                            VLNSYSGWALCAEGFMLNNNLMTIVGALIGSSGAILSYIMCKAMNRSLAN
Loxodonta_africana-ENSLAFG00000010443                               VLNSYSGWALCAEGFLLNNNLLTIVGALIGSSGAILSYIMCVAMNRSLAN
Anolis_carolinensis-ENSACAG00000010913                              VLNSYSGWALCAEGFLLNNNLLTIVGALIGSSGAILSYIMCVAMNRSLAN
Bos_taurus-ENSBTAG00000011885                                       VLNSYSGWALCAEGFLLNNNLLTIVGALIGSSGAILSYIMCVAMNRSLAN
Lottiea_gigantea-LotgiG220929                                       VLNSYSGWALCAEGFMLNNNLMTIVGAMIGSSGAILSYIMCVAMNRSIFN
Pan_troglodytes-ENSPTRG00000016851                                  VLNSYSGWALCAEGFLLNNNLLTIVGALIGSSGAILSYIMCVAMNRSLAN
Ciona_savignyi-ENSCSAVG00000010300                                  VLNSYSGWALCAEGFLLNNNLLTIVGALIGSSGAILSYIMCVAMNRSLMN
Danio_rerio-ENSDARG00000023536                                      VLNSYSGWALCAEGFLLNNNLLTIVGALIGSSGAILSYIMCVAMNRSLAN
```

```
                                                                    890       900       910       920       930
Homo_sapiens-Q13423                                                 VILGGYGTTSTAGGKPMEISG--THTEINLDNAIDMIREANSIIITPGYG
Ciona_intestinalis-ENSCING00000008112                               VILGGYGTSSTGGGKPMEITG--THTEVNVDDTVQMIKDAENIIIVPGYG
Escherichia_coli_str_k_12-pntAB-AAC74675-AAC74674                   VIAGGFGTDGSSTGDDQEV-G--EHREITAEETAELLKNSHSVIITPGYG
Ornithorhynchus_anatinus-ENSOANG00000014892                         VILGGYGTTSTAGGKPMEITG--THTEINLDNATEMIKEANNIIITPGYG
Xenopus_tropicalis-ENSXETG00000016591                               VILGGYGTTSTAGGKPMEITG--THTEINLENAVEYIREANNIIITPGYG
Mus_musculus-Uniprot-Q61941                                         VILGGYGTTSTAGGKPMEISG--THTEINLDNAVEMIREANSIVITPGYG
Rhodospirillum_rubrum_F11-Operon-F11_11245-F11_11240-F11_11235      VILGGFGSEGGVAAAGGAA-GDRSVKAGSAEDAAFIMKNASKVIIVPGYG
Caenorhabditis_elegans-WBGene00003778-EG                            VILGGVGTKSKGTGEAKAIEG--TAKEIAPVETADMLLNARSVIIIPGYG
Thermus_thermophilus_HB8-Operon_and_oddEnd-BAD70029_BAD70030_BAD70031 VLVGGFGVEQEAG----EVKG--SLKPIDVEDAAVMLAYAGKVVFVPGYG
Klebsiella_pneumoniae_hvKP1-Operon-G057_17323-G057_17318            VIAGGFGTDGSSSGGDEEV-G--EHREISAEETAEMLKNSHSVIITPGYG
Aedes_aegypti-AAEL012616                                            VILGGYGTSSTGGGKPAEIVG--THTEVNVDGVVDMIKNSKNIIITPGYG
Loxodonta_africana-ENSLAFG00000010443                               VILGGYGTTSTAGGKPMEISG--THTEINLDNAIDMIREANSIIITPGYG
Anolis_carolinensis-ENSACAG00000010913                              VILGGYGTTSTAGGKPMEITG--THTEVNVDSAVEMIKEANNIIITPGYG
Bos_taurus-ENSBTAG00000011885                                       VILGGYGTTSTAGGKPMEISG--THTEINLDNAIDMIREANSIIITPGYG
Lottiea_gigantea-LotgiG220929                                       VILGGYGTSSTGKGKPMEITG--THTEVNVDDAVEMINEAKNIIIVPGYG
Pan_troglodytes-ENSPTRG00000016851                                  VILGGYGTTSTAGGKPMEISG--THTEINLDNAIDMIREANSIIITPGYG
Ciona_savignyi-ENSCSAVG00000010300                                  VILGGYGTSSTGSGKPMEITG--THTEVTVDDTTQMIKDAENIIIVPGYG
Danio_rerio-ENSDARG00000023536                                      VILGGYGTSSTGTGKPMEITG--THTEVNVDQTVDLIKEAHNIIIVPGYG
```

```
                                                                 940       950                 960   970
Homo_sapiens-Q13423                                              LCAAKAQYPIADLVKMLTEQGKKV-------------RFGIHPVAGRMPG
Ciona_intestinalis-ENSCING00000008112                            LCVAKAQYPIADMVNRLRDAGKNV-------------RFGIHPVAGRMPG
Escherichia_coli_str_k_12-pntAB-AAC74675-AAC74674                MAVAQAQYPVAEITEKLRARGINV-------------RFGIHPVAGRLPG
Ornithorhynchus_anatinus-ENSOANG00000014892                      LCAAKAQYPIADLVKMLTEQGKKVRXVRKLASQGGKLRFGIHPVAGRMPG
Xenopus_tropicalis-ENSXETG00000016591                            LCAAKAQYPIADLVKMLKDEGKHV-------------RFGIHPVAGRMPG
Mus_musculus-Uniprot-Q61941                                      LCAAKAQYPIADLVKMLTEQGKKV-------------RFGIHPVAGRMPG
Rhodospirillum_rubrum_F11-Operon-F11_11245-F11_11240-F11_11235   MAVAQAQHALREMADVLKKEGVEV-------------SYAIHPVAGRMPG
Caenorhabditis_elegans-WBGene00003778-EG                         LCAAQAQYPIAQLVKELQQRGVRV-------------RFAIHPVAGRMPG
Thermus_thermophilus_HB8-Operon_and_oddEnd-BAD70029_BAD70030_BAD70031 MALSQAQHKLKELADLLEARGVEV-------------KFAIHPVAGRMPG
Klebsiella_pneumoniae_hvKP1-Operon-G057_17323-G057_17318         MAVAQAQYPVAEITEKLRARGIKV-------------RFGIHPVAGRLPG
Aedes_aegypti-AAEL012616                                         LCVAKAQYPIAEMVNILKSHGKKV-------------RFGIHPVAGRMPG
Loxodonta_africana-ENSLAFG00000010443                            LCAAKAQYPIADLVKMLTEQGKKV-------------RFGIHPVAGRMPG
Anolis_carolinensis-ENSACAG00000010913                           LCAAKAQYPIADLVKMLCEQGKNV-------------RFGIHPVAGRMPG
Bos_taurus-ENSBTAG00000011885                                    LCAAKAQYPIADLVKMLSEQGKKV-------------RFGIHPVAGRMPG
Lottiea_gigantea-LotgiG220929                                    LCVAKAQYPIAEMVSTLVKKGHNV-------------RFGIHPVAGRMPG
Pan_troglodytes-ENSPTRG00000016851                               LCAAKAQYPIADLVKMLTEQGKKV-------------RFGIHPVAGRMPG
Ciona_savignyi-ENSCSAVG00000010300                               LCVAKAQYPVADMVNKLREAGKNV-------------RFGIHPVAGRMPG
Danio_rerio-ENSDARG00000023536                                   LCAAKAQYPIADLVKSLTDQGKKV-------------RFGIHPVAGRMPG
```

```
                                                                     980       990       1000      1010      1020
Homo_sapiens-Q13423                                              QLNVLLAEAGVPYDIVLEMDEINHDFPDTDLVLVIGANDTVNSAAQEDPN
Ciona_intestinalis-ENSCING00000008112                            QLNVLLAEAGVPYDVVLEMDEINDDFSDTDLVLVVGANDTVNSAAQEDPN
Escherichia_coli_str_k_12-pntAB-AAC74675-AAC74674                HMNVLLAEAKVPYDIVLEMDEINDDFADTDTVLVIGANDTVNPAAQDDPK
Ornithorhynchus_anatinus-ENSOANG00000014892                      QLNVLLAEAGVPYDIVLEMDEINHDFPDTDLVLVIGANDTVNSAAQEDPN
Xenopus_tropicalis-ENSXETG00000016591                            QLNVLLAEAGVPYDIVLEMDEINEDFPETDLVLVIGANDTVNSAAQEDPN
Mus_musculus-Uniprot-Q61941                                      QLNVLLAEAGVPYDIVLEMDEINSDFPDTDLVLVIGANDTVNSAAQEDPN
Rhodospirillum_rubrum_F11-Operon-F11_11245-F11_11240-F11_11235   HMNVLLAEANVPYDEVFELEEINSSFQTADVAFVIGANDVTNPAAKTDPS
Caenorhabditis_elegans-WBGene00003778-EG                         QLNVLLAEAGVPYDIVEEMEEINEDFKETDVALVIGSNDTINSAAEDDPN
Thermus_thermophilus_HB8-Operon_and_oddEnd-BAD70029_BAD70030_BAD70031 HMNVLLAEAGVDYDKLKDLEEINPEFPTVDVAVVIGANDVVNPAAR-RPG
Klebsiella_pneumoniae_hvKP1-Operon-G057_17323-G057_17318         HMNVLLAEAKVPYDIVLEMDEINDDFSDTDTVLVIGANDTVNPAAQDDPK
Aedes_aegypti-AAEL012616                                         QLNVLLAEAGVPYDDVLEMEEINEDFTDTDLVLVIGANDTVNSAAEDDPN
Loxodonta_africana-ENSLAFG00000010443                            QLNVLLAEAGVPYDIVLEMDEINHDFPDTDLVLVIGANDTVNSAAQEDPN
Anolis_carolinensis-ENSACAG00000010913                           QLNVLLAEAGVPYDIVLEMDEINHDFPATDLVLVIGANDTVNSAAQEDPN
Bos_taurus-ENSBTAG00000011885                                    QLNVLLAEAGVPYDIVLEMDEINHDFPDTDLVLVIGANDTVNSAAQEDPN
Lottiea_gigantea-LotgiG220929                                    QLNVLLAEAGVPYDIVQEMDELNDDFPQTDLALVIGANDTVNSAAEEDPN
Pan_troglodytes-ENSPTRG00000016851                               QLNVLLAEAGVPYDIVLEMDEINHDFPDTDLVLVIGANDTVNSAAQEDPN
Ciona_savignyi-ENSCSAVG00000010300                               QLNVLLAEAGVPYDVVLEMDEINEDFSDTDLVLVVGANDTVNSAAQEDPN
Danio_rerio-ENSDARG00000023536                                   QLNVLLAEAGVPYDVVLEMDEINEDFPETDLVLVIGANDTVNSAAQEDPN
```

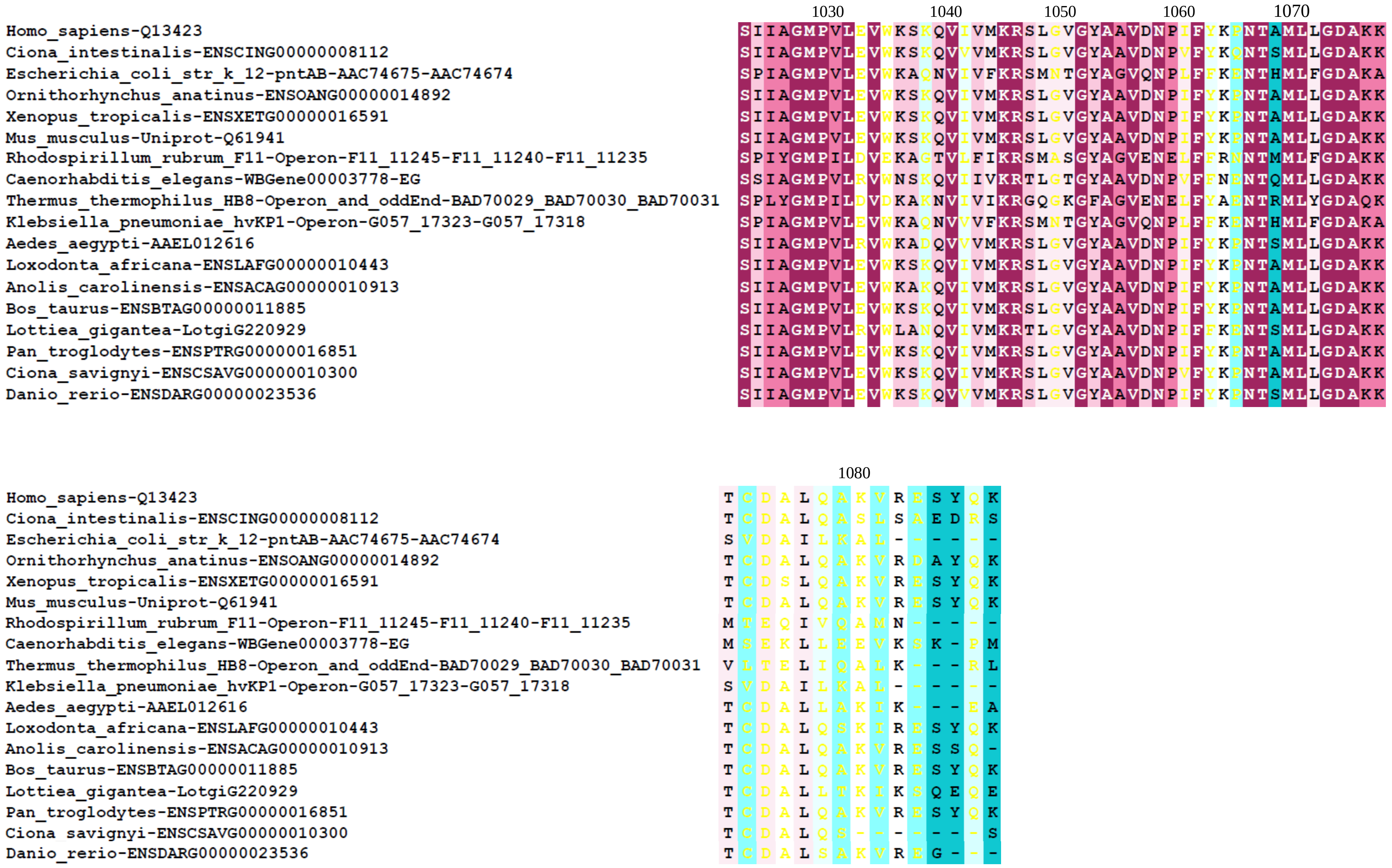
1030
1040
1050
1060
1070
Homo_sapiens-Q13423
Ciona_intestinalis-ENSCING00000008112
Escherichia_coli_str_k_12-pntAB-AAC74675-AAC74674
Ornithorhynchus_anatinus-ENSOANG00000014892
Xenopus_tropicalis-ENSXETG00000016591
Mus_musculus-Uniprot-Q61941
Rhodospirillum_rubrum_F11-Operon-F11_11245-F11_11240-F11_11235
Caenorhabditis_elegans-WBGene00003778-EG
Thermus_thermophilus_HB8-Operon_and_oddEnd-BAD70029_BAD70030_BAD70031
Klebsiella_pneumoniae_hvKP1-Operon-G057_17323-G057_17318
Aedes_aegypti-AAEL012616
Loxodonta_africana-ENSLAFG00000010443
Anolis_carolinensis-ENSACAG00000010913
Bos_taurus-ENSBTAG00000011885
Lottiea_gigantea-LotgiG220929
Pan_troglodytes-ENSPTRG00000016851
Ciona_savignyi-ENSCSAVG00000010300
Danio_rerio-ENSDARG00000023536
1080

Supp. Figure S2 Domain I model analysis using PROCHECK and ProSA structure validation tools.

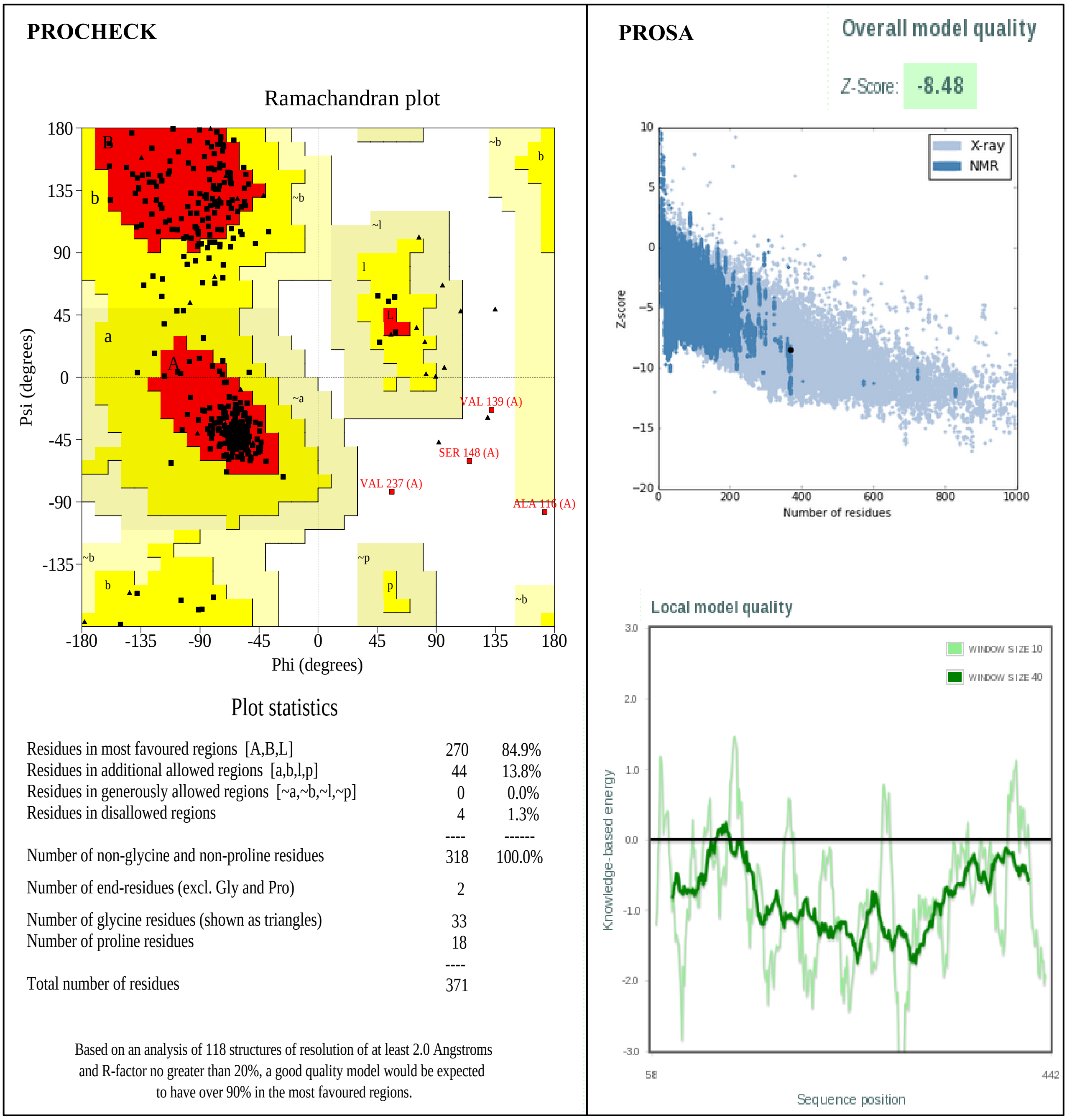

Supp. Figure S3 Predicted NAD binding site in domain I of the H-NNT. A) 3D structure of domain I (in grey) bound to NAD (presented as blue spheres). The NAD cleft is depicted in magenta. B) Schematic 2D representation of domain I bound to NAD. The diagram was generated with Ligplot+ using the human model of domain I. The bold orange bonds belong to the ligand, whereas the thin purple bonds to H-NNT. Hydrogen bonds between H-NNT and NAD are presented as dashed lines. H-NNT residues predicted to make hydrophobic contacts are presented as spiked arcs. C) List of all residues predicted to form the NAD binding site. The conservation score of H-NNT amino acids is reported.

*, residue not shown by Ligplot+ diagram, but in close proximity to NAD molecule (distance ≤ 5A).

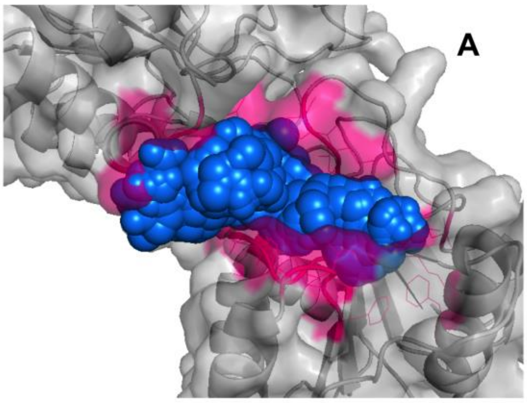

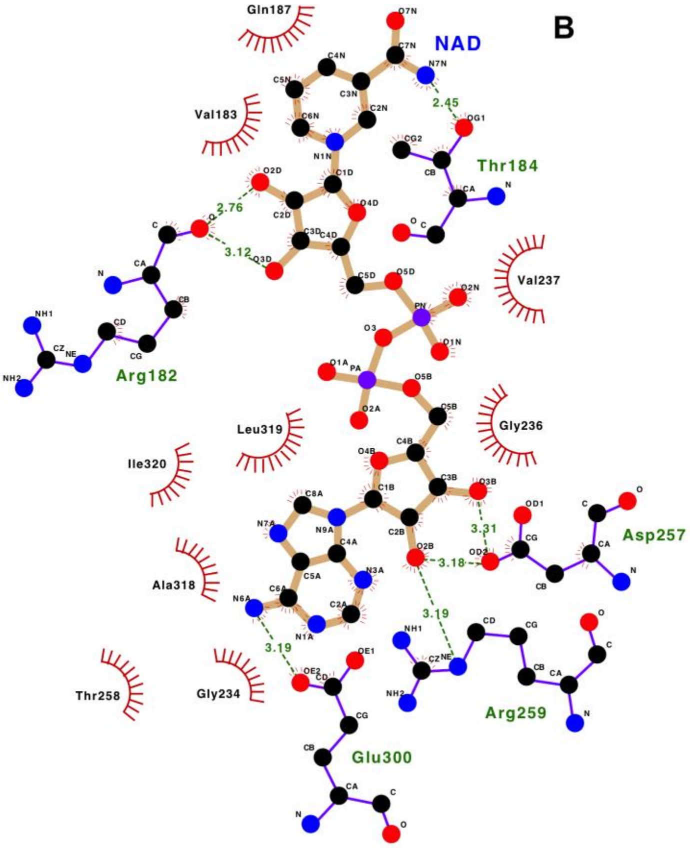

C

Res	Aa	Conservation score
182	ARG	R 100.0
183	VAL	V 61.1
184	THR	T 66.7
187	GLN	Q 100.0
233 *	VAL	V 27.8
234	GLY	G 100.0
235 *	GLY	G 77.8
236	GLY	G 100.0
237	VAL	V 100.0
238 *	ALA	A 100.0
256 *	PHE	F 88.9
257	ASP	D 100.0
258	THR	T 88.9
259	ARG	R 100. 0
300	GLU	E 88.9
304 *	PHE	F 88.9
317 *	THR	T 100.0
318	ALA	A 100.0
319	LEU	L 94.4
320	ILE	I 94.4
321 *	PRO	P 100.0
328 *	LEU	L 100.0

Supp. Figure S4A The NAD binding site in *E. coli* NNT. The plot was generated using LigPlot+ and PDB 2bru.

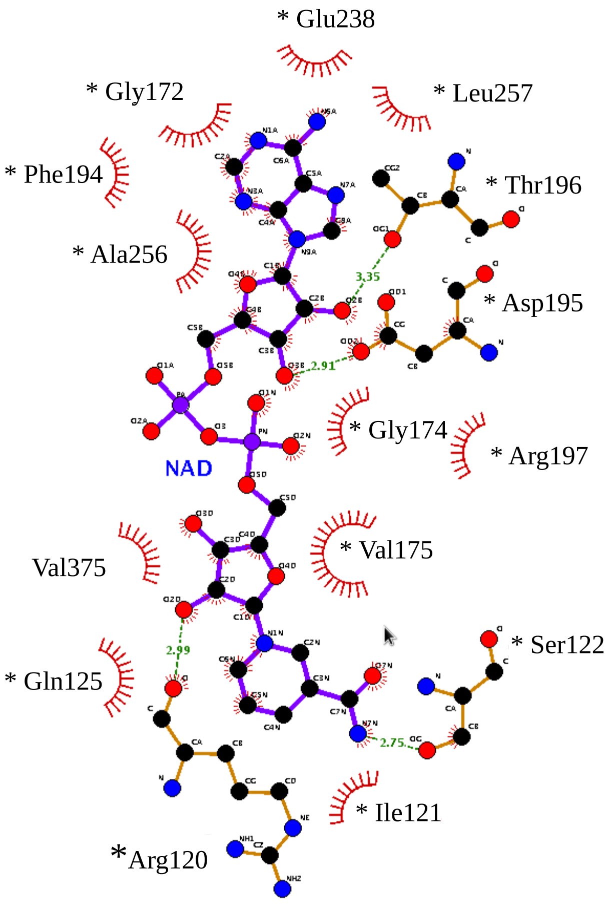

*, conserved residues between *E. coli* and human NNT.

Supp. Figure S4B Sequence alignment for domain I between human, *E. coli* and *T. thermophilus* NTT (Tt-NTT) is presented. The secondary structure elements of the H-NNT model (H-NNT_ss) are presented on top of the alignment. Residues forming the NAD binding site in H-NNT are highlighted in cyan.

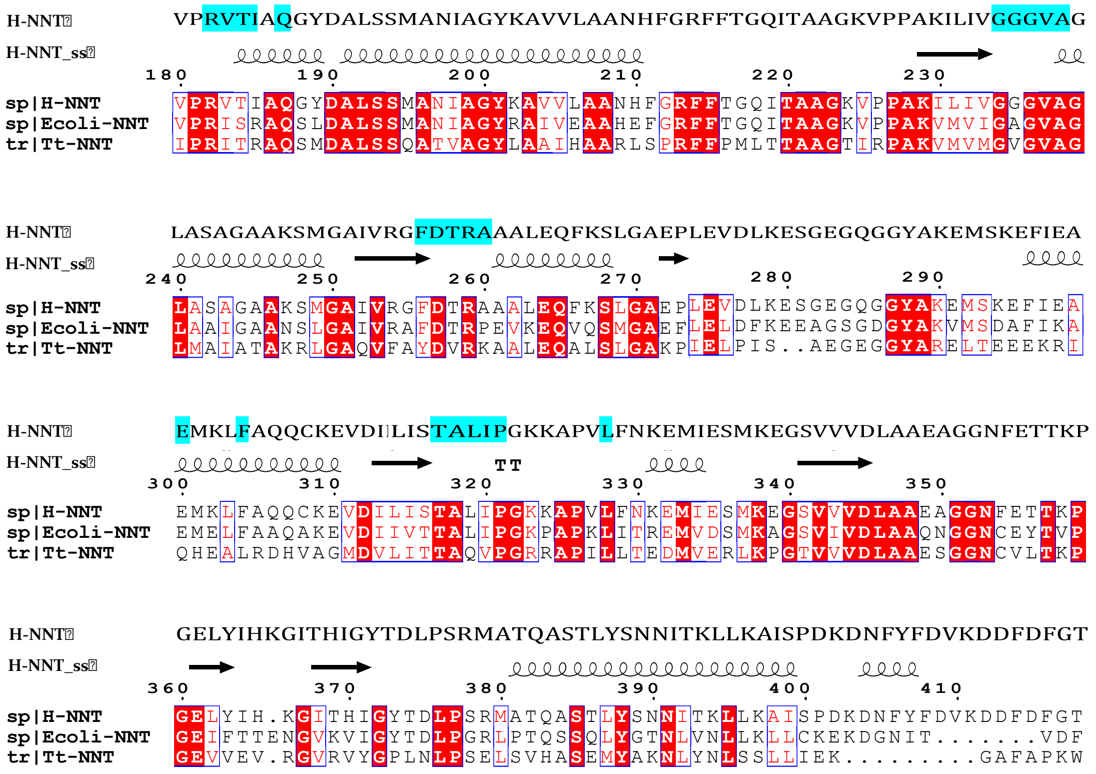

Supp. Figure S5 NNT catalytic site. The amino acid sequence of the RQD loop in H-NNT domain I, is presented. The RQD logo shows conservation of individual amino acids and was generated using Weblogo.

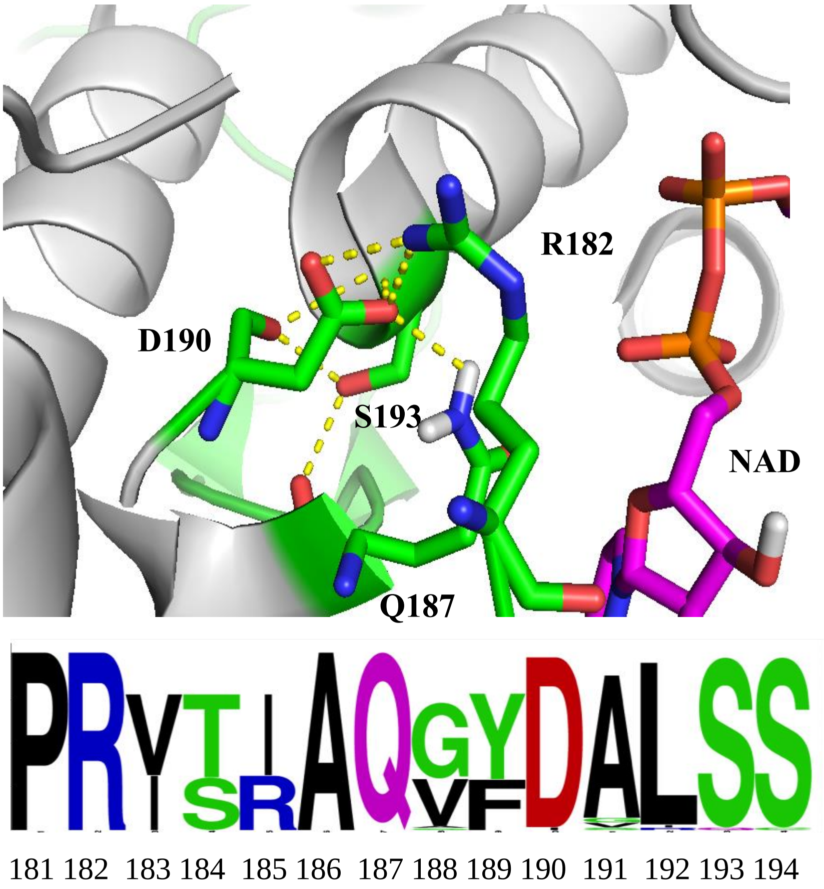

Supp. Figure S6 Schematic diagram of H-NNT Domain II. Transmembrane helices (TM) predicted to form the proton canal are highlighted in blue. Highlighted in red circles are highly conserved residues, which are functionally or structurally important, as demonstrated by mutagenesis studies in *E. coli* NNT (as described in the main text).

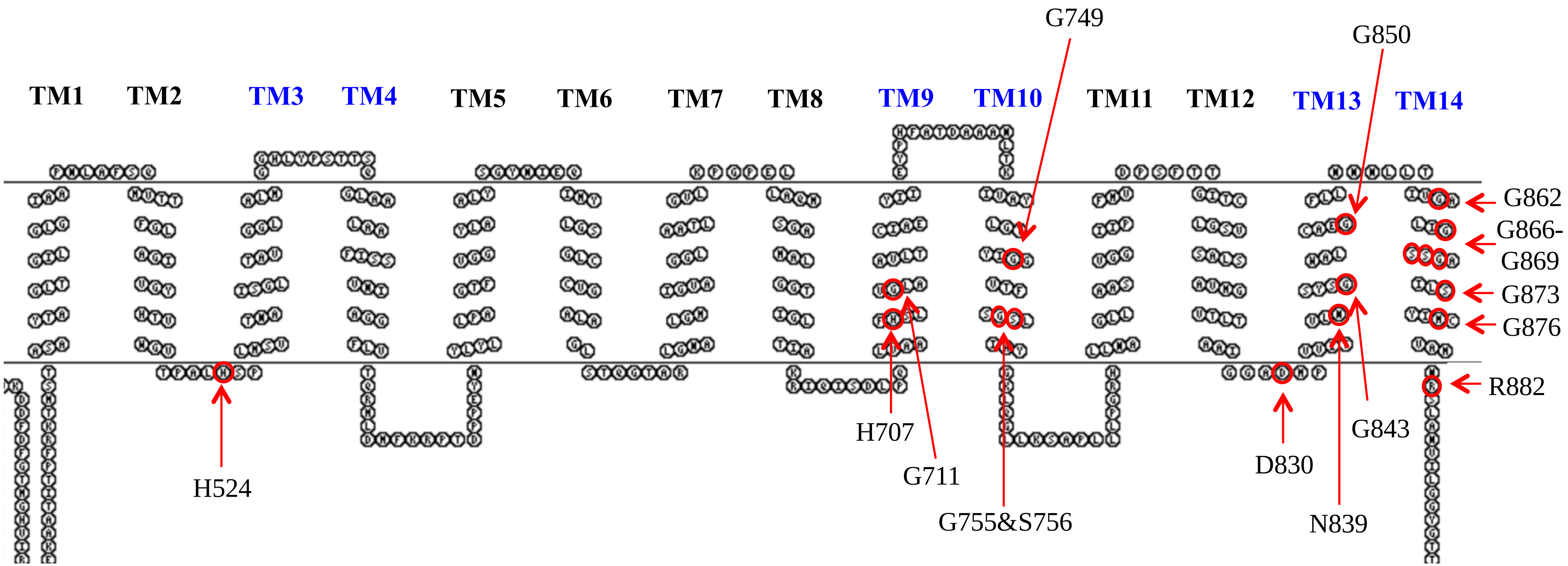

Supp. Figure S7 Domain II model analysis using PROCHECK and ProSA structure validation tools.

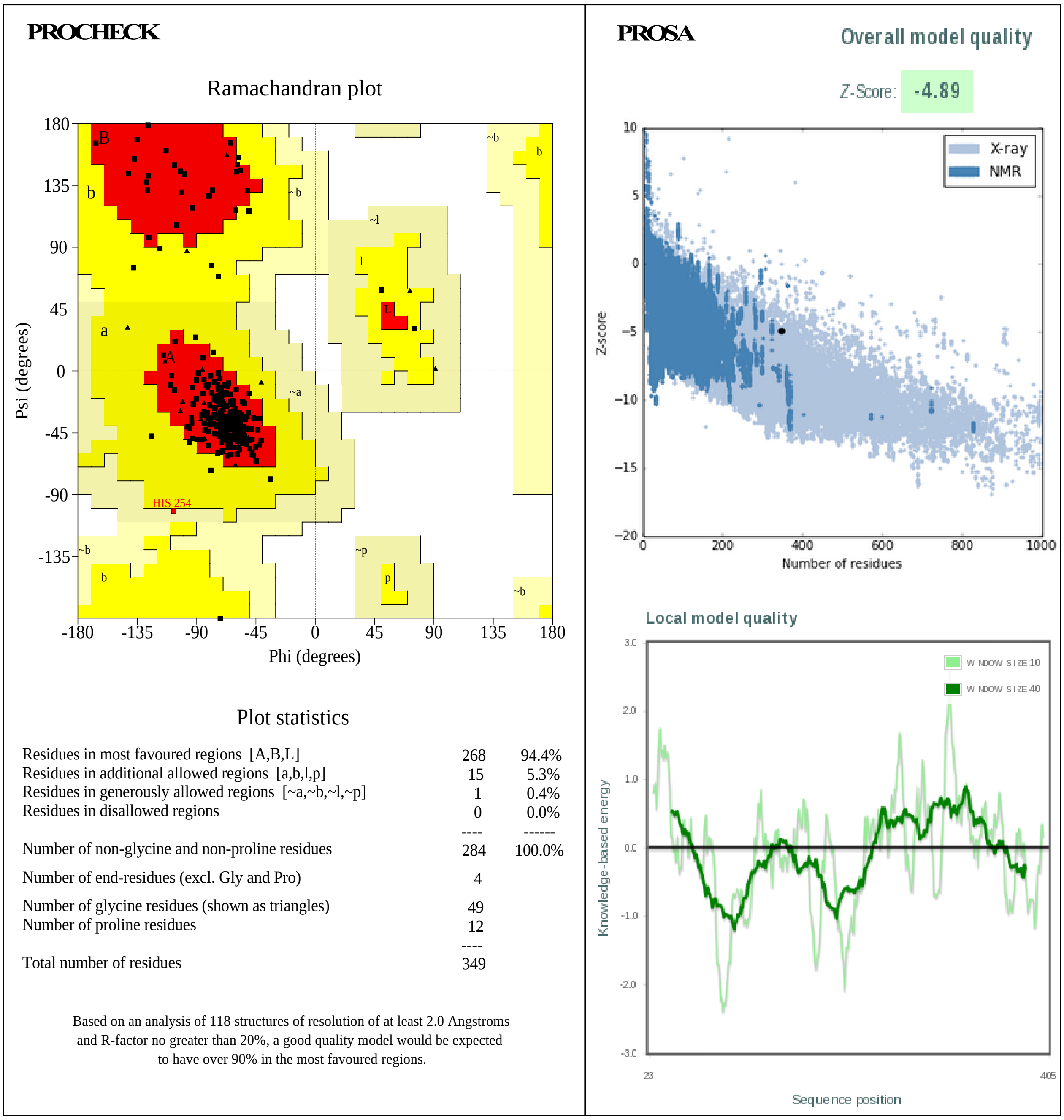

Supp. Figure S8 Structural analysis of amino acid substitutions identified in H-NNT and described in this study. The wild type residue (in green) is presented on the left and the amino acid substitution (in green) on the right. The dotted line indicates the presence of a chemical bond.

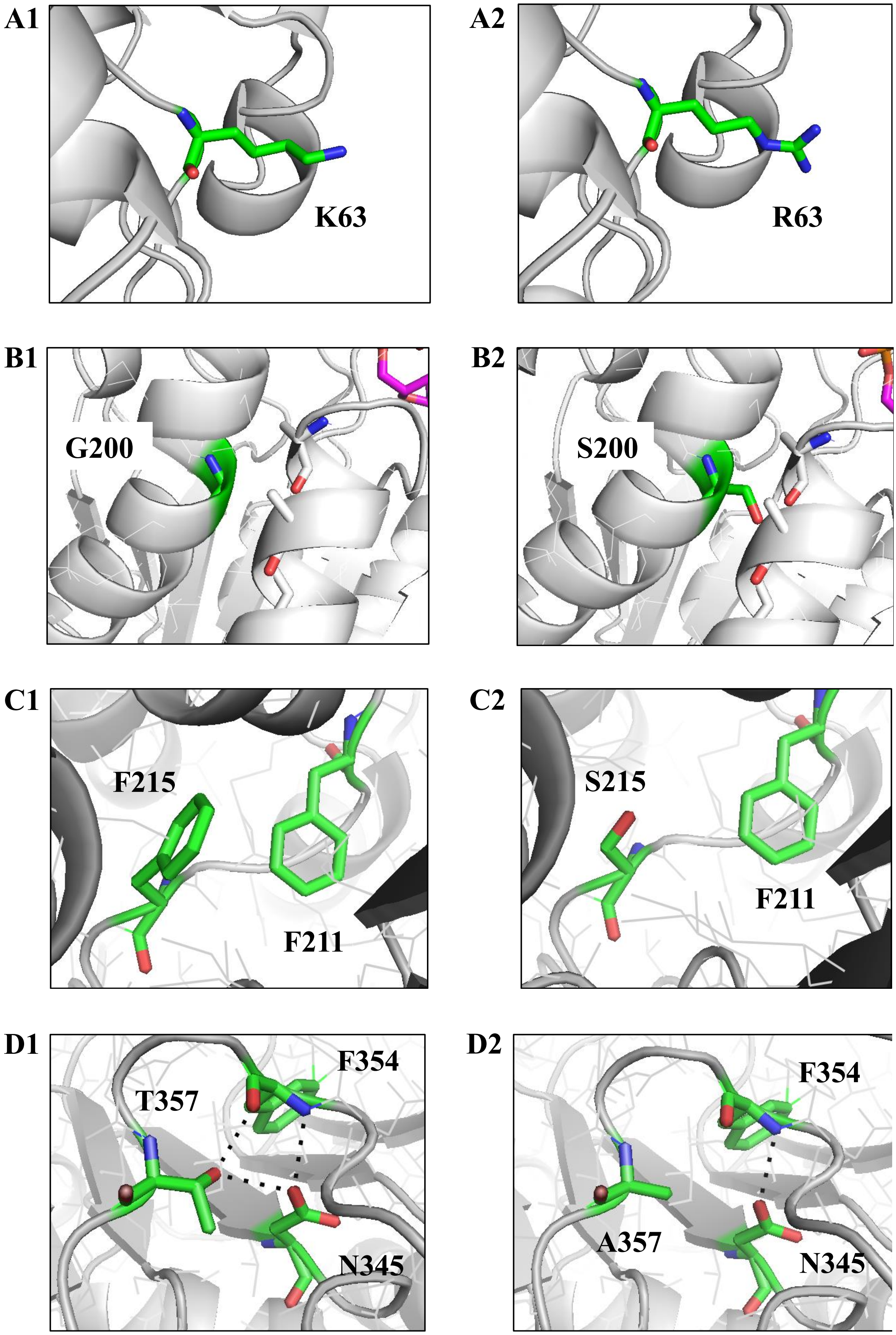

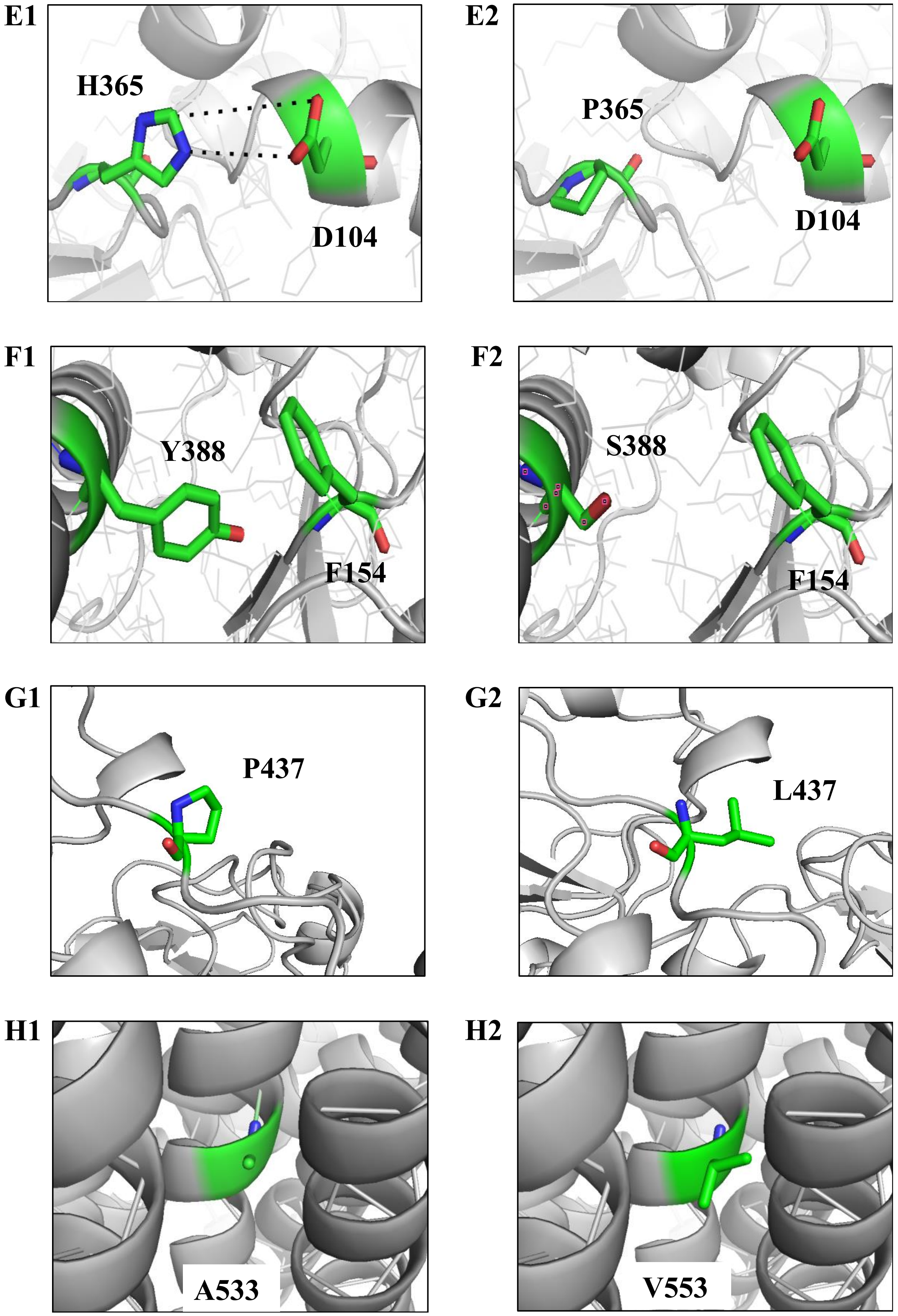
E1
H365
D104
E2
P365
D104
F1
Y388
F154
F2
S388
F154
G1
P437
G2
L437
H1
A533
H2
V553

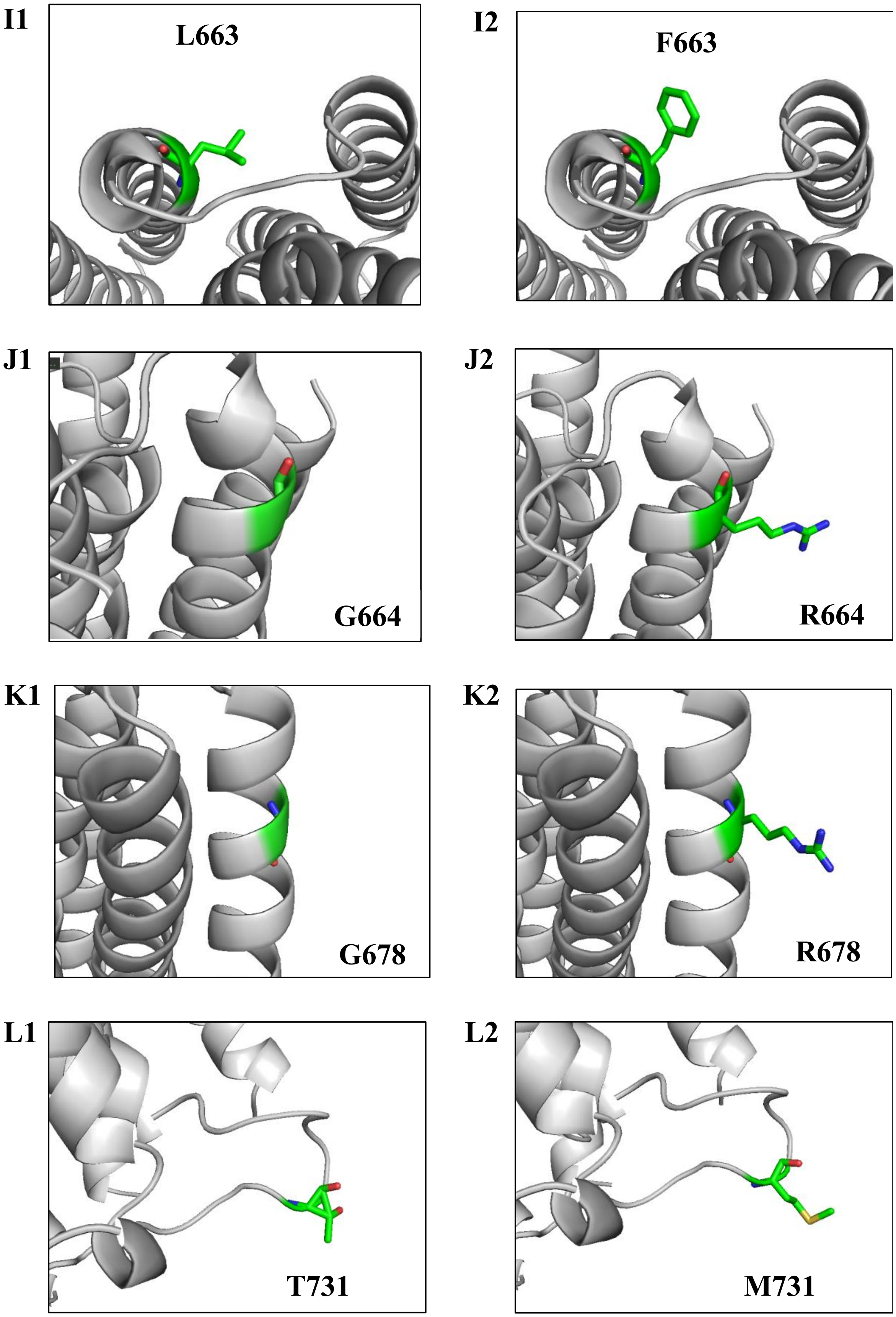
I1
L663
I2
F663
J1
G664
J2
R664
K1
G678
K2
R678
L1
T731
L2
M731

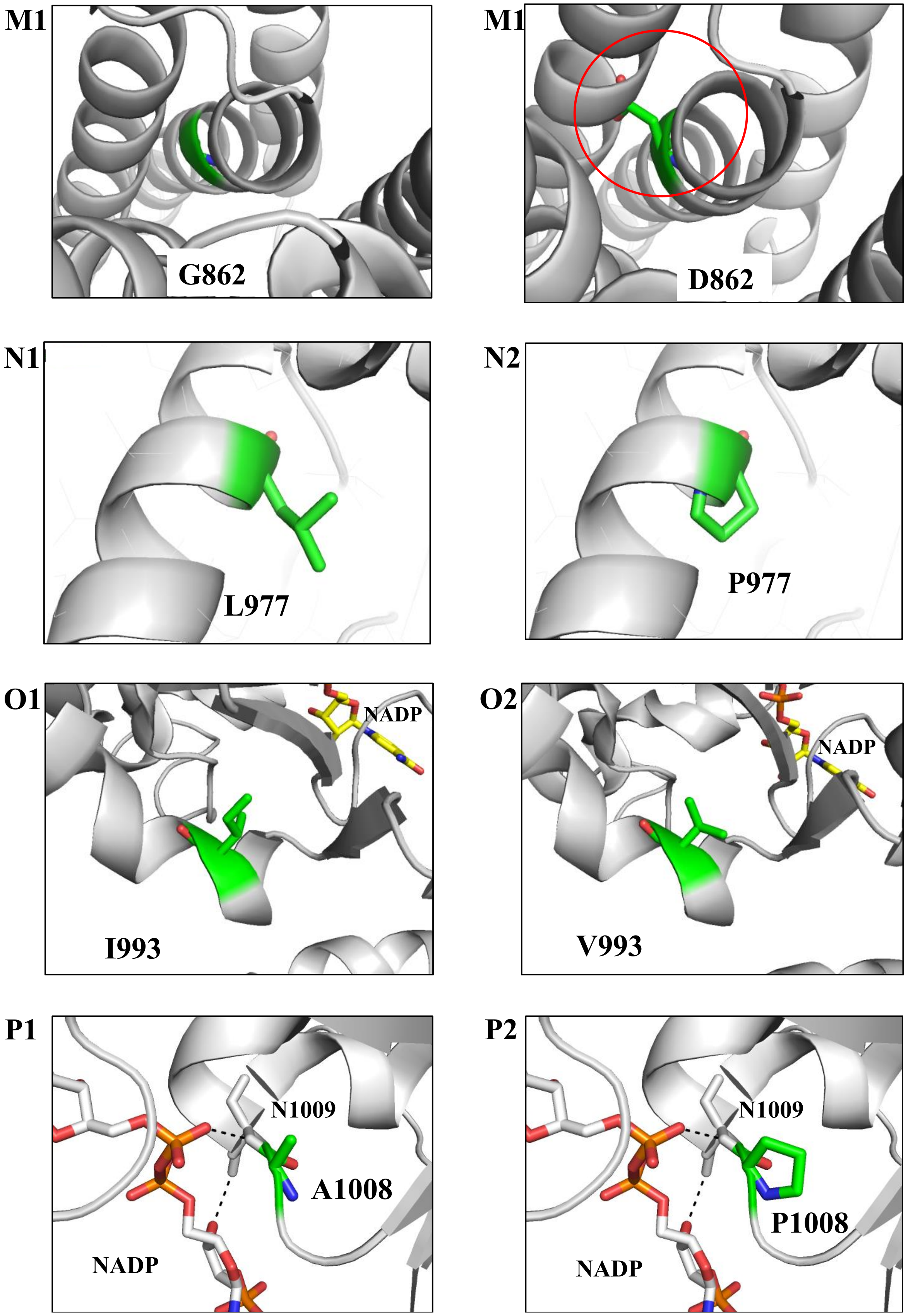
M1
G862
M1
D862
N1
L977
N2
P977
O1
NADP
I993
O2
NADP
V993
P1
N1009
A1008
NADP
P2
N1009
P1008
NADP

Q1

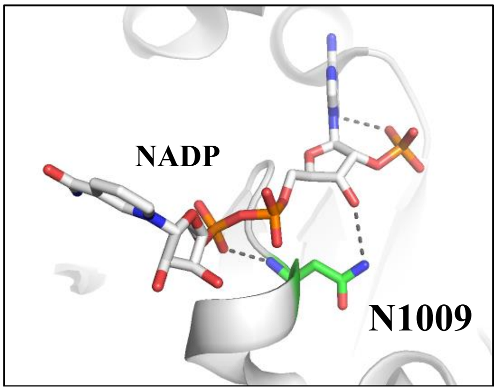

Q2

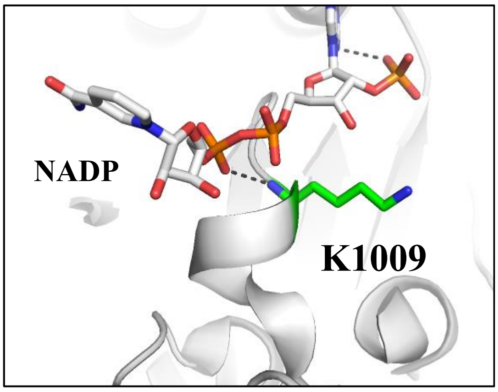

Supp. Table S1 Sequence-structure alignment for H-NNT. The sequence alignment between human, E. coli and T. thermophilus NTT (Tt-NTT) is presented. The secondary structure elements of the H-NNT model (H-NNT_ss) are presented on top of the alignment. The structural coverage of the H-NNT model (H-NNT_cov) shows residues that were modelled (in black) and residues that were not modelled (in blue). Functionally or structurally important residues that are described in the main text are highlighted in grey, deleterious amino acid substitutions in green and rare variants reported in EXAC database in cyan.

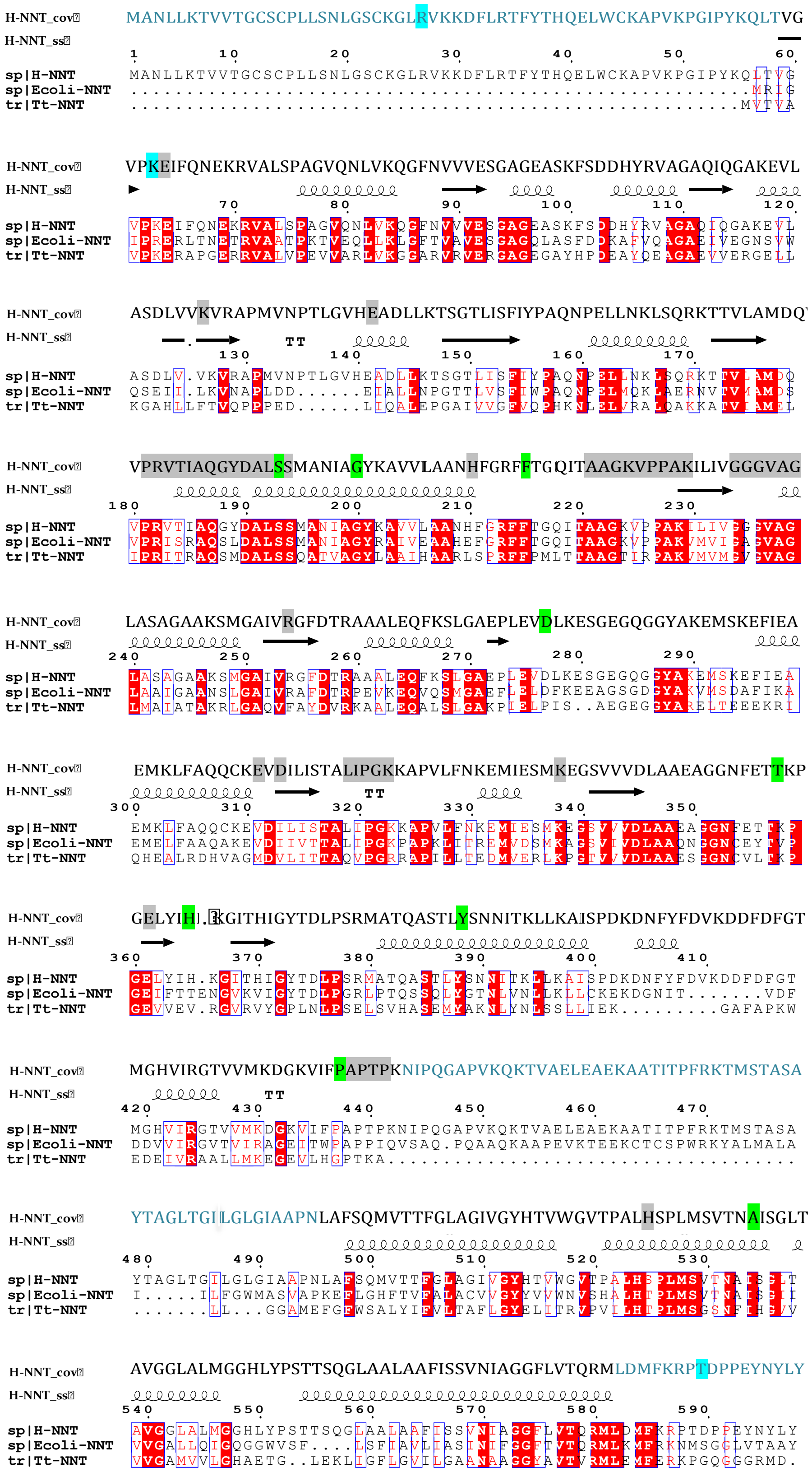

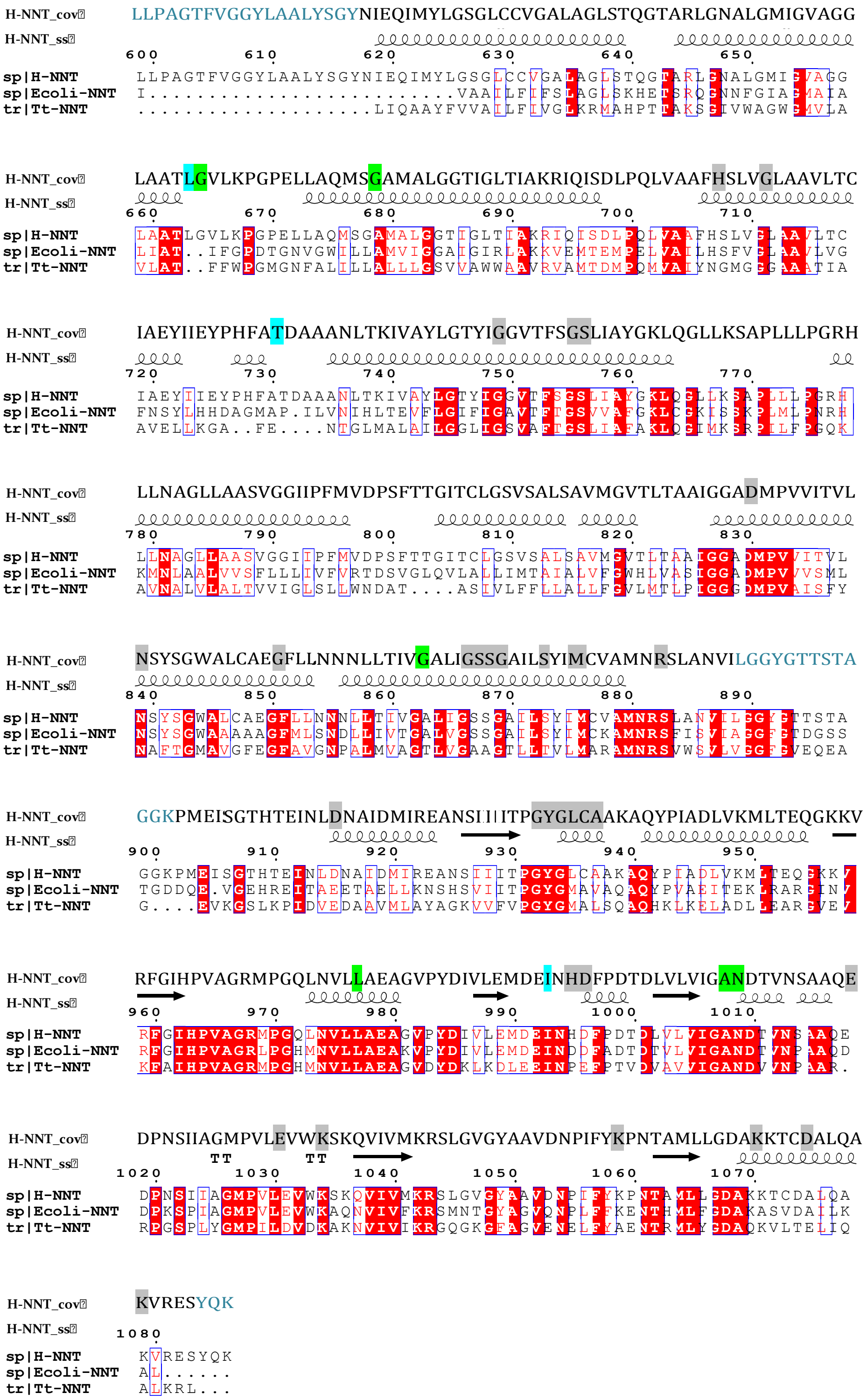

α-helices are displayed as squiggles, β-strands as arrows and strict β-turns as TT letters.

Supp. Table S2 Degree of conservation of the amino-acid sites between H-NNT and its close homologues.

Position in Alignment	ConSurf Class	Amino acid in Alignment
1	9	Met(100.0%)
2	6*	Ala(90.0%), Val(10.0%)
3	1	Asn(55.6%), Ser(22.2%), His(11.1%), Gly(11.1%)
4	5*	Leu(90.9%), Ile(9.1%)
5	8	Leu(100.0%)
6	4*	Lys(54.5%), Arg(45.5%)
7	1*	Thr(66.7%), Ser(16.7%), Val(8.3%), Ala(8.3%)
8	2*	Val(66.7%), Lys(16.7%), Arg(8.3%), Ala(8.3%)
9	1	Val(63.6%), Thr(9.1%), Gln(9.1%), Gly(9.1%), Ala(9.1%)
10	1	Thr(63.6%), Ala(18.2%), Ser(9.1%), Met(9.1%)
11	4*	Gly(45.5%), Ser(36.4%), Leu(18.2%)
12	2*	Cys(81.8%), Arg(9.1%), Leu(9.1%)
13	6*	Ser(81.8%), Leu(18.2%)
14	2*	Cys(54.5%), Ser(36.4%), Ala(9.1%)
15	1	Pro(72.7%), Thr(18.2%), Asp(9.1%)
16	1	Leu(54.5%), Phe(18.2%), Tyr(9.1%), Val(9.1%), Asn(9.1%)
17	1	Phe(54.5%), Leu(36.4%), Gly(9.1%)
18	2*	Ser(63.6%), Asn(18.2%), Gly(18.2%)
19	1	Asn(63.6%), Ser(18.2%), Gly(9.1%), Asp(9.1%)
20	1	Leu(72.7%), Val(9.1%), Arg(9.1%), Ala(9.1%)
21	1	Gly(54.5%), Ile(18.2%), Gln(9.1%), Met(9.1%), Phe(9.1%)
22	1	Ser(66.7%), Cys(22.2%), Pro(11.1%)
23	1	Cys(44.4%), Tyr(11.1%), Ser(11.1%), Leu(11.1%), His(11.1%), Ala(11.1%)
24	4*	Lys(88.9%), Arg(11.1%)
25	1	Val(44.4%), Thr(22.2%), Gly(22.2%), Ala(11.1%)
26	1	Leu(72.7%), Val(9.1%), Lys(9.1%), His(9.1%)
27	1	Pro(50.0%), Arg(20.0%), Thr(10.0%), Lys(10.0%), His(10.0%)

28	1	Val(40.0%), Gly(40.0%), Leu(10.0%), Ile(10.0%)
29	6*	Lys(81.8%), Ser(18.2%)
30	1	Lys(60.0%), Val(10.0%), Thr(10.0%), Arg(10.0%), Ala(10.0%)
31	1	Asp(27.3%), Pro(18.2%), Asn(18.2%), Lys(18.2%), Trp(9.1%), Thr(9.1%)
32	1	Phe(54.5%), Cys(27.3%), Tyr(9.1%), Pro(9.1%)
33	1	Leu(54.5%), Val(18.2%), Ile(18.2%), Thr(9.1%)
34	1*	Arg(72.7%), Gln(9.1%), Leu(9.1%), His(9.1%)
35	1	Thr(54.5%), Pro(18.2%), Met(9.1%), Gly(9.1%), Phe(9.1%)
36	1	Phe(54.5%), Leu(36.4%), Ser(9.1%)
37	1	Arg(36.4%), His(27.3%), Tyr(18.2%), Leu(9.1%), Lys(9.1%)
38	1*	Thr(66.7%), Ser(16.7%), Ile(8.3%), Asp(8.3%)
39	2*	His(75.0%), Thr(8.3%), Ser(8.3%), Gly(8.3%)
40	1	Gln(58.3%), Ser(16.7%), Arg(16.7%), Met(8.3%)
41	1	Ala(25.0%), Val(16.7%), Leu(16.7%), Glu(16.7%), Thr(8.3%), Arg(8.3%), Ile(8.3%)
42	1	Leu(75.0%), Tyr(8.3%), Arg(8.3%), Lys(8.3%)
43	1	Trp(72.7%), Tyr(9.1%), Arg(9.1%), Leu(9.1%)
44	1	Cys(72.7%), Leu(18.2%), Ser(9.1%)
45	1	Gln(33.3%), Lys(33.3%), Ser(8.3%), Asn(8.3%), His(8.3%), Phe(8.3%)
46	1	Ala(50.0%), Ser(25.0%), Lys(16.7%), Arg(8.3%)
47	1	Pro(58.3%), Val(8.3%), Asn(8.3%), Leu(8.3%), Asp(8.3%), Ala(8.3%)
48	1	Val(58.3%), Gly(16.7%), Thr(8.3%), Glu(8.3%), Ala(8.3%)
49	2*	Lys(75.0%), Gln(16.7%), Ser(8.3%)
50	5*	Pro(91.7%), Lys(8.3%)
51	6*	Gly(92.9%), Lys(7.1%)
52	2*	Ile(64.3%), Val(14.3%), Asn(14.3%), Thr(7.1%)
53	6*	Pro(92.9%), Glu(7.1%)
54	8	Tyr(100.0%)
55	4*	Lys(85.7%), Ser(7.1%), Asn(7.1%)
56	4*	Gln(64.3%), Asn(14.3%), Asp(14.3%), Lys(7.1%)
57	3*	Leu(50.0%), Ile(22.2%), Met(16.7%), Val(11.1%)
58	5*	Thr(72.2%), Arg(11.1%), Lys(11.1%), Ile(5.6%)

59	7	Val(61.1%), Ile(38.9%)
60	6	Gly(83.3%), Ala(16.7%)
61	6	Val(83.3%), Ile(16.7%)
62	8	Pro(94.4%), Ala(5.6%)
63	4*	Lys(77.8%), Arg(16.7%), Gln(5.6%)
64	9	Glu(100.0%)
65	4*	Ile(61.1%), Arg(27.8%), Val(5.6%), Ser(5.6%)
66	1	Phe(61.1%), Leu(22.2%), Trp(5.6%), Arg(5.6%), Ala(5.6%)
67	3*	Gln(50.0%), Pro(22.2%), Glu(11.1%), Ala(11.1%), Thr(5.6%)
68	4*	Asn(72.2%), Gly(16.7%), Gln(5.6%), His(5.6%)
69	9	Glu(100.0%)
70	2*	Lys(66.7%), Arg(16.7%), Thr(11.1%), Asp(5.6%)
71	9	Arg(100.0%)
72	9	Val(94.4%), Ala(5.6%)
73	9	Ala(94.4%), Ser(5.6%)
74	1	Leu(66.7%), Ala(16.7%), Ile(11.1%), Val(5.6%)
75	5*	Ser(72.2%), Thr(16.7%), Val(11.1%)
76	9	Pro(100.0%)
77	6*	Ala(72.2%), Lys(11.1%), Glu(11.1%), Asn(5.6%)
78	1	Gly(61.1%), Val(16.7%), Thr(11.1%), Ala(11.1%)
79	8	Val(94.4%), Thr(5.6%)
80	1	Gln(50.0%), Ala(22.2%), Glu(16.7%), Thr(5.6%), Lys(5.6%)
81	1	Ala(38.9%), Asn(16.7%), Gln(11.1%), Val(5.6%), Thr(5.6%), Arg(5.6%), Leu(5.6%), Lys(5.6%), Ile(5.6%)
82	9	Leu(100.0%)
83	1	Val(61.1%), Leu(11.1%), Lys(11.1%), Ile(11.1%), Ser(5.6%)
84	8	Lys(94.4%), Gly(5.6%)
85	1	Gln(50.0%), Leu(16.7%), Lys(11.1%), Ile(11.1%), Asn(5.6%), Gly(5.6%)
86	9	Gly(100.0%)
87	4*	Phe(77.8%), Tyr(11.1%), Ile(5.6%), Ala(5.6%)
88	1	Asn(61.1%), Ser(11.1%), Lys(11.1%), Thr(5.6%), Arg(5.6%), Glu(5.6%)
89	9	Val(100.0%)

90	1	Val(44.4%), Asn(16.7%), Ile(11.1%), Ala(11.1%), Arg(5.6%), Leu(5.6%), Lys(5.6%)
91	8	Val(88.9%), Ile(11.1%)
92	9	Glu(100.0%)
93	1	Ser(61.1%), Glu(16.7%), Arg(5.6%), Gln(5.6%), Lys(5.6%), Ala(5.6%)
94	1*	Gly(72.2%), Asn(22.2%), Ser(5.6%)
95	9	Ala(100.0%)
96	9	Gly(100.0%)
97	1	Glu(61.1%), Val(16.7%), Gln(5.6%), Lys(5.6%), Phe(5.6%), Asp(5.6%)
98	1	Ala(50.0%), Leu(16.7%), Gly(11.1%), Ser(5.6%), Lys(5.6%), Glu(5.6%), Asp(5.6%)
99	8	Ser(55.6%), Ala(44.4%)
100	3*	Lys(61.1%), Ser(16.7%), Glu(11.1%), Tyr(5.6%), Gly(5.6%)
101	4*	Phe(83.3%), Tyr(5.6%), Ile(5.6%), His(5.6%)
102	2*	Ser(61.1%), Pro(11.1%), Asp(11.1%), Thr(5.6%), Arg(5.6%), Lys(5.6%)
103	6	Asp(83.3%), Asn(16.7%)
104	2*	Asp(61.1%), Glu(16.7%), Ala(16.7%), Lys(5.6%)
105	2*	His(33.3%), Ala(22.2%), Met(16.7%), Asp(11.1%), Gln(5.6%), Leu(5.6%), Glu(5.6%)
106	5	Tyr(83.3%), Phe(11.1%), Leu(5.6%)
107	1	Arg(33.3%), Thr(16.7%), Ala(16.7%), Val(11.1%), Lys(11.1%), Ser(5.6%), Gln(5.6%)
108	1	Ala(38.9%), Glu(33.3%), Val(5.6%), Ser(5.6%), Arg(5.6%), Gln(5.6%), Lys(5.6%)
109	7	Ala(83.3%), Val(5.6%), Ser(5.6%), Leu(5.6%)
110	9	Gly(100.0%)
111	9	Ala(100.0%)
112	1	Gln(38.9%), Thr(16.7%), Glu(16.7%), Asn(11.1%), Ser(5.6%), Lys(5.6%), Asp(5.6%)
113	8	Ile(88.9%), Val(11.1%)
114	1	Gln(44.4%), Val(22.2%), Gly(11.1%), Ala(11.1%), Arg(5.6%), Lys(5.6%)
115	1	Gly(44.4%), Ser(22.2%), Glu(11.1%), Asp(11.1%), Thr(5.6%), Lys(5.6%)
116	1	Thr(27.8%), Ala(22.2%), Lys(16.7%), Gly(11.1%), Val(5.6%), Arg(5.6%), Met(5.6%), His(5.6%)
117	1	Lys(55.6%), Ser(11.1%), Asn(11.1%), Gln(5.6%), Gly(5.6%), Glu(5.6%), Asp(5.6%)
118	1	Glu(55.6%), Asp(27.8%), Ser(5.6%), Gln(5.6%), Ala(5.6%)
119	6*	Val(77.8%), Leu(16.7%), Ala(5.6%)
120	1	Leu(50.0%), Phe(27.8%), Trp(11.1%), Tyr(5.6%), Ser(5.6%)

121	2*	Ala(50.0%), Gln(22.2%), Ser(11.1%), Asn(5.6%), Lys(5.6%), His(5.6%)
122	8	Ser(83.3%), Ala(11.1%), Thr(5.6%)
123	7	Asp(88.9%), His(5.6%), Glu(5.6%)
124	2*	Leu(44.4%), Ile(33.3%), Val(22.2%)
125	3*	Val(61.1%), Leu(22.2%), Ile(11.1%), Met(5.6%)
126	5*	Leu(50.0%), Val(38.9%), Trp(5.6%), Phe(5.6%)
127	8	Lys(94.4%), Thr(5.6%)
128	9	Val(100.0%)
129	7	Arg(77.8%), Gln(11.1%), Asn(11.1%)
130	3*	Ala(72.2%), Pro(11.1%), Arg(5.6%), Gln(5.6%), Gly(5.6%)
131	9	Pro(100.0%)
132	1	Met(61.1%), Glu(11.1%), Thr(5.6%), Ser(5.6%), Pro(5.6%), Asn(5.6%), Leu(5.6%)
133	1	Leu(33.3%), Val(27.8%), Asp(16.7%), Glu(11.1%), Thr(5.6%), Met(5.6%)
134	3*	Asn(61.1%), Asp(16.7%), Thr(5.6%), Ser(5.6%), Glu(5.6%), Ala(5.6%)
135	7	Pro(100.0%)
136	1*	Thr(60.0%), Ala(30.0%), Glu(10.0%)
137	4*	Leu(90.0%), Glu(10.0%)
138	8	Gly(100.0%)
139	4*	Val(80.0%), Thr(10.0%), Ala(10.0%)
140	6*	His(90.0%), Asp(10.0%)
141	8	Glu(94.4%), Leu(5.6%)
142	5	Ala(50.0%), Ile(33.3%), Val(16.7%)
143	1	Asp(50.0%), Ser(16.7%), Ala(16.7%), Gln(11.1%), Pro(5.6%)
144	1	Leu(61.1%), Gln(16.7%), Thr(5.6%), Lys(5.6%), Phe(5.6%), Ala(5.6%)
145	1	Leu(61.1%), Phe(22.2%), Met(11.1%), Ile(5.6%)
146	5	Lys(77.8%), Asn(11.1%), Ser(5.6%), Glu(5.6%)
147	1	Pro(38.9%), Thr(22.2%), Ser(16.7%), Glu(16.7%), Asp(5.6%)
148	4*	Ser(38.9%), Gly(38.9%), Asn(16.7%), Pro(5.6%)
149	3*	Gly(38.9%), Ser(27.8%), Ala(16.7%), Thr(11.1%), Cys(5.6%)
150	8	Thr(88.9%), Val(5.6%), Ile(5.6%)
151	8	Leu(94.4%), Val(5.6%)

152	4*	Ile(66.7%), Val(16.7%), Phe(11.1%), Met(5.6%)
153	8	Ser(88.9%), Gly(5.6%), Cys(5.6%)
154	6*	Phe(83.3%), Tyr(11.1%), His(5.6%)
155	5*	Ile(72.2%), Leu(22.2%), Val(5.6%)
156	1	Tyr(72.2%), Trp(11.1%), Gln(5.6%), His(5.6%), Gly(5.6%)
157	8	Pro(94.4%), Ala(5.6%)
158	3*	Ala(72.2%), Thr(11.1%), Leu(5.6%), His(5.6%), Gly(5.6%)
159	7	Gln(88.9%), Thr(5.6%), Lys(5.6%)
160	9	Asn(100.0%)
161	2*	Pro(72.2%), Lys(11.1%), Arg(5.6%), Gln(5.6%), Leu(5.6%)
162	1	Asp(50.0%), Glu(27.8%), Gln(11.1%), Pro(5.6%), Ala(5.6%)
163	8	Leu(94.4%), Val(5.6%)
164	2*	Leu(61.1%), Val(16.7%), Met(16.7%), Ile(5.6%)
165	1	Asp(33.3%), Asn(27.8%), Glu(16.7%), Ser(11.1%), Arg(5.6%), Gln(5.6%)
166	3*	Lys(72.2%), Ala(16.7%), Thr(5.6%), Ser(5.6%)
167	9	Leu(100.0%)
168	2*	Ser(50.0%), Ala(27.8%), Thr(11.1%), Gln(5.6%), Gly(5.6%)
169	1	Glu(38.9%), Gln(27.8%), Lys(16.7%), Ala(11.1%), Gly(5.6%)
170	2*	Arg(66.7%), Lys(22.2%), Thr(5.6%), Asn(5.6%)
171	2*	Lys(66.7%), Asn(22.2%), Arg(5.6%), Asp(5.6%)
172	1	Thr(33.3%), Met(22.2%), Ile(11.1%), Ala(11.1%), Val(5.6%), Ser(5.6%), Leu(5.6%), Lys(5.6%)
173	8	Thr(88.9%), Asn(11.1%)
174	5	Val(72.2%), Ala(22.2%), Ile(5.6%)
175	2*	Leu(50.0%), Phe(27.8%), Met(11.1%), Tyr(5.6%), Ile(5.6%)
176	9	Ala(100.0%)
177	9	Met(100.0%)
178	8	Asp(88.9%), Glu(11.1%)
179	3*	Gln(61.1%), Ser(11.1%), Leu(11.1%), Cys(11.1%), Ala(5.6%)
180	7	Val(83.3%), Ile(11.1%), Met(5.6%)
181	9	Pro(100.0%)
182	9	Arg(100.0%)

183	9	Val(61.1%), Ile(38.9%)
184	8	Thr(66.7%), Ser(33.3%)
185	8	Ile(61.1%), Arg(38.9%)
186	9	Ala(100.0%)
187	9	Gln(100.0%)
188	6	Gly(50.0%), Ser(22.2%), Val(16.7%), Ala(11.1%)
189	5	Tyr(50.0%), Phe(27.8%), Met(11.1%), Leu(11.1%)
190	9	Asp(100.0%)
191	7	Ala(88.9%), Val(5.6%), Ile(5.6%)
192	9	Leu(100.0%)
193	9	Ser(100.0%)
194	9	Ser(100.0%)
195	8	Met(88.9%), Gln(11.1%)
196	8	Ala(83.3%), Ser(16.7%)
197	9	Asn(94.4%), Thr(5.6%)
198	8	Ile(88.9%), Val(5.6%), Leu(5.6%)
199	7	Ala(77.8%), Ser(22.2%)
200	9	Gly(100.0%)
201	9	Tyr(100.0%)
202	5	Lys(66.7%), Arg(27.8%), Leu(5.6%)
203	9	Ala(100.0%)
204	6	Val(72.2%), Ile(22.2%), Ala(5.6%)
205	6	Val(72.2%), Ile(22.2%), Leu(5.6%)
206	3*	Leu(55.6%), Glu(27.8%), Met(5.6%), His(5.6%), Asp(5.6%)
207	8	Ala(94.4%), Gly(5.6%)
208	9	Ala(100.0%)
209	7	Asn(77.8%), His(11.1%), Tyr(5.6%), Arg(5.6%)
210	1	His(55.6%), Asn(22.2%), Glu(16.7%), Leu(5.6%)
211	8	Phe(94.4%), Ser(5.6%)
212	4*	Gly(77.8%), Pro(16.7%), Ala(5.6%)
213	9	Arg(100.0%)

214	7	Phe(94.4%), Ala(5.6%)
215	9	Phe(100.0%)
216	7	Thr(83.3%), Pro(11.1%), Ala(5.6%)
217	8	Gly(88.9%), Met(11.1%)
218	7	Gln(88.9%), Met(5.6%), Leu(5.6%)
219	8	Ile(88.9%), Thr(5.6%), Met(5.6%)
220	9	Thr(100.0%)
221	9	Ala(100.0%)
222	9	Ala(100.0%)
223	9	Gly(100.0%)
224	8	Lys(88.9%), Thr(11.1%)
225	9	Val(94.4%), Ile(5.6%)
226	8	Pro(94.4%), Arg(5.6%)
227	9	Pro(100.0%)
228	9	Ala(100.0%)
229	8	Lys(94.4%), Arg(5.6%)
230	8	Val(61.1%), Ile(38.9%)
231	7	Leu(83.3%), Met(16.7%)
232	9	Ile(61.1%), Val(38.9%)
233	6	Ile(61.1%), Val(27.8%), Met(5.6%), Phe(5.6%)
234	9	Gly(100.0%)
235	7	Gly(77.8%), Val(11.1%), Ala(11.1%)
236	9	Gly(100.0%)
237	9	Val(100.0%)
238	9	Ala(100.0%)
239	9	Gly(100.0%)
240	9	Leu(100.0%)
241	5*	Ala(77.8%), Ser(11.1%), Gln(5.6%), Met(5.6%)
242	8	Ala(61.1%), Ser(38.9%)
243	8	Ala(61.1%), Ile(38.9%)
244	8	Gly(88.9%), Ala(11.1%)

245	6	Ala(55.6%), Thr(33.3%), Ser(5.6%), Gln(5.6%)
246	9	Ala(94.4%), Ser(5.6%)
247	5*	Lys(72.2%), Arg(16.7%), Asn(11.1%)
248	3*	Ser(55.6%), Gly(22.2%), Arg(11.1%), Asn(5.6%), Ala(5.6%)
249	7	Met(72.2%), Leu(27.8%)
250	9	Gly(100.0%)
251	9	Ala(100.0%)
252	3*	Ile(66.7%), Val(27.8%), Gln(5.6%)
253	9	Val(100.0%)
254	6	Arg(88.9%), Met(5.6%), Phe(5.6%)
255	7	Gly(72.2%), Ala(27.8%)
256	5*	Phe(88.9%), Tyr(5.6%), Thr(5.6%)
257	9	Asp(100.0%)
258	8	Thr(88.9%), Val(11.1%)
259	9	Arg(100.0%)
260	4*	Ala(66.7%), Pro(16.7%), Asp(11.1%), Lys(5.6%)
261	7	Ala(77.8%), Glu(16.7%), Val(5.6%)
262	6	Ala(66.7%), Val(27.8%), Thr(5.6%)
263	6*	Leu(66.7%), Lys(33.3%)
264	9	Glu(100.0%)
265	8	Gln(94.4%), His(5.6%)
266	7	Phe(61.1%), Val(33.3%), Ala(5.6%)
267	5*	Lys(61.1%), Glu(22.2%), Gln(11.1%), Leu(5.6%)
268	9	Ser(100.0%)
269	5*	Leu(77.8%), Met(16.7%), Phe(5.6%)
270	9	Gly(100.0%)
271	8	Ala(94.4%), Gly(5.6%)
272	7	Glu(83.3%), Lys(11.1%), Gln(5.6%)
273	5*	Pro(66.7%), Phe(33.3%)
274	8	Leu(88.9%), Ile(11.1%)
275	2*	Glu(72.2%), Thr(16.7%), Lys(11.1%)

276	6	Val(77.8%), Leu(16.7%), Ile(5.6%)
277	5	Asp(77.8%), Asn(16.7%), Pro(5.6%)
278	3*	Leu(44.4%), Ile(33.3%), Phe(11.1%), Val(5.6%), Asp(5.6%)
279	4*	Lys(72.2%), Glu(22.2%), Ser(5.6%)
280	8	Glu(94.1%), Lys(5.9%)
281	3*	Ser(70.6%), Glu(11.8%), Asp(11.8%), Thr(5.9%)
282	8	Gly(77.8%), Ala(22.2%)
283	5	Glu(77.8%), Gly(11.1%), Ser(5.6%), Asp(5.6%)
284	4*	Gly(77.8%), Thr(11.1%), Ser(11.1%)
285	1	Gln(50.0%), Gly(22.2%), Ala(16.7%), Thr(5.6%), Glu(5.6%)
286	8	Gly(88.9%), Asp(11.1%)
287	9	Gly(100.0%)
288	9	Tyr(100.0%)
289	9	Ala(94.4%), Ser(5.6%)
290	8	Lys(94.4%), Arg(5.6%)
291	7	Glu(83.3%), Val(11.1%), Thr(5.6%)
292	8	Met(94.4%), Leu(5.6%)
293	8	Ser(88.9%), Thr(5.6%), Gly(5.6%)
294	7	Lys(77.8%), Glu(16.7%), Asp(5.6%)
295	8	Glu(88.9%), Ala(11.1%)
296	7	Phe(94.4%), Glu(5.6%)
297	7	Ile(88.9%), Arg(5.6%), Lys(5.6%)
298	5*	Glu(66.7%), Lys(16.7%), Asp(11.1%), Arg(5.6%)
299	7	Ala(88.9%), Lys(5.6%), Ile(5.6%)
300	8	Glu(88.9%), Gln(11.1%)
301	7	Met(88.9%), His(5.6%), Ala(5.6%)
302	1	Lys(50.0%), Ala(22.2%), Glu(16.7%), Ser(5.6%), Gln(5.6%)
303	8	Leu(88.9%), Ala(11.1%)
304	6*	Phe(88.9%), Val(5.6%), Leu(5.6%)
305	7	Ala(88.9%), Arg(5.6%), Leu(5.6%)
306	2*	Lys(50.0%), Gln(27.8%), Asp(11.1%), Ala(11.1%)

307	7	Gln(88.9%), His(5.6%), Glu(5.6%)
308	2*	Cys(72.2%), Ala(16.7%), Val(5.6%), Leu(5.6%)
309	2*	Lys(72.2%), Gln(11.1%), Val(5.6%), Leu(5.6%), Ala(5.6%)
310	2*	Glu(66.7%), Asp(22.2%), Lys(5.6%), Gly(5.6%)
311	6	Val(83.3%), Thr(5.6%), Met(5.6%), Ile(5.6%)
312	9	Asp(100.0%)
313	8	Ile(88.9%), Val(11.1%)
314	3*	Leu(44.4%), Ile(44.4%), Val(5.6%), Ala(5.6%)
315	7	Ile(72.2%), Val(27.8%)
316	9	Thr(66.7%), Ser(33.3%)
317	9	Thr(100.0%)
318	9	Ala(100.0%)
319	8	Leu(94.4%), Gln(5.6%)
320	9	Ile(94.4%), Val(5.6%)
321	9	Pro(100.0%)
322	9	Gly(100.0%)
323	6*	Lys(83.3%), Arg(16.7%)
324	2*	Lys(61.1%), Pro(33.3%), Arg(5.6%)
325	9	Ala(100.0%)
326	9	Pro(100.0%)
327	1	Val(38.9%), Lys(27.8%), Ile(27.8%), Thr(5.6%)
328	9	Leu(100.0%)
329	7	Ile(50.0%), Phe(44.4%), Leu(5.6%)
330	6	Thr(50.0%), Arg(22.2%), Asn(16.7%), Ser(11.1%)
331	4*	Lys(66.7%), Glu(22.2%), Arg(11.1%)
332	3*	Glu(66.7%), Asp(22.2%), Gln(11.1%)
333	9	Met(100.0%)
334	6	Ile(55.6%), Val(44.4%)
335	3*	Glu(72.2%), Lys(11.1%), Asp(11.1%), Thr(5.6%)
336	4*	Ser(72.2%), Leu(11.1%), Thr(5.6%), Arg(5.6%), Lys(5.6%)
337	8	Met(94.4%), Leu(5.6%)

338	9	Lys(100.0%)
339	4*	Glu(44.4%), Pro(27.8%), Asp(16.7%), Ser(5.6%), Ala(5.6%)
340	9	Gly(100.0%)
341	9	Ser(94.4%), Thr(5.6%)
342	9	Val(100.0%)
343	5	Val(72.2%), Ile(27.8%)
344	8	Val(94.4%), Ile(5.6%)
345	9	Asp(100.0%)
346	9	Leu(100.0%)
347	9	Ala(100.0%)
348	5	Ala(77.8%), Ser(16.7%), Val(5.6%)
349	8	Glu(88.9%), Gln(11.1%)
350	6	Ala(77.8%), Ser(11.1%), Asn(11.1%)
351	9	Gly(100.0%)
352	9	Gly(100.0%)
353	9	Asn(100.0%)
354	3*	Phe(33.3%), Cys(33.3%), Ile(27.8%), Val(5.6%)
355	1	Glu(72.2%), Ala(16.7%), Val(5.6%), Pro(5.6%)
356	5*	Thr(66.7%), Leu(22.2%), Tyr(11.1%)
357	8	Thr(88.9%), Ser(5.6%), Ile(5.6%)
358	1	Lys(66.7%), Val(22.2%), Arg(5.6%), Glu(5.6%)
359	9	Pro(100.0%)
360	9	Gly(100.0%)
361	4*	Glu(77.8%), Lys(16.7%), Asp(5.6%)
362	4*	Leu(66.7%), Val(22.2%), Ile(11.1%)
363	1	Tyr(66.7%), Val(16.7%), Ser(5.6%), Phe(5.6%), Glu(5.6%)
364	1	Val(50.0%), Ile(16.7%), Thr(11.1%), Lys(11.1%), Arg(5.6%), Glu(5.6%)
365	1	His(61.1%), Val(11.1%), Lys(11.1%), Tyr(5.6%), Glu(5.6%), Ala(5.6%)
366	3*	Lys(50.0%), Asn(16.7%), His(16.7%), Gly(11.1%), Arg(5.6%)
367	6*	Gly(83.3%), Asp(11.1%), Lys(5.6%)
368	7	Val(72.2%), Ile(27.8%)

369	3*	Thr(55.6%), Lys(16.7%), Val(11.1%), Ile(11.1%), Arg(5.6%)
370	5*	His(66.7%), Ile(22.2%), Val(11.1%)
371	4*	Ile(72.2%), Val(22.2%), Tyr(5.6%)
372	7	Gly(94.4%), Leu(5.6%)
373	2*	Tyr(77.8%), Pro(5.6%), Leu(5.6%), His(5.6%), Phe(5.6%)
374	8	Thr(94.4%), Leu(5.6%)
375	7	Asp(83.3%), Asn(11.1%), Glu(5.6%)
376	1	Leu(66.7%), Val(11.1%), Ile(11.1%), Phe(11.1%)
377	8	Pro(94.4%), Met(5.6%)
378	8	Ser(83.3%), Gly(11.1%), Cys(5.6%)
379	6	Arg(88.9%), Tyr(5.6%), Glu(5.6%)
380	5*	Leu(50.0%), Met(38.9%), Val(11.1%)
381	4*	Pro(44.4%), Ala(44.4%), Thr(5.6%), Ser(5.6%)
382	7	Thr(83.3%), Val(5.6%), Cys(5.6%), Ala(5.6%)
383	6	Gln(83.3%), His(11.1%), Asp(5.6%)
384	7	Ala(55.6%), Ser(44.4%)
385	9	Ser(94.4%), Leu(5.6%)
386	1	Thr(55.6%), Gln(11.1%), Glu(11.1%), Ser(5.6%), Pro(5.6%), Asn(5.6%), Phe(5.6%)
387	8	Leu(94.4%), Met(5.6%)
388	7	Tyr(94.4%), Phe(5.6%)
389	2*	Ser(55.6%), Ala(22.2%), Gly(16.7%), Glu(5.6%)
390	7	Asn(77.8%), Thr(11.1%), Lys(11.1%)
391	9	Asn(94.4%), Lys(5.6%)
392	6*	Ile(61.1%), Leu(22.2%), Met(11.1%), Glu(5.6%)
393	1	Thr(50.0%), Val(22.2%), Ser(11.1%), Tyr(5.6%), Leu(5.6%), Ala(5.6%)
394	7	Lys(72.2%), Asn(22.2%), Arg(5.6%)
395	3*	Leu(72.2%), Phe(22.2%), Ile(5.6%)
396	5*	Leu(72.2%), Ile(22.2%), Ser(5.6%)
397	1	Lys(66.7%), Leu(16.7%), Thr(5.6%), Ser(5.6%), Arg(5.6%)
398	2*	Ala(55.6%), Leu(16.7%), Ser(11.1%), Pro(5.6%), Asn(5.6%), His(5.6%)
399	3*	Ile(61.1%), Leu(22.2%), Met(5.6%), His(5.6%), Cys(5.6%)

400	3*	Ser(55.6%), Gly(16.7%), Cys(11.1%), Val(5.6%), Thr(5.6%), Ile(5.6%)
401	4*	Pro(71.4%), Lys(21.4%), Ser(7.1%)
402	5*	Asp(66.7%), Glu(27.8%), Asn(5.6%)
403	8	Lys(94.4%), Thr(5.6%)
404	2*	Asp(64.7%), Glu(23.5%), Ser(5.9%), Gly(5.9%)
405	3*	Asn(44.4%), Thr(27.8%), His(16.7%), Val(5.6%), Ala(5.6%)
406	1	Phe(66.7%), Tyr(11.1%), Ile(11.1%), Leu(5.6%), Ala(5.6%)
407	1	Tyr(38.9%), Asn(16.7%), Val(11.1%), Thr(5.6%), Met(5.6%), His(5.6%), Phe(5.6%), Asp(5.6%), Ala(5.6%)
408	1	Phe(50.0%), Val(16.7%), Ile(11.1%), Pro(5.6%), Met(5.6%), Leu(5.6%), Lys(5.6%)
409	1	Asp(33.3%), Glu(27.8%), Asn(22.2%), Lys(11.1%), Gln(5.6%)
410	1	Val(38.9%), Leu(22.2%), Phe(22.2%), Trp(5.6%), Ile(5.6%), Glu(5.6%)
411	7	Lys(61.1%), Glu(27.8%), Asp(11.1%)
412	7	Asp(88.9%), Thr(5.6%), Asn(5.6%)
413	3*	Asp(44.4%), Glu(38.9%), Val(11.1%), Thr(5.6%)
414	5*	Phe(90.9%), Leu(9.1%)
415	1	Asp(72.7%), Ser(9.1%), Ile(9.1%), Ala(9.1%)
416	1	Phe(63.6%), Tyr(27.3%), Ile(9.1%)
417	4*	Gly(90.9%), Gln(9.1%)
418	4*	Thr(72.7%), Asn(18.2%), Ser(9.1%)
419	1	Met(72.7%), Val(9.1%), Pro(9.1%), Ile(9.1%)
420	1	Gly(45.5%), Asp(36.4%), Tyr(9.1%), Ser(9.1%)
421	6*	His(90.9%), Asn(9.1%)
422	7	Val(83.3%), Ile(11.1%), Thr(5.6%)
423	4*	Val(50.0%), Ile(38.9%), Gln(5.6%), Ala(5.6%)
424	7	Arg(88.9%), Ser(5.6%), Cys(5.6%)
425	6*	Gly(88.9%), Lys(5.6%), Ala(5.6%)
426	4*	Thr(44.4%), Ser(27.8%), Val(11.1%), Ala(11.1%), Cys(5.6%)
427	1	Val(44.4%), Cys(16.7%), Thr(11.1%), Leu(11.1%), Ile(11.1%), Lys(5.6%)
428	7	Val(88.9%), Leu(5.6%), Ile(5.6%)
429	1	Met(50.0%), Val(22.2%), Leu(11.1%), Thr(5.6%), Gln(5.6%), Ile(5.6%)
430	3*	Lys(50.0%), Arg(22.2%), His(16.7%), Gln(11.1%)

431	3*	Asp(61.1%), Glu(27.8%), Asn(5.6%), Ala(5.6%)
432	9	Gly(100.0%)
433	2*	Lys(61.1%), Glu(16.7%), Gln(11.1%), Asn(5.6%), Ala(5.6%)
434	4*	Val(61.1%), Ile(22.2%), Leu(11.1%), Met(5.6%)
435	1	Ile(44.4%), Thr(22.2%), Met(11.1%), Leu(11.1%), Val(5.6%), Lys(5.6%)
436	5*	Phe(61.1%), Trp(27.8%), His(11.1%)
437	7	Pro(94.4%), Gly(5.6%)
438	5*	Ala(66.7%), Pro(22.2%), Ser(11.1%)
439	7	Pro(94.1%), Leu(5.9%)
440	1	Thr(35.3%), Pro(23.5%), Ala(17.6%), Val(11.8%), Gln(5.9%), Leu(5.9%)
441	1	Pro(70.6%), Ile(23.5%), Gly(5.9%)
442	2*	Lys(47.1%), Gln(35.3%), Asn(11.8%), Ser(5.9%)
443	3*	Asn(58.8%), Val(23.5%), Lys(5.9%), Gly(5.9%), Phe(5.9%)
444	1	Ile(52.9%), Ser(17.6%), Val(11.8%), Pro(5.9%), Asp(5.9%), Ala(5.9%)
445	5*	Pro(76.5%), Ala(17.6%), (5.9%)
446	1	Gln(64.7%), (5.9%), Val(5.9%), Pro(5.9%), Lys(5.9%), Glu(5.9%), Ala(5.9%)
447	1	Gly(35.3%), Pro(29.4%), Ala(23.5%), (5.9%), Glu(5.9%)
448	1	Ala(52.9%), Thr(17.6%), Gln(11.8%), Pro(11.8%), Met(5.9%)
449	6*	Pro(82.4%), Ala(11.8%), Glu(5.9%)
450	1*	Val(66.7%), Pro(16.7%), Asp(8.3%), Ala(8.3%)
451	1	Lys(66.7%), Pro(13.3%), Ala(13.3%), Val(6.7%)
452	1	Gln(56.3%), Pro(25.0%), Ala(12.5%), Asn(6.3%)
453	6*	Lys(82.4%), Ala(11.8%), Ile(5.9%)
454	1	Thr(41.2%), Ser(17.6%), Pro(17.6%), Ala(11.8%), Leu(5.9%), Lys(5.9%)
455	3*	Val(70.6%), Ala(17.6%), Thr(5.9%), Asp(5.9%)
456	1	Ala(56.3%), Glu(18.8%), Thr(6.3%), Gln(6.3%), Pro(6.3%), Lys(6.3%)
457	1	Glu(64.7%), Pro(11.8%), Lys(11.8%), Thr(5.9%), Gln(5.9%)
458	1	Leu(58.8%), Val(11.8%), Ala(11.8%), Ser(5.9%), Gln(5.9%), Ile(5.9%)
459	1	Glu(70.6%), Thr(5.9%), Ser(5.9%), Gln(5.9%), Pro(5.9%), Lys(5.9%)
460	1*	Ala(47.1%), Lys(23.5%), Val(11.8%), Thr(5.9%), Gln(5.9%), Asn(5.9%)
461	3*	Glu(73.3%), Val(13.3%), Thr(6.7%), Gln(6.7%)

462	5*	Lys(60.0%), Ala(26.7%), Glu(13.3%)
463	1	Ala(64.7%), Val(11.8%), Leu(11.8%), Pro(5.9%), Lys(5.9%)
464	1	Ala(41.2%), Glu(17.6%), Val(5.9%), Ser(5.9%), Gln(5.9%), Pro(5.9%), Lys(5.9%), Gly(5.9%), Cys(5.9%)
465	2*	Thr(47.1%), Pro(35.3%), Ser(5.9%), Leu(5.9%), Ala(5.9%)
466	1	Ile(41.2%), Val(11.8%), Pro(11.8%), Glu(11.8%), Ser(5.9%), Leu(5.9%), Cys(5.9%), Ala(5.9%)
467	2*	Thr(41.2%), Ser(41.2%), Asn(11.8%), Gln(5.9%)
468	2*	Pro(70.6%), Ala(11.8%), Tyr(5.9%), Met(5.9%), His(5.9%)
469	1	Phe(70.6%), Tyr(11.8%), Trp(11.8%), Ala(5.9%)
470	1*	Arg(58.8%), Gln(11.8%), Asn(11.8%), Ala(11.8%), Thr(5.9%)
471	1	Lys(64.7%), Ser(11.8%), Ala(11.8%), Met(5.9%), Asp(5.9%)
472	5*	Thr(70.6%), Tyr(11.8%), Lys(11.8%), Leu(5.9%)
473	1	Met(35.3%), Ala(23.5%), Leu(17.6%), Phe(17.6%), Val(5.9%)
474	1	Thr(35.3%), Lys(23.5%), Leu(17.6%), Ser(11.8%), Asn(11.8%)
475	1	Thr(35.3%), Ser(17.6%), Gln(11.8%), Met(11.8%), Glu(11.8%), Asn(5.9%), Gly(5.9%)
476	5	Ala(76.5%), Thr(11.8%), Ser(11.8%)
477	1	Ser(47.1%), Leu(17.6%), Ala(17.6%), Gly(11.8%), Met(5.9%)
478	1	Ala(41.2%), Val(23.5%), Leu(17.6%), Lys(5.9%), Ile(5.9%), His(5.9%)
479	2*	Tyr(76.5%), Ile(11.8%), Leu(5.9%), Ala(5.9%)
480	6	Thr(85.7%), Ser(14.3%)
481	1	Ala(57.1%), Gly(21.4%), Thr(14.3%), Ser(7.1%)
482	8	Gly(100.0%)
483	2*	Leu(80.0%), Val(6.7%), Thr(6.7%), Phe(6.7%)
484	1	Thr(46.7%), Gly(33.3%), Ala(13.3%), Lys(6.7%)
485	1	Gly(38.9%), Ser(22.2%), Thr(16.7%), Ile(11.1%), Val(5.6%), Ala(5.6%)
486	1	Leu(50.0%), Ile(27.8%), Met(11.1%), Val(5.6%), Ala(5.6%)
487	1	Leu(55.6%), Val(16.7%), Phe(11.1%), Ser(5.6%), Pro(5.6%), Ile(5.6%)
488	4*	Gly(83.3%), Ser(5.6%), Leu(5.6%), Ala(5.6%)
489	1	Leu(61.1%), Trp(11.1%), Met(11.1%), Gly(5.6%), Cys(5.6%), Ala(5.6%)
490	5*	Gly(77.8%), Ala(11.1%), Met(5.6%), Leu(5.6%)
491	1	Ile(44.4%), Ala(22.2%), Val(11.1%), Leu(11.1%), Met(5.6%), (5.6%)
492	1	Ala(38.9%), Val(11.1%), Leu(11.1%), Ile(11.1%), Thr(5.6%), Ser(5.6%), Asn(5.6%), Cys(5.6%), (5.6%)

493	2*	Ala(44.4%), Ser(27.8%), Val(11.1%), Gly(5.6%), Glu(5.6%), (5.6%)
494	3*	Pro(72.2%), Ala(11.1%), Thr(5.6%), Met(5.6%), Gly(5.6%)
495	2	Asn(55.6%), Thr(16.7%), Pro(11.1%), His(5.6%), Glu(5.6%), Ala(5.6%)
496	1	Pro(27.8%), Ser(22.2%), Leu(22.2%), Lys(11.1%), Val(5.6%), Phe(5.6%), Ala(5.6%)
497	2*	Ala(55.6%), Glu(16.7%), Ser(11.1%), Gln(11.1%), Asn(5.6%)
498	6	Phe(83.3%), Val(11.1%), Ile(5.6%)
499	4*	Thr(38.9%), Ser(38.9%), Trp(11.1%), Leu(11.1%)
500	3*	Gln(61.1%), Thr(11.1%), Ser(11.1%), Gly(11.1%), Trp(5.6%)
501	6*	Met(77.8%), His(11.1%), Leu(5.6%), Ala(5.6%)
502	1	Val(61.1%), Met(11.1%), Phe(11.1%), Ser(5.6%), Leu(5.6%), Ile(5.6%)
503	8	Thr(94.4%), Tyr(5.6%)
504	7	Thr(77.8%), Val(16.7%), Ile(5.6%)
505	7	Phe(88.9%), Cys(11.1%)
506	5*	Gly(66.7%), Ala(22.2%), Val(11.1%)
507	5*	Leu(83.3%), Val(5.6%), Met(5.6%), Ile(5.6%)
508	6	Ala(72.2%), Ser(22.2%), Thr(5.6%)
509	3*	Gly(66.7%), Cys(16.7%), Ile(11.1%), Ala(5.6%)
510	6	Ile(72.2%), Val(11.1%), Phe(11.1%), Leu(5.6%)
511	6	Val(83.3%), Ile(11.1%), Leu(5.6%)
512	9	Gly(100.0%)
513	7	Tyr(94.4%), Phe(5.6%)
514	5*	His(72.2%), Tyr(22.2%), Glu(5.6%)
515	7	Thr(77.8%), Val(16.7%), Leu(5.6%)
516	9	Val(94.4%), Ile(5.6%)
517	6*	Trp(94.4%), Thr(5.6%)
518	5*	Gly(77.8%), Asn(11.1%), Ser(5.6%), Arg(5.6%)
519	9	Val(100.0%)
520	6	Thr(72.2%), Ser(11.1%), Ala(11.1%), Pro(5.6%)
521	6*	Pro(83.3%), His(11.1%), Val(5.6%)
522	8	Ala(94.4%), Ile(5.6%)
523	9	Leu(100.0%)

524	9	His(100.0%)
525	8	Ser(83.3%), Thr(16.7%)
526	9	Pro(100.0%)
527	9	Leu(100.0%)
528	9	Met(100.0%)
529	9	Ser(94.4%), Gly(5.6%)
530	8	Val(94.4%), Gly(5.6%)
531	9	Thr(94.4%), Ser(5.6%)
532	9	Asn(100.0%)
533	8	Ala(94.4%), Phe(5.6%)
534	9	Ile(100.0%)
535	9	Ser(94.4%), His(5.6%)
536	8	Gly(94.4%), Ser(5.6%)
537	6*	Leu(61.1%), Ile(22.2%), Val(11.1%), Thr(5.6%)
538	8	Thr(77.8%), Ile(16.7%), Val(5.6%)
539	9	Ala(77.8%), Val(22.2%)
540	7	Val(77.8%), Ala(22.2%)
541	9	Gly(100.0%)
542	8	Gly(72.2%), Ala(27.8%)
543	8	Leu(94.4%), Met(5.6%)
544	1	Ala(38.9%), Leu(22.2%), Val(11.1%), Phe(11.1%), Ser(5.6%), Ile(5.6%), Cys(5.6%)
545	3*	Leu(72.2%), Gln(11.1%), Val(5.6%), Met(5.6%), Ala(5.6%)
546	6	Met(77.8%), Ile(11.1%), Thr(5.6%), Leu(5.6%)
547	9	Gly(100.0%)
548	4*	Gly(77.8%), His(11.1%), Gln(5.6%), Pro(5.6%)
549	1	Gly(38.9%), His(27.8%), Glu(16.7%), Asn(5.6%), Lys(5.6%), Ala(5.6%)
550	1	Tyr(43.8%), Leu(31.3%), Val(6.3%), Phe(6.3%), Glu(6.3%), Ala(6.3%)
551	1	Tyr(31.3%), Leu(31.3%), Phe(18.8%), Thr(12.5%), Met(6.3%)
552	8	Pro(100.0%)
553	1	Ser(64.3%), Thr(14.3%), Gln(7.1%), Asn(7.1%), Asp(7.1%)
554	2*	Thr(33.3%), Ser(27.8%), Asn(22.2%), Gly(16.7%)

555	2	Thr(61.1%), Trp(11.1%), Ala(11.1%), Ser(5.6%), Leu(5.6%), Ile(5.6%)
556	1	Ser(33.3%), Pro(16.7%), Ala(16.7%), Val(11.1%), Ile(11.1%), His(5.6%), Glu(5.6%)
557	2	Gln(55.6%), Glu(16.7%), Ser(11.1%), Lys(11.1%), His(5.6%)
558	1	Gly(38.9%), Thr(16.7%), Ser(16.7%), Leu(11.1%), Phe(11.1%), Val(5.6%)
559	6	Leu(88.9%), Met(5.6%), Ile(5.6%)
560	7	Ala(77.8%), Ser(11.1%), Gly(11.1%)
561	1	Ala(38.9%), Phe(22.2%), Val(16.7%), Leu(16.7%), Thr(5.6%)
562	1	Leu(61.1%), Ala(16.7%), Ile(11.1%), Ser(5.6%), Phe(5.6%)
563	8	Ala(83.3%), Ser(11.1%), Gly(5.6%)
564	3*	Ala(44.4%), Thr(33.3%), Val(16.7%), Ile(5.6%)
565	4*	Phe(72.2%), Leu(22.2%), Ile(5.6%)
566	6	Ile(77.8%), Val(11.1%), Leu(11.1%)
567	8	Ser(77.8%), Ala(16.7%), Gly(5.6%)
568	8	Ser(88.9%), Phe(5.6%), Ala(5.6%)
569	3*	Val(50.0%), Ile(44.4%), Ala(5.6%)
570	9	Asn(100.0%)
571	8	Ile(94.4%), Ala(5.6%)
572	1	Ala(55.6%), Phe(27.8%), Gly(11.1%), Val(5.6%)
573	9	Gly(100.0%)
574	9	Gly(100.0%)
575	8	Phe(94.4%), Tyr(5.6%)
576	3*	Leu(72.2%), Thr(11.1%), Val(5.6%), Ile(5.6%), Ala(5.6%)
577	9	Val(100.0%)
578	9	Thr(100.0%)
579	7	Gln(88.9%), Val(5.6%), Lys(5.6%)
580	9	Arg(100.0%)
581	9	Met(100.0%)
582	9	Leu(100.0%)
583	6*	Asp(77.8%), Lys(11.1%), Glu(5.6%), Ala(5.6%)
584	9	Met(100.0%)
585	9	Phe(100.0%)

586	7	Lys(83.3%), Arg(11.1%), Glu(5.6%)
587	7	Arg(83.3%), Lys(16.7%)
588	6*	Pro(75.0%), Lys(18.8%), Ala(6.3%)
589	2*	Thr(62.5%), Asp(25.0%), Gln(6.3%), Pro(6.3%)
590	7	Asp(93.3%), Lys(6.7%)
591	8	Pro(93.3%), (6.7%)
592	8	Pro(93.3%), (6.7%)
593	9	Glu(93.3%), (6.7%)
594	3*	Tyr(66.7%), His(26.7%), Met(6.7%)
595	8	Asn(93.3%), Thr(6.7%)
596	5*	Tyr(92.9%), Lys(7.1%)
597	8	Leu(100.0%)
598	6*	Tyr(92.9%), Phe(7.1%)
599	2*	Leu(64.3%), Gly(14.3%), Ala(14.3%), Ser(7.1%)
600	3*	Leu(50.0%), Ile(50.0%)
601	8	Pro(100.0%)
602	1	Ala(50.0%), Gly(28.6%), Thr(21.4%)
603	7	Gly(64.3%), Ala(35.7%)
604	2*	Thr(42.9%), Val(28.6%), Ala(21.4%), Leu(7.1%)
605	4*	Phe(78.6%), Tyr(14.3%), Leu(7.1%)
606	4*	Val(64.3%), Leu(28.6%), Ile(7.1%)
607	8	Gly(100.0%)
608	6*	Gly(85.7%), Ala(14.3%)
609	8	Tyr(100.0%)
610	1	Gly(40.0%), Leu(33.3%), Phe(13.3%), Tyr(6.7%), Ala(6.7%)
611	1	Ala(53.3%), Tyr(13.3%), Leu(13.3%), Val(6.7%), Gln(6.7%), Met(6.7%)
612	3*	Ala(58.8%), Gly(29.4%), Ser(5.9%), Asn(5.9%)
613	1	Leu(52.9%), Ala(11.8%), (11.8%), Val(5.9%), Thr(5.9%), Ser(5.9%), Gly(5.9%)
614	1	Tyr(41.2%), Gln(11.8%), Leu(11.8%), (11.8%), Ser(5.9%), Met(5.9%), His(5.9%), Gly(5.9%)
615	1	Ser(52.9%), Thr(11.8%), (11.8%), Arg(5.9%), Gln(5.9%), Asn(5.9%), Gly(5.9%)
616	4*	Gly(76.5%), Met(11.8%), (5.9%), Ala(5.9%)

617	1	Tyr(44.4%), Pro(16.7%), Ser(11.1%), Gly(11.1%), Met(5.6%), His(5.6%), Phe(5.6%)
618	1	Asn(50.0%), Asp(22.2%), Gly(11.1%), Ser(5.6%), Leu(5.6%), Glu(5.6%)
619	7	Ile(66.7%), Leu(33.3%)
620	4*	Glu(50.0%), His(27.8%), Val(11.1%), Thr(5.6%), Ile(5.6%)
621	4*	Gln(72.2%), Thr(11.1%), Ser(5.6%), Met(5.6%), Glu(5.6%)
622	6	Met(44.4%), Ile(27.8%), Ala(22.2%), Tyr(5.6%)
623	6	Met(55.6%), Ala(33.3%), Val(5.6%), Ile(5.6%)
624	9	Tyr(100.0%)
625	4*	Leu(66.7%), Ile(16.7%), Phe(16.7%)
626	5*	Gly(61.1%), Val(22.2%), Ile(11.1%), Ala(5.6%)
627	7	Ser(72.2%), Ala(22.2%), Val(5.6%)
628	4*	Gly(72.2%), Ala(16.7%), Ser(11.1%)
629	7	Leu(77.8%), Ile(16.7%), Val(5.6%)
630	6*	Cys(72.2%), Leu(22.2%), Ser(5.6%)
631	7	Cys(77.8%), Phe(22.2%)
632	8	Val(72.2%), Ile(27.8%)
633	4*	Gly(77.8%), Phe(11.1%), Val(5.6%), Leu(5.6%)
634	7	Ala(83.3%), Ser(11.1%), Gly(5.6%)
635	9	Leu(100.0%)
636	1	Ala(55.6%), Gly(22.2%), Val(5.6%), Thr(5.6%), Arg(5.6%), Lys(5.6%)
637	8	Gly(94.4%), Arg(5.6%)
638	8	Leu(94.4%), Met(5.6%)
639	9	Ser(94.4%), Ala(5.6%)
640	3*	Thr(44.4%), Ser(22.2%), Lys(22.2%), Asn(5.6%), His(5.6%)
641	7	Gln(77.8%), Pro(11.1%), His(11.1%)
642	2*	Gly(44.4%), Ser(22.2%), Glu(16.7%), Lys(11.1%), Thr(5.6%)
643	9	Thr(94.4%), Ser(5.6%)
644	8	Ala(83.3%), Ser(16.7%)
645	7	Arg(88.9%), Gln(5.6%), Lys(5.6%)
646	1	Leu(55.6%), Val(11.1%), Gln(11.1%), Met(11.1%), Ser(5.6%), Asn(5.6%)
647	9	Gly(100.0%)

648	8	Asn(94.4%), Ile(5.6%)
649	3*	Ala(72.2%), Val(11.1%), Tyr(5.6%), Arg(5.6%), Asn(5.6%)
650	4*	Leu(77.8%), Phe(11.1%), Trp(5.6%), Met(5.6%)
651	8	Gly(94.4%), Ala(5.6%)
652	3*	Met(61.1%), Ile(33.3%), Gly(5.6%)
653	4*	Ile(66.7%), Met(11.1%), Ala(11.1%), Trp(5.6%), Val(5.6%)
654	9	Gly(100.0%)
655	9	Val(77.8%), Met(22.2%)
656	4*	Ala(66.7%), Thr(11.1%), Ser(11.1%), Val(5.6%), Gly(5.6%)
657	4*	Gly(72.2%), Ile(16.7%), Ser(5.6%), Leu(5.6%)
658	8	Gly(77.8%), Ala(22.2%)
659	5	Leu(50.0%), Ile(38.9%), Val(11.1%)
660	1	Ala(55.6%), Val(11.1%), Leu(11.1%), Ile(11.1%), Thr(5.6%), Gly(5.6%)
661	9	Ala(94.4%), Thr(5.6%)
662	9	Thr(100.0%)
663	5*	Leu(77.8%), Ile(11.1%), Phe(11.1%)
664	8	Gly(100.0%)
665	1	Gly(28.6%), Val(21.4%), Cys(14.3%), Ser(7.1%), Leu(7.1%), Ile(7.1%), His(7.1%), Ala(7.1%)
666	1	Leu(66.7%), Ile(16.7%), Phe(11.1%), Met(5.6%)
667	1	Lys(55.6%), Ala(16.7%), Gly(11.1%), Trp(5.6%), Ser(5.6%), Gln(5.6%)
668	9	Pro(100.0%)
669	1	Ser(44.4%), Asp(27.8%), Gly(16.7%), Cys(11.1%)
670	1	Pro(50.0%), Thr(16.7%), Leu(11.1%), Val(5.6%), Met(5.6%), Phe(5.6%), Ala(5.6%)
671	1	Glu(50.0%), Gly(16.7%), Gln(11.1%), Ala(11.1%), Asn(5.6%), Asp(5.6%)
672	3*	Leu(61.1%), Asn(16.7%), Thr(11.1%), Val(5.6%), Ala(5.6%)
673	3*	Leu(72.2%), Val(11.1%), Tyr(5.6%), Met(5.6%), Phe(5.6%)
674	1	Ala(50.0%), Gly(22.2%), Thr(11.1%), Val(5.6%), Met(5.6%), Cys(5.6%)
675	6	Gln(77.8%), Trp(16.7%), Leu(5.6%)
676	8	Met(77.8%), Ile(22.2%)
677	1	Ser(55.6%), Val(11.1%), Leu(11.1%), Gly(11.1%), Ile(5.6%), Cys(5.6%)
678	1*	Gly(61.1%), Leu(22.2%), Thr(5.6%), Ser(5.6%), Ala(5.6%)

679	7	Ala(83.3%), Val(5.6%), Ser(5.6%), Cys(5.6%)
680	5	Met(77.8%), Val(5.6%), Leu(5.6%), Ile(5.6%), Ala(5.6%)
681	4*	Ala(66.7%), Gly(16.7%), Val(11.1%), Leu(5.6%)
682	1	Leu(50.0%), Ile(22.2%), Val(16.7%), Met(11.1%)
683	9	Gly(100.0%)
684	6	Gly(88.9%), Ser(11.1%)
685	5*	Thr(50.0%), Leu(22.2%), Ala(22.2%), Val(5.6%)
686	3*	Ile(66.7%), Leu(11.1%), Ala(11.1%), Val(5.6%), Thr(5.6%)
687	8	Gly(94.4%), Ala(5.6%)
688	1	Leu(61.1%), Thr(11.1%), Ile(11.1%), Trp(5.6%), Gly(5.6%), Phe(5.6%)
689	1	Thr(50.0%), Arg(11.1%), Gly(11.1%), Trp(5.6%), Val(5.6%), Leu(5.6%), Ile(5.6%), Ala(5.6%)
690	5	Ile(77.8%), Leu(11.1%), Ala(11.1%)
691	9	Ala(100.0%)
692	5*	Lys(83.3%), Val(5.6%), Arg(5.6%), Asn(5.6%)
693	5	Arg(66.7%), Lys(22.2%), Ser(11.1%)
694	8	Ile(77.8%), Val(22.2%)
695	1	Gln(55.6%), Glu(27.8%), Leu(5.6%), Lys(5.6%), Ala(5.6%)
696	7	Ile(66.7%), Met(22.2%), Val(11.1%)
697	8	Thr(55.6%), Ser(44.4%)
698	4*	Asp(72.2%), Glu(11.1%), Thr(5.6%), Ser(5.6%), Ala(5.6%)
699	7	Leu(83.3%), Met(16.7%)
700	9	Pro(100.0%)
701	8	Gln(88.9%), Glu(11.1%)
702	6	Leu(88.9%), Met(11.1%)
703	9	Val(100.0%)
704	9	Ala(100.0%)
705	5	Ala(77.8%), Ile(16.7%), Leu(5.6%)
706	6	Phe(83.3%), Leu(11.1%), Tyr(5.6%)
707	8	His(94.4%), Asn(5.6%)
708	9	Ser(94.4%), Gly(5.6%)
709	5*	Leu(77.8%), Phe(16.7%), Met(5.6%)

710	8	Val(94.4%), Gly(5.6%)
711	9	Gly(100.0%)
712	3*	Leu(77.8%), Ala(11.1%), Met(5.6%), Gly(5.6%)
713	9	Ala(100.0%)
714	9	Ala(100.0%)
715	7	Val(88.9%), Thr(5.6%), Ala(5.6%)
716	6*	Leu(83.3%), Met(11.1%), Thr(5.6%)
717	7	Thr(77.8%), Val(16.7%), Ile(5.6%)
718	6	Cys(77.8%), Gly(11.1%), Ala(11.1%)
719	1	Val(33.3%), Ile(22.2%), Phe(16.7%), Met(11.1%), Thr(5.6%), Leu(5.6%), Ala(5.6%)
720	5*	Ala(72.2%), Asn(11.1%), Val(5.6%), Ser(5.6%), Gly(5.6%)
721	3*	Glu(66.7%), Ser(11.1%), Asn(11.1%), Thr(5.6%), Ala(5.6%)
722	1*	Tyr(66.7%), Phe(16.7%), Leu(11.1%), His(5.6%)
723	5*	Met(38.9%), Ile(33.3%), Leu(27.8%)
724	1	Ile(38.9%), Val(22.2%), Gln(11.1%), His(11.1%), Asn(5.6%), Leu(5.6%), Lys(5.6%)
725	3*	Glu(72.2%), His(11.1%), Pro(5.6%), Gly(5.6%), Asp(5.6%)
726	1	Tyr(50.0%), His(16.7%), Glu(11.1%), Gln(5.6%), Phe(5.6%), Asp(5.6%), Ala(5.6%)
727	4*	Pro(77.8%), Ala(11.1%), Thr(5.6%), Phe(5.6%)
728	5*	His(81.3%), Tyr(6.3%), Thr(6.3%), Glu(6.3%)
729	3*	Phe(64.7%), Leu(17.6%), Gly(17.6%)
730	5	Ala(76.5%), Met(11.8%), Ser(5.9%), Leu(5.9%)
731	1	Thr(52.9%), Glu(23.5%), Ala(11.8%), Met(5.9%), Leu(5.9%)
732	1	Asp(64.7%), Asn(17.6%), Gln(5.9%), Pro(5.9%), Gly(5.9%)
733	1	Pro(35.3%), Ala(35.3%), Glu(17.6%), Ile(11.8%)
734	2*	Ala(58.8%), Met(11.8%), Leu(11.8%), Thr(5.9%), Ser(5.9%), Ile(5.9%)
735	3*	Ala(61.1%), Val(11.1%), Gly(11.1%), Ser(5.6%), Asn(5.6%), His(5.6%)
736	5*	Asn(77.8%), Ala(11.1%), Thr(5.6%), Gly(5.6%)
737	1	Leu(44.4%), Val(11.1%), Met(11.1%), Ile(11.1%), Gly(11.1%), Phe(5.6%), Ala(5.6%)
738	1	Thr(50.0%), Leu(16.7%), Ile(11.1%), His(11.1%), Ser(5.6%), Ala(5.6%)
739	6	Lys(77.8%), Leu(16.7%), Met(5.6%)
740	1	Ile(50.0%), Thr(16.7%), Phe(11.1%), Val(5.6%), Leu(5.6%), Gly(5.6%), Ala(5.6%)

741	5	Val(61.1%), Glu(16.7%), Ala(16.7%), Leu(5.6%)
742	4*	Ala(66.7%), Leu(16.7%), Val(11.1%), Met(5.6%)
743	4*	Tyr(61.1%), Phe(27.8%), Ser(5.6%), Ile(5.6%)
744	7	Leu(94.4%), Ala(5.6%)
745	9	Gly(100.0%)
746	6	Thr(77.8%), Ile(11.1%), Leu(5.6%), Gly(5.6%)
747	2*	Tyr(72.2%), Phe(16.7%), Leu(5.6%), Ala(5.6%)
748	9	Ile(94.4%), Val(5.6%)
749	9	Gly(100.0%)
750	7	Gly(77.8%), Ala(16.7%), Ser(5.6%)
751	8	Val(94.4%), Ile(5.6%)
752	9	Thr(94.4%), Ala(5.6%)
753	9	Phe(100.0%)
754	7	Ser(61.1%), Thr(38.9%)
755	9	Gly(100.0%)
756	9	Ser(100.0%)
757	6	Leu(83.3%), Val(16.7%)
758	4*	Val(61.1%), Ile(33.3%), Met(5.6%)
759	9	Ala(100.0%)
760	6*	Tyr(72.2%), Phe(27.8%)
761	8	Gly(94.4%), Ala(5.6%)
762	9	Lys(100.0%)
763	9	Leu(100.0%)
764	7	Gln(88.9%), Arg(5.6%), Cys(5.6%)
765	9	Gly(100.0%)
766	1	Ile(55.6%), Leu(27.8%), Lys(11.1%), Val(5.6%)
767	6	Leu(77.8%), Ile(16.7%), Met(5.6%)
768	1	Lys(38.9%), Asn(27.8%), Ser(11.1%), Asp(11.1%), Ala(11.1%)
769	9	Ser(94.4%), Gly(5.6%)
770	3*	Ala(66.7%), Arg(11.1%), Lys(11.1%), Ser(5.6%), Asp(5.6%)
771	8	Pro(88.9%), Ala(11.1%)

772	3*	Leu(72.2%), Val(16.7%), Thr(5.6%), Ile(5.6%)
773	1	Leu(61.1%), Met(27.8%), Tyr(5.6%), Thr(5.6%)
774	7	Leu(88.9%), Phe(11.1%)
775	9	Pro(100.0%)
776	3*	Gly(77.8%), Asn(11.1%), Met(5.6%), Ala(5.6%)
777	8	Arg(88.9%), Gln(11.1%)
778	7	His(88.9%), Asn(5.6%), Lys(5.6%)
779	1	Leu(33.3%), Met(22.2%), Ala(22.2%), Lys(11.1%), Val(5.6%), Pro(5.6%)
780	3*	Leu(77.8%), Met(11.1%), Val(5.6%), Ile(5.6%)
781	9	Asn(100.0%)
782	6	Ala(77.8%), Leu(16.7%), Gly(5.6%)
783	2*	Gly(66.7%), Ala(16.7%), Val(5.6%), Thr(5.6%), Leu(5.6%)
784	4*	Leu(77.8%), Ala(11.1%), Val(5.6%), Met(5.6%)
785	3*	Leu(83.3%), Gly(11.1%), Met(5.6%)
786	1	Ala(55.6%), Thr(22.2%), Val(11.1%), Leu(5.6%), Ile(5.6%)
787	1*	Ala(44.4%), Gly(27.8%), Val(11.1%), Leu(11.1%), Ser(5.6%)
788	6	Ser(77.8%), Asn(11.1%), Thr(5.6%), Leu(5.6%)
789	1	Val(55.6%), Phe(22.2%), Leu(11.1%), Ala(11.1%)
790	1	Gly(61.1%), Leu(16.7%), Val(11.1%), Ile(5.6%), Ala(5.6%)
791	5*	Gly(50.0%), Leu(22.2%), Ala(16.7%), Pro(11.1%)
792	1	Ile(38.9%), Met(33.3%), Leu(22.2%), Gly(5.6%)
793	1	Ile(66.7%), Val(11.1%), Gly(11.1%), Leu(5.6%), Ala(5.6%)
794	1*	Pro(61.1%), Val(11.1%), Thr(11.1%), Ser(5.6%), Met(5.6%), Ile(5.6%)
795	1	Phe(61.1%), Tyr(33.3%), Leu(5.6%)
796	3*	Met(66.7%), Val(11.1%), Tyr(5.6%), Leu(5.6%), Ile(5.6%), Ala(5.6%)
797	1	Leu(22.2%), Val(16.7%), Met(16.7%), Arg(11.1%), Ala(11.1%), Tyr(5.6%), Trp(5.6%), Lys(5.6%), Ile(5.6%)
798	1	Asp(55.6%), Ser(16.7%), Thr(11.1%), Asn(5.6%), Lys(5.6%), Glu(5.6%)
799	1	Pro(55.6%), Asp(16.7%), Asn(11.1%), Glu(11.1%), Thr(5.6%)
800	5	Ser(76.5%), Asp(11.8%), Thr(5.9%), Ala(5.9%)
801	1	Phe(41.2%), Tyr(23.5%), Thr(11.8%), Val(5.9%), Pro(5.9%), Met(5.9%), His(5.9%)
802	1	Thr(52.9%), Gly(17.6%), Ala(17.6%), Ser(5.9%), Asn(5.9%)

803	4*	Thr(70.6%), Ser(5.9%), Met(5.9%), Leu(5.9%), Gly(5.9%), Ala(5.9%)
804	1	Gly(70.6%), Gln(11.8%), Tyr(5.9%), Leu(5.9%), Ile(5.9%)
805	1	Ile(47.1%), Leu(23.5%), Val(17.6%), Met(5.9%), Phe(5.9%)
806	1	Thr(47.1%), Leu(17.6%), Met(11.8%), Gly(11.8%), Ser(5.9%), Ala(5.9%)
807	1	Cys(64.7%), Met(11.8%), Leu(11.8%), Phe(5.9%), Ala(5.9%)
808	6	Leu(88.2%), Met(5.9%), Phe(5.9%)
809	6*	Gly(86.7%), Leu(13.3%)
810	1	Ser(66.7%), Val(13.3%), Thr(6.7%), Ile(6.7%), Gly(6.7%)
811	7	Val(60.0%), Thr(26.7%), Met(13.3%)
812	4*	Ser(60.0%), Thr(20.0%), Ala(13.3%), Val(6.7%)
813	1	Ala(64.7%), Leu(11.8%), Gly(11.8%), Thr(5.9%), Ile(5.9%)
814	8	Leu(88.2%), Ile(11.8%)
815	9	Ser(76.5%), Ala(23.5%)
816	1	Ala(47.1%), Thr(17.6%), Leu(17.6%), Ser(11.8%), Phe(5.9%)
817	1	Val(47.1%), Leu(17.6%), Ile(17.6%), Ala(17.6%)
818	5	Met(70.6%), Phe(17.6%), Leu(11.8%)
819	9	Gly(100.0%)
820	4*	Val(66.7%), Trp(11.1%), Thr(11.1%), Leu(5.6%), Phe(5.6%)
821	7	Thr(77.8%), Leu(11.1%), His(11.1%)
822	8	Leu(94.4%), Met(5.6%)
823	7	Thr(83.3%), Val(11.1%), Ile(5.6%)
824	3*	Ala(72.2%), Met(11.1%), Val(5.6%), Leu(5.6%), Ile(5.6%)
825	7	Ala(77.8%), Ser(11.1%), Pro(11.1%)
826	9	Ile(100.0%)
827	9	Gly(100.0%)
828	9	Gly(100.0%)
829	8	Ala(94.4%), Gly(5.6%)
830	9	Asp(100.0%)
831	9	Met(100.0%)
832	9	Pro(100.0%)
833	9	Val(94.4%), Ile(5.6%)

834	9	Val(94.4%), Ala(5.6%)
835	9	Ile(88.9%), Val(11.1%)
836	9	Thr(77.8%), Ser(22.2%)
837	7	Val(77.8%), Met(16.7%), Phe(5.6%)
838	7	Leu(94.4%), Tyr(5.6%)
839	9	Asn(100.0%)
840	9	Ser(94.4%), Ala(5.6%)
841	7	Tyr(94.4%), Phe(5.6%)
842	9	Ser(94.4%), Thr(5.6%)
843	9	Gly(100.0%)
844	6*	Trp(94.4%), Met(5.6%)
845	9	Ala(100.0%)
846	6	Leu(77.8%), Ala(16.7%), Val(5.6%)
847	6	Cys(77.8%), Ala(16.7%), Gly(5.6%)
848	7	Ala(88.9%), Gly(5.6%), Phe(5.6%)
849	6	Glu(83.3%), Ala(11.1%), Ile(5.6%)
850	9	Gly(100.0%)
851	9	Phe(100.0%)
852	6	Leu(61.1%), Met(27.8%), Thr(5.6%), Ala(5.6%)
853	8	Leu(94.4%), Val(5.6%)
854	6	Asn(72.2%), Ser(11.1%), Gly(11.1%), Asp(5.6%)
855	9	Asn(100.0%)
856	6*	Asn(72.2%), Pro(11.1%), Asp(11.1%), Ser(5.6%)
857	7	Leu(94.4%), Ala(5.6%)
858	8	Leu(88.9%), Met(11.1%)
859	8	Thr(77.8%), Ile(16.7%), Met(5.6%)
860	7	Ile(77.8%), Val(22.2%)
861	6	Val(72.2%), Thr(11.1%), Ala(11.1%), Leu(5.6%)
862	9	Gly(100.0%)
863	9	Ala(94.4%), Thr(5.6%)
864	8	Leu(94.4%), Met(5.6%)

865	8	Ile(77.8%), Val(22.2%)
866	9	Gly(100.0%)
867	9	Ser(94.4%), Ala(5.6%)
868	9	Ser(94.4%), Ala(5.6%)
869	9	Gly(100.0%)
870	9	Ala(94.4%), Thr(5.6%)
871	8	Ile(94.4%), Leu(5.6%)
872	9	Leu(100.0%)
873	9	Ser(94.4%), Thr(5.6%)
874	5*	Tyr(88.9%), Val(5.6%), His(5.6%)
875	8	Ile(94.4%), Leu(5.6%)
876	9	Met(100.0%)
877	7	Cys(94.4%), Ala(5.6%)
878	6*	Val(66.7%), Lys(27.8%), Arg(5.6%)
879	8	Ala(94.4%), Gly(5.6%)
880	9	Met(100.0%)
881	9	Asn(100.0%)
882	9	Arg(100.0%)
883	9	Ser(100.0%)
884	4*	Leu(72.2%), Ile(11.1%), Phe(11.1%), Val(5.6%)
885	1	Ala(55.6%), Met(11.1%), Ile(11.1%), Phe(11.1%), Trp(5.6%), Leu(5.6%)
886	7	Asn(77.8%), Ser(22.2%)
887	9	Val(100.0%)
888	8	Ile(94.4%), Leu(5.6%)
889	6*	Leu(83.3%), Ala(11.1%), Val(5.6%)
890	9	Gly(100.0%)
891	9	Gly(100.0%)
892	6*	Tyr(72.2%), Phe(22.2%), Val(5.6%)
893	9	Gly(100.0%)
894	7	Thr(88.9%), Val(5.6%), Ser(5.6%)
895	5*	Thr(44.4%), Ser(27.8%), Glu(11.1%), Asp(11.1%), Lys(5.6%)

896	7	Ser(77.8%), Gly(16.7%), Gln(5.6%)
897	5*	Thr(72.2%), Ser(11.1%), Lys(5.6%), Gly(5.6%), Glu(5.6%)
898	4*	Ala(50.0%), Gly(33.3%), Ser(11.1%), Val(5.6%)
899	1	Gly(61.1%), Thr(16.7%), Ser(11.1%), Lys(5.6%), Ala(5.6%)
900	7	Gly(94.1%), Ala(5.9%)
901	3*	Lys(76.5%), Gly(5.9%), Glu(5.9%), Asp(5.9%), Ala(5.9%)
902	4*	Pro(76.5%), Asp(11.8%), Gly(5.9%), Ala(5.9%)
903	1	Met(70.6%), Gln(5.9%), Lys(5.9%), Gly(5.9%), Glu(5.9%), Ala(5.9%)
904	7	Glu(88.9%), Ala(11.1%)
905	7	Ile(77.8%), Val(16.7%), Ala(5.6%)
906	3*	Thr(46.7%), Ser(33.3%), Val(6.7%), Lys(6.7%), Glu(6.7%)
907	9	Gly(100.0%)
908	7	Thr(77.8%), Ser(11.1%), Glu(11.1%)
909	5	His(83.3%), Val(5.6%), Leu(5.6%), Ala(5.6%)
910	7	Thr(72.2%), Lys(16.7%), Arg(11.1%)
911	7	Glu(88.9%), Pro(5.6%), Ala(5.6%)
912	3*	Ile(61.1%), Val(33.3%), Gly(5.6%)
913	4*	Asn(66.7%), Thr(11.1%), Ser(11.1%), Asp(5.6%), Ala(5.6%)
914	4*	Val(38.9%), Leu(38.9%), Ala(16.7%), Pro(5.6%)
915	6*	Asp(66.7%), Glu(27.8%), Val(5.6%)
916	2*	Asn(38.9%), Asp(27.8%), Glu(16.7%), Ser(5.6%), Gln(5.6%), Gly(5.6%)
917	5	Ala(61.1%), Thr(33.3%), Val(5.6%)
918	5*	Val(38.9%), Ala(27.8%), Ile(22.2%), Thr(11.1%)
919	1	Glu(38.9%), Asp(38.9%), Gln(11.1%), Val(5.6%), Phe(5.6%)
920	5	Met(77.8%), Leu(11.1%), Tyr(5.6%), Ile(5.6%)
921	7	Ile(72.2%), Leu(22.2%), Met(5.6%)
922	2*	Lys(50.0%), Arg(33.3%), Asn(5.6%), Leu(5.6%), Ala(5.6%)
923	4*	Glu(55.6%), Asn(27.8%), Asp(11.1%), Tyr(5.6%)
924	8	Ala(83.3%), Ser(16.7%)
925	1	Asn(44.4%), His(16.7%), Lys(11.1%), Glu(11.1%), Ser(5.6%), Arg(5.6%), Gly(5.6%)
926	6	Ser(44.4%), Asn(44.4%), Lys(11.1%)

927	8	Ile(72.2%), Val(27.8%)
928	8	Ile(88.9%), Val(11.1%)
929	8	Ile(94.4%), Phe(5.6%)
930	5*	Thr(61.1%), Val(33.3%), Ile(5.6%)
931	9	Pro(100.0%)
932	9	Gly(100.0%)
933	9	Tyr(100.0%)
934	9	Gly(100.0%)
935	8	Leu(77.8%), Met(22.2%)
936	8	Cys(77.8%), Ala(22.2%)
937	6	Ala(55.6%), Val(38.9%), Leu(5.6%)
938	9	Ala(94.4%), Ser(5.6%)
939	8	Lys(72.2%), Gln(27.8%)
940	9	Ala(100.0%)
941	9	Gln(100.0%)
942	8	Tyr(88.9%), His(11.1%)
943	6*	Pro(88.9%), Lys(5.6%), Ala(5.6%)
944	6	Ile(72.2%), Val(16.7%), Leu(11.1%)
945	7	Ala(88.9%), Arg(5.6%), Lys(5.6%)
946	7	Asp(61.1%), Glu(33.3%), Gln(5.6%)
947	5	Leu(61.1%), Met(27.8%), Ile(11.1%)
948	8	Val(77.8%), Thr(11.1%), Ala(11.1%)
949	4*	Lys(55.6%), Asn(16.7%), Glu(11.1%), Asp(11.1%), Ser(5.6%)
950	1	Met(44.4%), Lys(16.7%), Val(5.6%), Thr(5.6%), Ser(5.6%), Arg(5.6%), Leu(5.6%), Ile(5.6%), Glu(5.6%)
951	9	Leu(100.0%)
952	1	Thr(33.3%), Arg(22.2%), Lys(16.7%), Val(5.6%), Ser(5.6%), Gln(5.6%), Glu(5.6%), Cys(5.6%)
953	1	Glu(44.4%), Asp(16.7%), Ala(16.7%), Lys(11.1%), Ser(5.6%), Gln(5.6%)
954	1	Gln(44.4%), Arg(22.2%), Glu(11.1%), Ala(11.1%), Lys(5.6%), His(5.6%)
955	9	Gly(100.0%)
956	5*	Lys(66.7%), Val(16.7%), Ile(11.1%), His(5.6%)
957	1	Lys(50.0%), Asn(27.8%), Glu(11.1%), Arg(5.6%), His(5.6%)

958	9	Val(100.0%)
959	7	Arg(88.9%), Ser(5.6%), Lys(5.6%)
960	7	Phe(94.4%), Tyr(5.6%)
961	6	Gly(83.3%), Ala(16.7%)
962	9	Ile(100.0%)
963	9	His(100.0%)
964	9	Pro(100.0%)
965	9	Val(100.0%)
966	9	Ala(100.0%)
967	9	Gly(100.0%)
968	9	Arg(100.0%)
969	8	Met(88.9%), Leu(11.1%)
970	9	Pro(100.0%)
971	9	Gly(100.0%)
972	8	Gln(77.8%), His(22.2%)
973	8	Leu(77.8%), Met(22.2%)
974	9	Asn(100.0%)
975	9	Val(100.0%)
976	9	Leu(100.0%)
977	9	Leu(100.0%)
978	9	Ala(100.0%)
979	9	Glu(100.0%)
980	9	Ala(100.0%)
981	5*	Gly(83.3%), Lys(11.1%), Asn(5.6%)
982	9	Val(100.0%)
983	7	Pro(94.4%), Asp(5.6%)
984	9	Tyr(100.0%)
985	9	Asp(100.0%)
986	2*	Ile(66.7%), Val(16.7%), Lys(5.6%), Glu(5.6%), Asp(5.6%)
987	8	Val(94.4%), Leu(5.6%)
988	1	Leu(77.8%), Gln(5.6%), Lys(5.6%), Phe(5.6%), Glu(5.6%)

989	8	Glu(94.4%), Asp(5.6%)
990	8	Met(88.9%), Leu(11.1%)
991	6*	Asp(77.8%), Glu(22.2%)
992	9	Glu(100.0%)
993	9	Ile(94.4%), Leu(5.6%)
994	9	Asn(100.0%)
995	1	His(33.3%), Glu(27.8%), Asp(22.2%), Ser(11.1%), Pro(5.6%)
996	7	Asp(88.9%), Ser(5.6%), Glu(5.6%)
997	9	Phe(100.0%)
998	1	Pro(61.1%), Ser(16.7%), Thr(5.6%), Gln(5.6%), Lys(5.6%), Ala(5.6%)
999	1	Asp(61.1%), Glu(16.7%), Thr(11.1%), Gln(5.6%), Ala(5.6%)
1000	8	Thr(88.9%), Val(5.6%), Ala(5.6%)
1001	9	Asp(100.0%)
1002	6*	Leu(72.2%), Val(16.7%), Thr(11.1%)
1003	6	Val(77.8%), Ala(22.2%)
1004	6	Leu(88.9%), Val(5.6%), Phe(5.6%)
1005	9	Val(100.0%)
1006	9	Ile(88.9%), Val(11.1%)
1007	9	Gly(100.0%)
1008	9	Ala(94.4%), Ser(5.6%)
1009	9	Asn(100.0%)
1010	9	Asp(100.0%)
1011	8	Thr(88.9%), Val(11.1%)
1012	7	Val(88.9%), Thr(5.6%), Ile(5.6%)
1013	9	Asn(100.0%)
1014	8	Ser(77.8%), Pro(22.2%)
1015	9	Ala(100.0%)
1016	9	Ala(100.0%)
1017	5*	Gln(72.2%), Glu(16.7%), Arg(5.6%), Lys(5.6%)
1018	4*	Glu(70.6%), Asp(23.5%), Thr(5.9%)
1019	8	Asp(94.4%), Arg(5.6%)

1020	9	Pro(100.0%)
1021	6	Asn(77.8%), Lys(11.1%), Ser(5.6%), Gly(5.6%)
1022	9	Ser(100.0%)
1023	7	Ile(72.2%), Pro(22.2%), Ser(5.6%)
1024	8	Ile(94.4%), Leu(5.6%)
1025	8	Ala(88.9%), Tyr(11.1%)
1026	9	Gly(100.0%)
1027	9	Met(100.0%)
1028	9	Pro(100.0%)
1029	8	Val(88.9%), Ile(11.1%)
1030	9	Leu(100.0%)
1031	5*	Glu(72.2%), Arg(16.7%), Asp(11.1%)
1032	9	Val(100.0%)
1033	5*	Trp(88.9%), Glu(5.6%), Asp(5.6%)
1034	7	Lys(88.9%), Asn(5.6%), Leu(5.6%)
1035	7	Ser(61.1%), Ala(38.9%)
1036	3*	Lys(72.2%), Gln(11.1%), Asn(5.6%), Gly(5.6%), Asp(5.6%)
1037	7	Gln(77.8%), Asn(16.7%), Thr(5.6%)
1038	9	Val(100.0%)
1039	4*	Ile(72.2%), Val(22.2%), Leu(5.6%)
1040	7	Val(88.9%), Ile(5.6%), Phe(5.6%)
1041	6	Met(72.2%), Ile(11.1%), Phe(11.1%), Val(5.6%)
1042	9	Lys(100.0%)
1043	9	Arg(100.0%)
1044	7	Ser(83.3%), Thr(11.1%), Gly(5.6%)
1045	6	Leu(77.8%), Met(16.7%), Gln(5.6%)
1046	6*	Gly(83.3%), Asn(11.1%), Ala(5.6%)
1047	6	Val(72.2%), Thr(16.7%), Ser(5.6%), Lys(5.6%)
1048	9	Gly(100.0%)
1049	7	Tyr(94.4%), Phe(5.6%)
1050	9	Ala(100.0%)

1051	8	Ala(77.8%), Gly(22.2%)
1052	9	Val(100.0%)
1053	7	Asp(77.8%), Gln(11.1%), Glu(11.1%)
1054	9	Asn(100.0%)
1055	8	Pro(88.9%), Glu(11.1%)
1056	6*	Ile(61.1%), Leu(22.2%), Val(16.7%)
1057	9	Phe(100.0%)
1058	4*	Tyr(72.2%), Phe(27.8%)
1059	5	Lys(83.3%), Arg(5.6%), Asn(5.6%), Ala(5.6%)
1060	2*	Pro(61.1%), Glu(27.8%), Gln(5.6%), Asn(5.6%)
1061	9	Asn(100.0%)
1062	9	Thr(100.0%)
1063	1	Ala(50.0%), Ser(22.2%), His(11.1%), Arg(5.6%), Gln(5.6%), Met(5.6%)
1064	9	Met(100.0%)
1065	9	Leu(100.0%)
1066	6	Leu(77.8%), Phe(16.7%), Tyr(5.6%)
1067	9	Gly(100.0%)
1068	9	Asp(100.0%)
1069	9	Ala(100.0%)
1070	8	Lys(94.4%), Gln(5.6%)
1071	8	Lys(88.9%), Ala(11.1%)
1072	6	Thr(72.2%), Ser(11.1%), Met(11.1%), Val(5.6%)
1073	2*	Cys(72.2%), Val(11.1%), Thr(5.6%), Ser(5.6%), Leu(5.6%)
1074	6*	Asp(83.3%), Glu(11.1%), Thr(5.6%)
1075	5*	Ala(77.8%), Ser(5.6%), Gln(5.6%), Lys(5.6%), Glu(5.6%)
1076	6	Leu(83.3%), Ile(16.7%)
1077	4*	Gln(55.6%), Leu(27.8%), Val(5.6%), Ser(5.6%), Ile(5.6%)
1078	2*	Ala(55.6%), Ser(11.1%), Gln(11.1%), Lys(11.1%), Thr(5.6%), Glu(5.6%)
1079	5*	Lys(64.7%), Ala(23.5%), Ser(5.9%), Glu(5.9%)
1080	2*	Val(52.9%), Leu(23.5%), Ile(17.6%), Met(5.9%)
1081	5	Arg(60.0%), Lys(26.7%), Ser(6.7%), Asn(6.7%)

1082	2*	Glu(66.7%), Ser(16.7%), Asp(8.3%), Ala(8.3%)
1083	1	Ser(58.3%), Gln(8.3%), Lys(8.3%), Gly(8.3%), Glu(8.3%), Ala(8.3%)
1084	1	Tyr(70.0%), Ser(10.0%), Glu(10.0%), Asp(10.0%)
1085	3*	Gln(69.2%), Arg(15.4%), Pro(7.7%), Glu(7.7%)
1086	1	Lys(53.8%), Ser(15.4%), Met(7.7%), Leu(7.7%), Glu(7.7%), Ala(7.7%)

ConSurf class, the conservation numbering scheme ranges from 1 (variable) to 9 (highly conserved); *, Unreliable estimate due to high number of gaps in alignment.
